# Copper-catalyzed formylation of alkenyl C–H bonds using BrCHCl_2_ as a stoichiometric formylating reagent†
†Electronic supplementary information (ESI) available. See DOI: 10.1039/c8sc00210j


**DOI:** 10.1039/c8sc00210j

**Published:** 2018-02-14

**Authors:** Yan Bao, Gao-Yin Wang, Ya-Xuan Zhang, Kang-Jie Bian, Xi-Sheng Wang

**Affiliations:** a Department of Chemistry , University of Science and Technology of China , 96 Jinzhai Road , Hefei , Anhui 230026 , China . Email: xswang77@ustc.edu.cn

## Abstract

The first example of copper-catalyzed direct formylation of alkenyl C–H bonds for the facile synthesis of α,β-unsaturated aldehydes has been developed. This transformation has demonstrated high reactivity, mild reaction conditions and a broad substrate scope. BrCHCl_2_ is expected to be developed as an efficient stoichiometric C1 building block in organic synthesis.

## Abstract 2

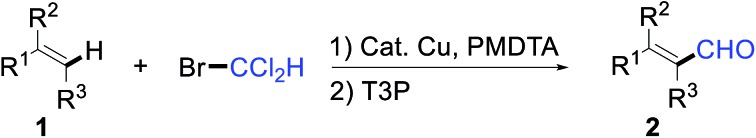
The first example of copper-catalyzed formylation of alkenyl C–H bonds with BrCHCl_2_ as a stoichiometric formylating reagent for the facile synthesis of α,β-unsaturated aldehydes has been developed.

## Introduction

Developing novel methods for the synthesis of complex molecules in an efficient and expeditious manner still remains a major challenge in organic synthesis. Accordingly, many novel strategies for the quick construction of complex molecular skeletons have been developed and used for the total synthesis of complex compounds.^1^,^2^ Among all of these strategies, domino reactions are emerging as efficient reactions in the facile synthesis of key intermediates. Of significant interest are cascade reactions that provide access to fundamental building blocks in a chemoselective manner from readily available starting materials in a step-economical fashion.^3^ As a key material used in cascade transformations, especially in organocatalyzed domino reactions, α,β-unsaturated aldehydes have been used in a broad range of unconventional transformations to make complex molecules or key intermediates.^4^ Thus, considerable efforts have been devoted to developing novel methods for the facile synthesis of α,β-unsaturated aldehydes from readily available simple raw materials in efficient and quick ways.^5^

**Scheme 1:**
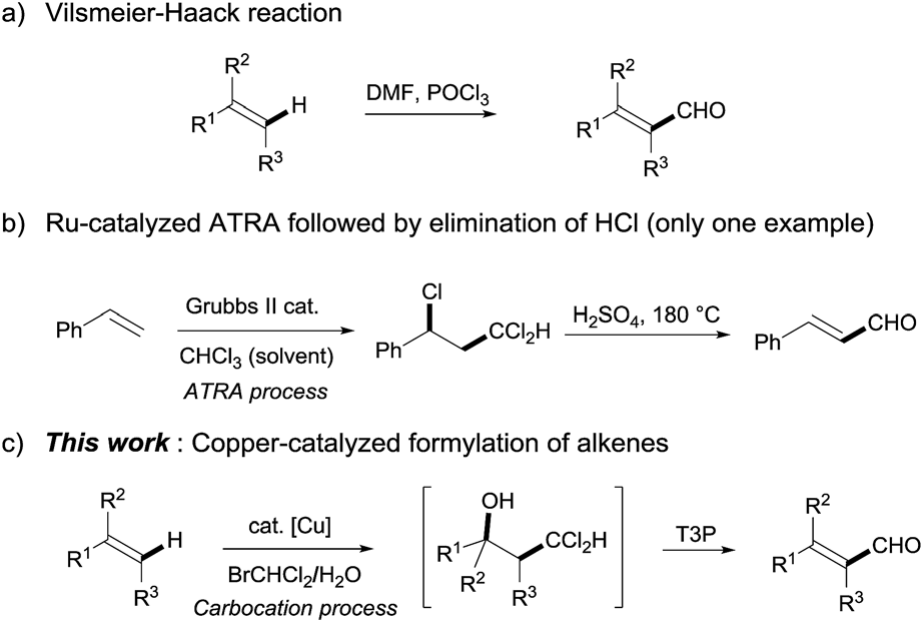
Facile synthesis of α,β-unsaturated aldehydes from alkenes.

As they are produced on a large scale industrially and have served as an important feedstock for the petrochemical industry, simple alkenes have long been realized as one of the most widely used raw materials for a great variety of organic transformations.^6^ The direct formylation of simple alkenes thus offers the most economical and efficient method for the facile construction of α,β-unsaturated aldehydes (Scheme 1). Indeed, the Vilsmeier–Haack reaction has long been developed as a useful approach to introduce a formyl group into alkenes.^7^ On the other hand, using the Grubbs II catalyst as the radical initiator succeeded in the atom transfer radical addition (ATRA) of styrene, which afforded the alkenyl aldehyde *via* the subsequent elimination of hydrochloric acid under strong acid conditions or a stoichiometric amount of silver salts at high temperature.^8^ Apart from this interesting progress, both known methods still suffer from a limited scope of alkenes and the requirement for a large amount of poisonous reagents (POCl_3_ as a precursor of the Vilsmeier reagent or CHCl_3_ as the solvent), which have definitely hampered their practical application in organic synthesis. Herein, we report a novel copper-catalyzed formylation of alkenyl C–H bonds for the facile synthesis of α,β-unsaturated aldehydes, in which high reactivity, mild conditions and a broad substrate scope have been demonstrated. The key to success is the use of commercially available BrCHCl_2_ as a stoichiometric formylating reagent instead of CHCl_3_, which is normally used as a solvent in such reactions. Alkenyl aldehydes were then furnished by the bifunctionalization of alkenes *via* a carbocation process and subsequent dehydration.

## Results and discussion

Our initial investigation commenced with 4-methoxystyrene (**1a**) as the model substrate, and bromodichloromethane as the stoichiometric formylating reagent in the presence of a catalytic amount of a copper catalyst (10 mol%) and 1.0 equiv. of PMDTA (1,1,4,7,7-pentamethyl-diethylenetriamine) in CH_3_CN at 40 °C (Table 1). Unfortunately, none of the desired product **2a** was obtained when CuBr was used as the catalyst. Considering that halogen atom exchange may improve the reactivity of CHBrCl_2_,^9^ to our delight, the addition of 1.0 equiv. of KI into the reaction system afforded **2a** successfully, albeit with a relatively low yield (23%, entry 2). A careful examination of copper catalysts was next performed, which indicated that Cu(OH)_2_ gave the best result with a 31% yield (entry 4). Meanwhile, further investigation into various solvents gave no improvement in yield and CH_3_CN was still the best choice. When a trace amount of the bifunctionalized alcohol **2aa** was isolated as a byproduct, it was conjectured that water acted as the hydroxyl source for this bifunctional reaction. As we expected, up to a 28% yield of **2aa** was obtained along with a 24% yield of **2a** when water was used directly as the solvent. To further improve the yield, co-solvents were investigated and CH_3_CN/H_2_O (1/1) gave the dichloromethylated alcohol **2aa** in 74% yield along with the aldehyde **2a** in 7% yield. After a simple work-up without purification and further dehydration with T3P, **2a** was finally furnished in 80% yield (entry 8). Considering the key role that PMDTA played in this transformation,^10^ other bases were also screened instead of PMDTA, but they did not give the desired product **2a** (Table 1, entries 11–13). Notably, by the replacement of KI with NaI, the yield of **2a** was further improved to 90% after this two-step formylation of 4-methoxystyrene (entry 15). Finally, control experiments indicated that none of the desired product was obtained without the addition of a copper catalyst or base (for details, see the ESI†).

**Table 1:** Copper-catalyzed formylation of 4-methoxystyrene: optimization of conditions^*a*^

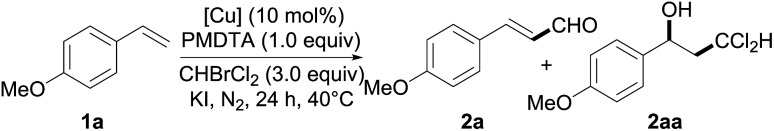				
Entry	[Cu]	Solvent(v/v)	Base	**2a**/**2aa** yield (%)^*b*^
1^*c*^	CuBr	CH_3_CN	PMDTA	0
2	CuBr	CH_3_CN	PMDTA	23/2
3	CuO	CH_3_CN	PMDTA	0
4	Cu(OH)_2_	CH_3_CN	PMDTA	31/2
5	Cu(OH)_2_	THF	PMDTA	0
6	Cu(OH)_2_	H_2_O	PMDTA	24/28
7	Cu(OH)_2_	DMSO	PMDTA	5/57
8	Cu(OH)_2_	CH_3_CN/H_2_O(1 : 1)	PMDTA	7/74(80)^*d*^
9	Cu(OH)_2_	CH_3_CN/DMSO(4 : 1)	PMDTA	8/72
10	Cu(OH)_2_	CH_3_CN/H_2_O(1 : 1)	—	0
11	Cu(OH)_2_	CH_3_CN/H_2_O(1 : 1)	Et_3_N	0
12	Cu(OH)_2_	CH_3_CN/H_2_O(1 : 1)	TMEDA	0
13	Cu(OH)_2_	CH_3_CN/H_2_O(1 : 1)	DMEDA	0
14^*e*^	Cu(OH)_2_	CH_3_CN/H_2_O(1 : 1)	PMDTA	10/72(82)^*d*^
15^*f*^	Cu(OH)_2_	CH_3_CN/H_2_O(1 : 1)	PMDTA	9/85(90)^*d*^

^*a*^Reaction conditions: **1a** (0.2 mmol, 1.0 equiv.), CHBrCl_2_ (3.0 equiv.), Cu catalyst (10 mol%), PMDTA (1.0 equiv.) and KI (1.0 equiv.) in CH_3_CN (1 mL) at 40 °C for 24 h.

^*b*^Isolated yield by ^1^H NMR analysis.

^*c*^KI was not added.

^*d*^The mixture of **2a**/**2aa** was dehydrated by T3P (1-propanephosphonic acid cyclic anhydride, 50% in ethyl acetate). The yield in the parentheses was the isolated yield of **2a**.

^*e*^TBAI was used instead of KI.

^*f*^NaI was used instead of KI. PMDTA: 1,1,4,7,7-pentamethyl-diethylenetriamine.

With the optimized conditions in hand, we next investigated the scope of the alkene (Table 2). Initially, the study of the substituent effect on the aryl rings of the styrene derivatives showed that both electron-donating groups, such as 4-methoxy, 4-phenoxy, 4-methyl and 4-methylthio (**1a–d**), and weak electron-withdrawing groups, such as fluorine, chlorine and bromine (**1f–h**), were all compatible with this catalytic system and were able to furnish the desired products in moderate to high yields. Not surprisingly, the substrates with electron-withdrawing groups installed on the aryl rings typically gave lower yields and required relatively high reaction temperatures (**2f–h**). Meanwhile, a variety of styrene derivatives with *ortho*- and *meta*- as well as *para*-substituents were smoothly formylated to afford the corresponding α,β-unsaturated aldehydes **2i–k** in acceptable yields.

**Table 2:** Substrate scope^*a*^

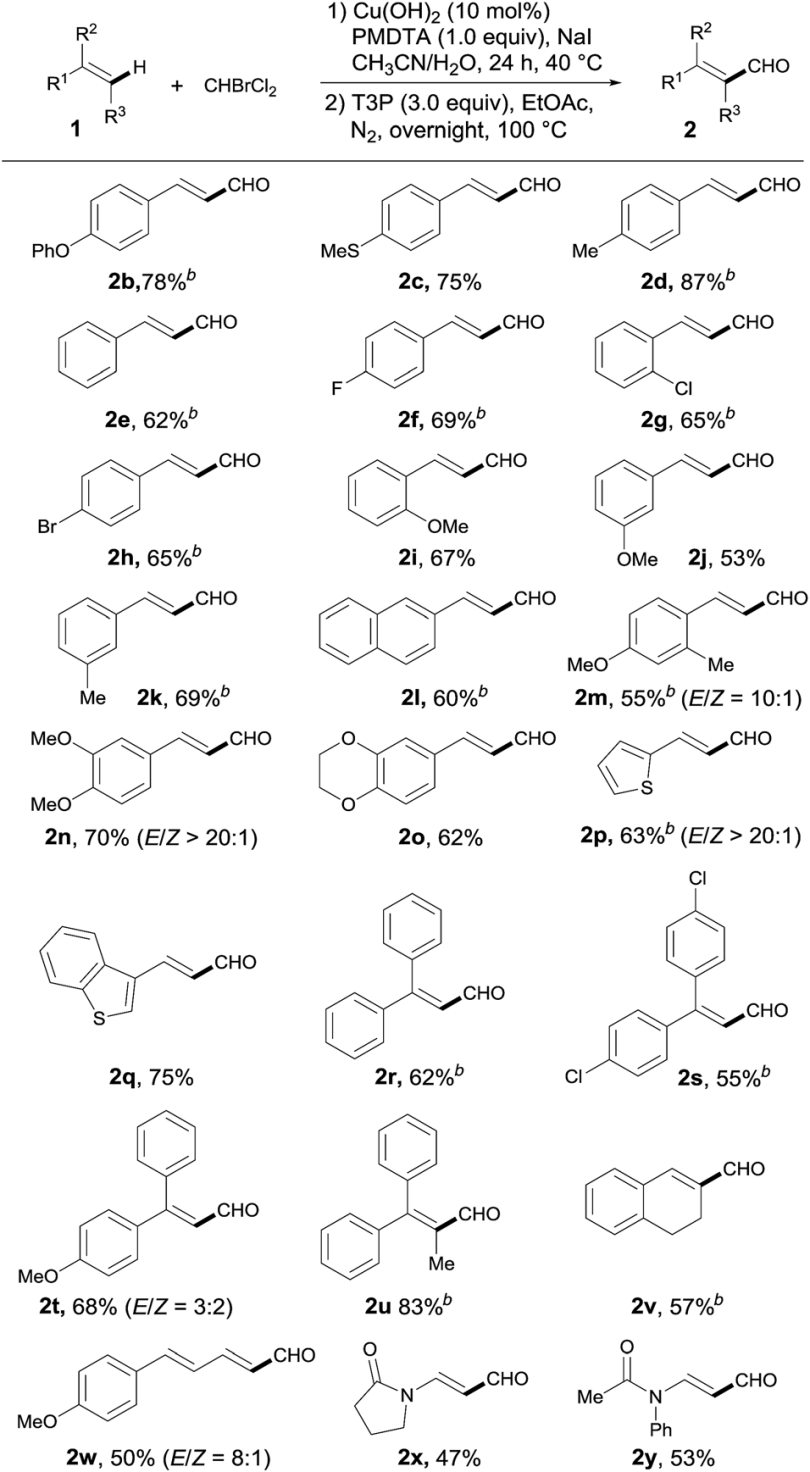

^*a*^General conditions: **1** (0.2 mmol), Cu(OH)_2_ (10 mol%), CHBrCl_2_ (3.0 equiv.), PMDTA (1.0 equiv.) and NaI (1.0 equiv.) in CH_3_CN/H_2_O (v/v = 1 : 1, 1 mL) at 40 °C for 24 h, then dehydration with T3P (3.0 equiv.) in EtOAc at 100 °C; for **2x–y**, **1** (0.2 mmol), Cu(hfacac)_2_·*x*H_2_O (10 mol%), CHBrCl_2_ (3.0 equiv.), PMDTA (1.0 equiv.) and KI (1.0 equiv.) in CH_3_CN (1 mL) at 80 °C for 24 h, and aldehydes **2x–y** were obtained directly. Isolated yields are reported.

^*b*^80 °C.

The *ortho*-chloro- and *meta*-methyl-substituted styrenes (**1g** and **1k**, respectively), styrene (**1e**) and 2-vinylnaphthalene (**1l**) also required a much higher temperature and gave moderate yields. Although the *ortho*- and *meta*-methoxystyrene (**1i** and **1j**, respectively) did not need high temperatures, the yields of the reactions were only moderate. Of interest to us was that heteroarene-derived styrene substrates, including 2-vinylthiophene **1p** and 3-vinylbenzothiophene **1q**, were also well-tolerated in this novel transformation with synthetically useful yields (**2p**, 63%; **2q**, 75%). Just as expected, 1,1-diphenylethenes **1r–t** normally worked as better radical trappers than styrene, and generated tri-substituted unsaturated aldehydes **2r–t** in relatively high yields (Table 2, **2r–t**). Of note was that the formylation of tri-substituted internal styrene **1u** also proceeded smoothly to give the corresponding tetra-substituted vinyl aldehyde with a fairly good yield (**2u**, 83%). Importantly, the subjection of 1,2-dihydronaphthalene **1v** to the optimized conditions resulted in the successful formylation of a C–H bond that was part of the cyclic double bond system. It was also noteworthy that the conjugated alkene **1w** underwent the reaction efficiently, with an acceptable yield (**2w**, 50%) and a good *E*/*Z* selectivity (8 : 1). To our satisfaction, *N*-vinyl substrates could also be directly formylated at the end of terminal alkenes to afford β-aminoacrylaldehydes in moderate yields, by simply altering the conditions to a combination of Cu(hfacac)_2_·*x*H_2_O, KI and CH_3_CN.^14^

**Scheme 2:**
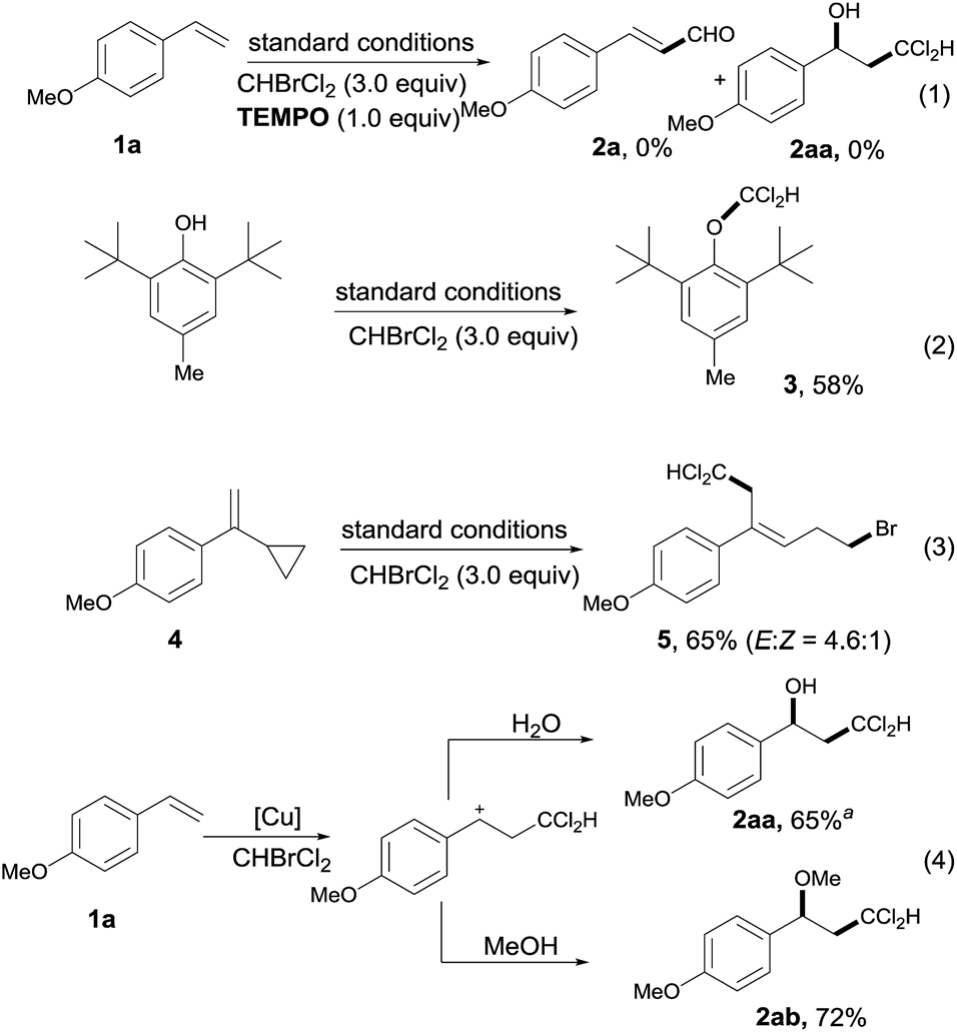
Mechanistic studies. ^*a*^ For details of the isolation of **2aa**, see the ESI.†

To gain some insights into the mechanism of this transformation, a series of control experiments were then performed accordingly (Scheme 2). First, the reaction was completely quenched when 1.0 equivalent of 2,2,6,6-tetramethyl-1-piperidinyloxy (TEMPO) was added to the standard conditions. Meanwhile, the addition of 1.0 equivalent of butylated hydroxytoluene (BHT) to the standard conditions afforded the corresponding dichloromethyl adduct **3** in 58% yield. Both results indicated that this transformation may proceed *via* a radical path. To further test this theory, the well known radical clock 1-(1-cyclopropylvinyl)-4-methoxybenzene **4** was synthesized and added into this catalytic system, furnishing the ring-opened product **5** in 65% yield, strengthening the hypothesis of the dichloromethyl radical (˙CCl_2_H) being involved in the catalytic cycle.

As alcohol **2aa** was isolated as the key intermediate along with the vinyl aldehyde **2a** in the model reaction (eqn (4), Scheme 2), it was suggested that a carbocation process was possibly involved in the catalytic cycle. Indeed, the replacement of H_2_O with methanol as the cation scavenger afforded the corresponding bifunctional product **2ab** in 72% yield, which further strengthened this possibility.^11^

**Scheme 3:**
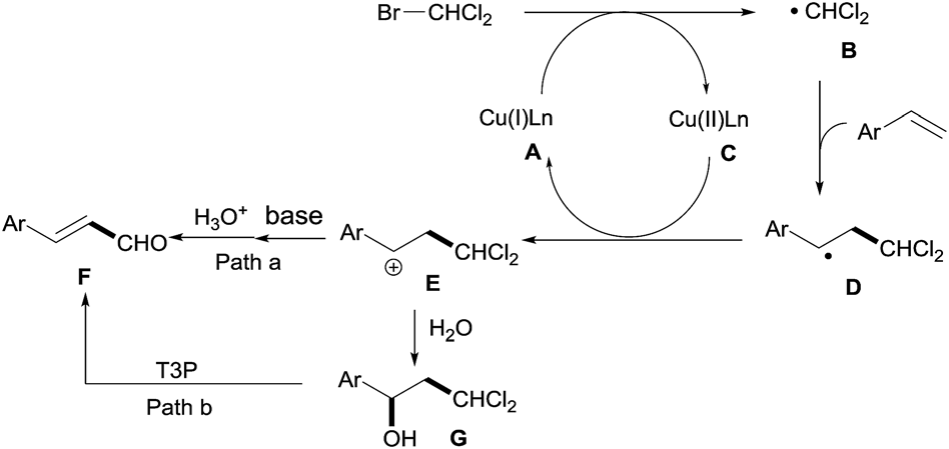
Proposed mechanism.

On the basis of our preliminary results and previous reports,^12^ a plausible mechanism involving a ˙CHCl_2_ radical is proposed as shown in Scheme 3. The copper salt is reduced to generate a low-valent Cu complex, which can transfer a single electron to CHBrCl_2_ and then produce the free radical compound ˙CHCl_2_ (**B**), and the radical subsequently reacts with the styrene. The ˙CHCl_2_ addition-compound **D** can be oxidized by a high-valent Cu complex to the cationic intermediate **E**. Finally, the high-valent Cu complex is reduced to a low-valent Cu complex to complete the catalytic cycle. There are two possible pathways for the sequential transformation of cation **E** to vinyl aldehyde **F**: Path a, which consists of deprotonation to regenerate the C=C bond, and **F** is obtained by the subsequent hydrolysis of the dichloromethyl group to the aldehyde; or Path b, where the cation **E** is captured by H_2_O to give the alcohol intermediate **G**, followed by dehydration with T3P and subsequent hydrolysis of the dichloromethyl group to afford **F**.^13^

**Scheme 4:**
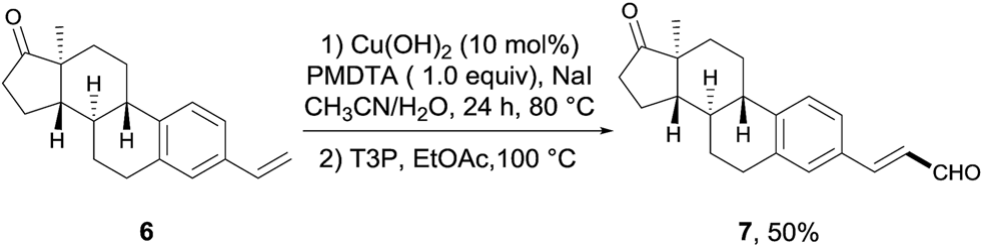
Formylation of the estrone derivative **6**.

To demonstrate the synthetic potential of this transformation, we applied this two-step transformation to the late-stage formylation of complex biologically active molecules. As shown in Scheme 4, the estrone-derived aryl alkene **6**, which was synthesized from estrone in two steps, was transformed to the desired vinyl aldehyde **7** in a 50% yield. This outcome clearly showed the great potential of this methodology as a facile strategy for the synthesis of various analogues or intermediates in drug discovery and screening.

## Conclusions

In summary, we have developed a novel copper-catalyzed formylation of alkenyl C–H bonds for the facile synthesis of α,β-unsaturated aldehydes. This transformation has demonstrated high reactivity, mild conditions and a broad substrate scope. An important point is that commercially available BrCHCl_2_ was used as a stoichiometric formylating reagent instead of CHCl_3_. Mechanistic studies indicated that alkenyl aldehydes were furnished by bifunctionalization of alkenes *via* a carbocation process and subsequent dehydration. Further applications of this method for the modification of bioactive molecules and the use of BrCHCl_2_ as an interesting C1 building block in organic synthesis are still underway in our laboratory.

## Conflicts of interest

There are no conflicts to declare.

## Supplementary Material

Supplementary information
