# Supplementary material for: Copper-catalyzed formylation of alkenyl C–H bonds using BrCHCl2 as a stoichiometric formylating reagent
Source: Chem Sci. 2018 Feb 14;9(11):2986–90. doi: 10.1039/c8sc00210j (PMC5915796; doi:10.1039/c8sc00210j)
Supplement: Supplementary file 1 [file SC-009-C8SC00210J-s001.pdf]

Electronic Supplementary Material (ESI) for Chemical Science.
This journal is © The Royal Society of Chemistry 2018

Supporting Information

For

Copper-catalyzed formylation of alkenyl C-H bonds with $BrCHCl_2$ used as a stoichiometric formylating reagent

Yan Bao, Gao-Yin Wang, Ya-Xuan Zhang, Kang-Jie Bian,
and Xi-Sheng Wang

Table of Contents

General Information S2

Tables of the Optimization of Reaction Conditions S3

Catalyst Screening S3
Other Conditions Screening S3
Solvent Screening S4
Mixed Solvent Screening S4
Base Screening S5
"I" Source Screening S5
Ratio of Aldehydes/Alcohols before Dehydration S6

Preparation of Substrates S7

General Procedure S7

Procedure of Modification of Estrone Derivative 6 S16

Mechanistic Studies S17

Dichloromethylation of 2-Phenylpropene S21

I/Br Exchange Experiments S22

References S23

NMR Spectra of New Compounds (^{1}H NMR, ^{13}C NMR) S24

General Information:

NMR spectra were recorded on Bruker-400 MHz NMR spectrometer (400 MHz for ^{1}H and 100 MHz for ^{13}C). ^{1}H NMR chemical shifts were determined relative to internal $(CH_3)_4Si$(TMS) at δ 0.0 or at the signal of a residual protonated solvent: $CDCl_3$ δ 7.26. ^{13}C NMR chemical shifts were determined relative to internal TMS at δ 0.0. Data for ^{1}H, ^{13}C NMR are recorded as follows: chemical shift (δ, ppm), multiplicity (s = singlet, d = doublet, t = triplet, m = multiplet, q = quartet, br = broad). Mass spectra were obtained on a mass spectrometer. High resolution mass spectra were recorded on P-SIMS-Gly of Bruker Daltonics Inc. using ESI-TOF (electrospray ionization-time of flight) or Micromass GCT using EI (electron impact). All reagents were purchased from TCI and *J&K* and used directly.

Tables of the Optimization of Reaction Condition

Table S1. Catalyst Screening[a]

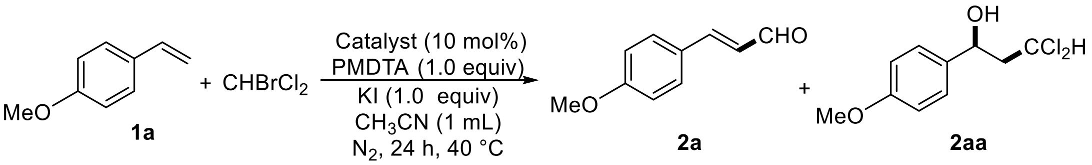

Entry	catalyst	**2a**+**2aa** yield (%)[b]	Entry	catalyst	**2a**+**2aa** yield (%)[b]
1	CuO	0	10	$Cu(ClO_4)_2{\bullet}6H_2O$	trace
2	CuF_2	25+1	11	$CuCl_2$	23+1
3	$Cu(PPh_3)_3Br$	trace	12	CuBr	23+2
4	$Cu(hfacac)_2{\bullet}XH_2O$	25+8	13	$FeBr_2$	trace
5	$Cu(acac)_2$	trace	14	$CoCl_2$	trace
**6**	**$Cu(OH)_2$**	**31+2**	15	$NiCl_2$	trace
7	$Cu(OTFA)_2{\bullet}XH_2O$	21+7	16	$PdCl_2$	trace
8	$CuF_2{\bullet}2H_2O$	24+2	17[c]	CuBr	0
9	$Cu(OAc)_2$	trace	18	/	0

[a] Unless otherwise noted, the reaction conditions were as follows: **1a** (0.2 mmol, 1.0 equiv), $CHBrCl_2$ (0.6 mmol, 3.0 equiv), Catalyst (0.02 mmol, 10 mol%), KI (0.2 mmol, 1.0 equiv) , PMDTA (0.2 mmol, 1.0 equiv), CH_3CN (1 mL), 40 °C, 24 h. [b] isolated yield by ^1H NMR analysis. [c] None KI was added. PMDTA=1,1,4,7,7-pentamethyldiethylenetriamin.

Table S2. Other Conditions Screening[a]

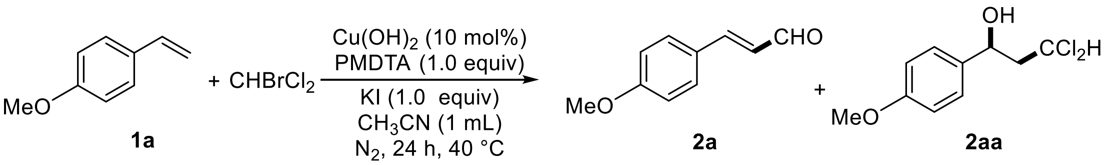

Entry	changes from the "standard conditions"	**2a**+**2aa** yield (%)[b]
1	35 °C	0
2	50 °C	28+2
3	CH_3CN (dry)	25+2
4	Ar	28+2
5	Air	0

[a] Unless otherwise noted, the reaction conditions were as follows: **1a** (0.2 mmol, 1.0 equiv), $CHBrCl_2$ (0.6 mmol, 3.0 equiv), $Cu(OH)_2$ (0.02 mmol, 10 mol%), KI (0.2 mmol, 1.0 equiv), PMDTA (0.2 mmol, 1.0 equiv), CH_3CN (1 mL), 40 °C, 24 h. [b] Isolated yield by ^1H NMR analysis. PMDTA=1,1,4,7,7-pentamethyl-diethylenetriamin.

Table S3. Solvent Screening[a]

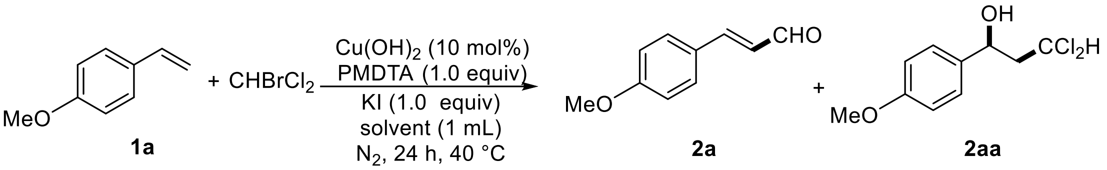

Entry	solvent	**2a**+**2aa** yield (%)[b]	Entry	solvent	**2a**+**2aa** yield (%)[b]
1	Cyclohexane	trace	10	Dioxane	0
2	THF	0	11	toulene	0
3	Et_2O	0	12	DMF	29+11
4	acetone	trace	**13**	**DMSO**	**5+ 57**
5	CH_3OH	0	14	NMP	0
6	EA	trace	15	PhCl	0
7	CCl_4	trace	**16**	**H_2O**	**24+28**
8	1,2-Dimethoxyethane	trace	17	PhCN	0
9	CH_3NO_2	0			

[a] Unless otherwise noted, the reaction conditions were as follows: **1a** (0.2 mmol, 1.0 equiv), $CHBrCl_2$ (0.6 mmol, 3.0 equiv), $Cu(OH)_2$ (0.02 mmol, 10 mol%), KI (0.2 mmol, 1.0 equiv), PMDTA (0.2 mmol, 1.0 equiv), solvent (1 mL), 40 °C, 24 h. [b] isolated yield by ^1H NMR analysis. PMDTA=1,1,4,7,7-pentamethyl-diethylenetriamin.

Table S4. The mixed solvent Screening[a]

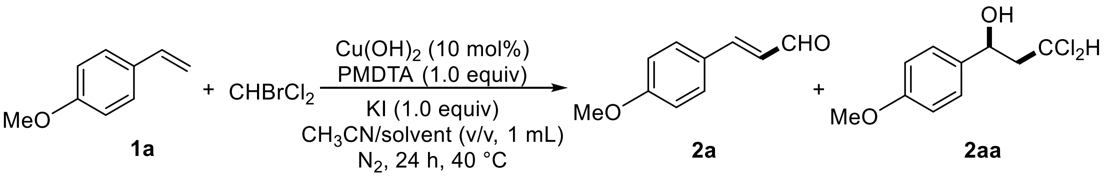

Entry	solvent (v/v)	**2a**+**2aa** yield (%)[b]
1	DMSO(1/1)	5+64
2	DMSO(2/1)	8+65
3	DMSO(4/1)	8+72
4	DMSO(9/1)	14+59
**5**[c]	**H_2O(1/1)**	**7+74 (80)**
6	H_2O(4/1)	8+63
7	H_2O(9/1)	14+44

[a] Unless otherwise noted, the reaction conditions were as follows: **1a** (0.2 mmol, 1.0 equiv), $CHBrCl_2$ (0.6 mmol, 3.0 equiv), $Cu(OH)_2$ (0.02 mmol, 10 mol%), KI (0.2 mmol, 1.0 equiv), PMDTA (0.2 mmol, 1.0 equiv), solvent (1 mL), 40 °C, 24 h. [b] isolated yield by ^1H NMR analysis. PMDTA=1,1,4,7,7-pentamethyl-diethylenetriamin. [c] **2a+2aa** was dehydrated by T3P (1-Propanephosphonic acid cyclic anhydride 50% ethyl acetate). The yield in the parentheses was isolated yield of **2a**.

Table S5 Base Screening[a]

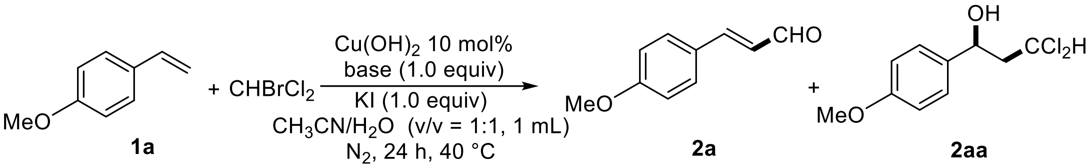

1a **2a** **2aa**

Entry	base	**2a**+**2aa** yield (%)
1	none	0
2	Et_3N (2.0 equiv)	0
3	TMEDA (1.0 equiv)	0
4	DMEDA (1.0 equiv)	0

[a] Unless otherwise noted, the reaction conditions were as follows: **1a** (0.2 mmol, 1.0 equiv), $CHBrCl_2$ (0.6 mmol, 3.0 equiv), $Cu(OH)_2$ (0.02 mmol, 10 mol%), KI (0.2 mmol, 1.0 equiv), PMDTA (0.2 mmol, 1.0 equiv), 40 °C, 24 h. PMDTA=1,1,4,7,7-pentamethyl-diethylenetriamin.

Table S6 "I" source Screening[a]

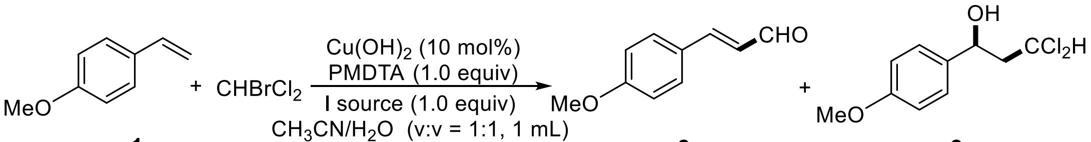

1a **2a** **2aa**

Entry	I source	**2a**+**2aa** yield (%)[b]
**1**	**NaI (1.0 equiv)**	**9+85 (90%)**
2	TBAI (1.0 equiv)	10+72 (82%)

[a] Unless otherwise noted, the reaction conditions were as follows: **1a** (0.2 mmol, 1.0 equiv), $CHBrCl_2$ (0.6 mmol, 3.0 equiv), $Cu(OH)_2$ (0.02 mmol, 10 mol%), "I" source (0.2 mmol, 1.0 equiv), PMDTA (0.2 mmol, 1.0 equiv), solvent (1 mL), 40 °C, 24 h. PMDTA=1,1,4,7,7-pentamethyl-diethylenetriamin. [b] isolated yield by ^1H NMR analysis. **2a+2aa** was dehydrated by T3P (1-Propanephosphonic acid cyclic anhydride 50% ethyl acetate). The yield in the parentheses was isolated yield of **2a**.

Table S7 Ratio of **2**/**2'** before Dehydration[a]

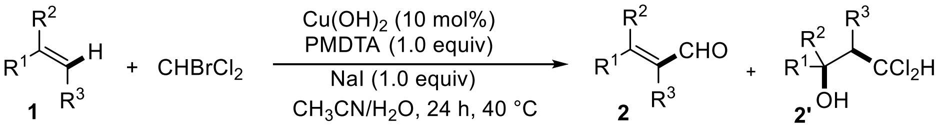

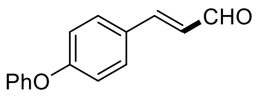

2b/2ab, 7%/74%[b]

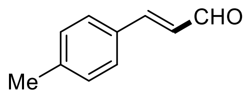

2c/2ac, 3%/78[b]

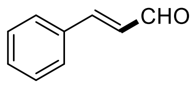

2e/2ae, 5%/53%[b]

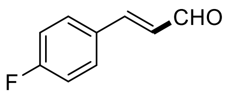

2f/2af, 6%/54%[b]

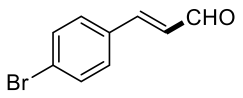

2h/2ah, 3%/59%[b]

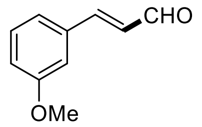

2h/2ah, 6%/52%[b]

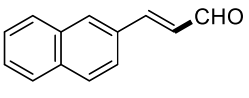

2l/2al, 11%/51%[b]

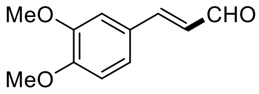

2l/2al, 8%/67%

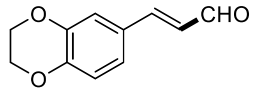

2o/2ao, 9%/80%

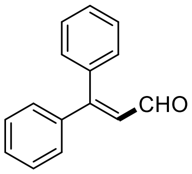

2r/2ar, 15%/46%[b]

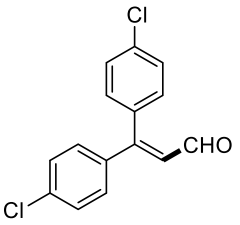

2s/2as, 23%/30%[b]

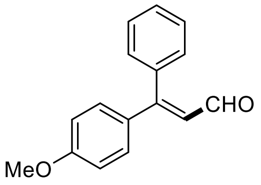

2t/2at, 49%/23%[b] (*E*/*Z* = 3:1)

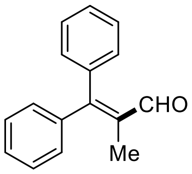

2u/2au, 44%/40%[b]

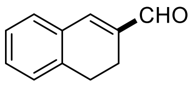

2v/2av, 18%/52%[b]

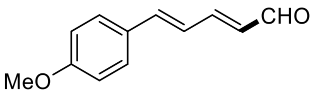

2w/2aw, 9%/43% (*E*/*Z* = 4:1)

[a] Unless otherwise noted, the reaction conditions were as follows: **1** (0.2 mmol, 1.0 equiv), $CHBrCl_2$ (0.6 mmol, 3.0 equiv), $Cu(OH)_2$ (0.02 mmol, 10 mol%), NaI (0.2 mmol, 1.0 equiv), PMDTA (0.2 mmol, 1.0 equiv), solvent (1 mL), 40 °C, 24 h. [b] 80 °C. The ^1H NMR yields ousing dibromomethane as an internal standard.

Preparation of Substrates.

Substrates **1b**[1], **1f**[2], **1m-o**[3], **1q-s**[3] , **1u-w**[4], **1y**[5] and **6**[6] were prepared according to the known methods. Substrates **1a**, **1c-e**, **1p**, **1t** and **1x** were purchased from TCI, J&K *et al*. and used as received.

General Procedure

To a 50 mL of Schlenk tube was added $Cu(OH)_2$ (0.02 mmol, 10 mol%) under air, followed by NaI (0.2 mmol, 1.0 equiv). The mixture was then evacuated and back filled with N_2 (3 times). 4-Methoxystrene **1a** (0.2 mmol, 1.0 equiv), bromodichoromethane (0.6 mmol, 3.0 equiv), PMDTA (0.2 mmol, 1.0 equiv) and CH_3CN/H_2O (v/v = 1/1, 1 mL) were added subsequently. The Schlenk tube was screw capped and put into a preheated oil bath (40 ˚C). After stirring for 24 hours, the reaction mixture was cooled to room temperature, and then extracted with ethyl acetate for 3 times. After the solvent was removed under rotary evaporation, the residue was resolved in ethyl acetate (5 mL) and T3P (1-Propanephosphonic acid cyclic anhydride 50% ethyl acetate, 0.6 mmol, 3.0 equiv) was added. The mixture was stirred at 100 ˚C overnight, and the reaction was quenched by water (5 mL) and stirred for a few more minutes, extracted with ethyl acetate for 3 times. The solvent was removed under rotary evaporation, and the residue was then purified by flash column chromatography (petroleum ether/ethyl acetate = 6:1) to give **2a** as pale yellow liquid.

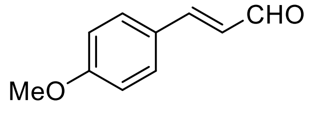

2a

The product **2a** was obtained with flash column chromatography (petroleum ether/ethyl acetate = 6:1) as

pale yellow liquid (90% yield). ^{1}H NMR (400 MHz, $CDCl_3$) δ 9.65 (d, *J* = 8.0 Hz, 1H), 7.54-7.50 (m, 2H), 7.42 (d, *J* = 8.0 Hz, 1H), 6.94 (d, *J* = 8.0 Hz, 2H), 6.61 (dd, *J* = 16.0, 8.0 Hz, 1H), 3.85 (s, 3H). ^{13}C NMR (101 MHz, $CDCl_3$) δ 193.6, 162.3, 152.9, 130.5, 126.9, 126.6, 114.1, 55.6. HRMS ESI (m/z): $[M+H]^+$ calcd. for $C_{10}H_{11}O_2$: 163.0759, found: 163.0762.

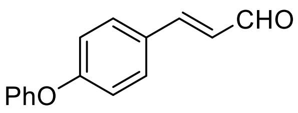

2b

The product **2b** was obtained with flash column chromatography (petroleum ether/ethyl acetate = 10:1) as pale yellow liquid (69% yield). ^{1}H NMR (400 MHz, $CDCl_3$) δ 9.67 (d, *J* = 8.0 Hz, 1H), 7.54 (dd, *J* = 8.0, 4.0 Hz, 2H), 7.44 (d, *J* = 16.0 Hz, 1H), 7.39 (t, *J* = 8.0 Hz, 2H), 7.19 (t, *J* = 8.0 Hz, 1H), 7.07 (d, *J* = 8.0 Hz, 2H), 7.02 (d, *J* = 12.0 Hz, 2H), 6.64 (dd, *J* = 16.0, 8.0 Hz, 1H). ^{13}C NMR (101 MHz, $CDCl_3$) δ 193.6, 160.6, 155.8, 152.3, 130.5, 130.1, 128.7, 127.5, 124.6, 120.1, 118.4. HRMS ESI (m/z): $[M+H]^+$ calcd. for $C_{15}H_{13}O_2$: 225.0916, found: 225.0915.

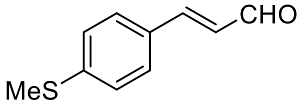

2c

The product **2c** was obtained with flash column chromatography (petroleum ether/ethyl acetate = 6:1) as pale yellow liquid (75% yield). ^{1}H NMR (400 MHz, $CDCl_3$) δ 9.67 (d, *J* = 8.0 Hz, 1H), 7.47 (d, *J* = 8.0 Hz, 2H), 7.41 (d, *J* = 16.0 Hz, 1H), 7.25 (d, *J* = 8.0 Hz, 2H), 6.67 (dd, *J* = 16.0, 8.0 Hz, 1H), 2.51 (s, 3H). ^{13}C NMR (101 MHz, $CDCl_3$) δ 193.8, 152.4, 143.7, 130.5, 128.9, 127.7, 126.0, 15.1 HRMS ESI (m/z): $[M+H]^+$ calcd. for $C_{10}H_{11}OS$: 179.0531, found: 179.0522.

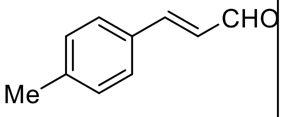

2d

The product **2d** was obtained with flash column chromatography (petroleum ether/ethyl acetate = 6:1) as pale

yellow liquid (87% yield). ^{1}H NMR (400 MHz, $CDCl_3$) δ 9.68 (d, *J* = 8.0 Hz, 1H), 7.48-7.43 (m, 3H), 7.24 (d, *J* = 8.0 Hz, 2H), 6.69 (dd, *J* = 16.0, 8.0 Hz, 1H), 2.40 (s, 3H). ^{13}C NMR (101 MHz, $CDCl_3$) δ 194.0, 153.2, 142.1, 131.4, 130.0, 128.7, 127.8, 21.7. HRMS ESI (m/z): $[M+H]^+$ calcd. for $C_{10}H_{11}O$: 147.0810, found: 147.0810.

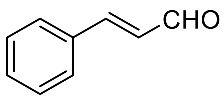

2e

The product **2e**[7] was obtained with flash column chromatography (petroleum ether/ethyl Acetate = 10:1) as pale yellow liquid (78% yield).

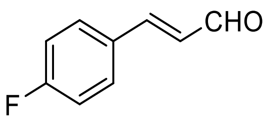

2f

The product **2f** was obtained with flash column chromatography (petroleum ether/ethyl acetate= 6:1) as pale yellow liquid (69% yield). ^{1}H NMR (400 MHz, $CDCl_3$) δ 9.68(d, *J* = 8.0 Hz, 1H), 7.57(dd, *J*= 8.0, 4.0 Hz, 2H), 7.34 (d, *J* = 16.0 Hz, 1H), 7.12 (t, *J* = 8.0 Hz, 2H), 6.64 (dd, *J* = 16.0, 8.0 Hz, 1H). ^{13}C NMR (101 MHz, $CDCl_3$) δ 193.6, 164.5 (d, *J* = 254.5 Hz), 151.5, 130.6 (d, *J* = 8.0 Hz), 130.4 (d, *J* = 4.0 Hz), 128.4 (d, *J* = 2.0 Hz), 116.5 (d, *J* = 22.0 Hz). HRMS ESI (m/z): $[M+H]^+$ calcd. for C_9H_8OF: 151.0559, found: 151.0561.

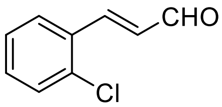

2g

The product **2g** was obtained with flash column chromatography (petroleum ether/ethyl acetate = 7:1) as pale yellow liquid (65% yield). ^{1}H NMR (400 MHz, $CDCl_3$) δ 9.77 (d, *J* = 8.0 Hz, 1H), 7.95 (d, *J* = 16.0 Hz, 1H), 7.67 (dd, *J* = 8.0 4.0 Hz, 1H), 7.47 (dd, *J* = 8.0, 4.0 Hz, 1H), 7.40-7.31 (m, 2H), 6.71 (dd, *J* = 16.0, 8.0 Hz, 1H). ^{13}C NMR (101 MHz, $CDCl_3$) δ 193.8, 148.2, 135.4, 132.2, 132.1, 130.7, 130.5, 128.0, 127.5. HRMS ESI (m/z): $[M+H]^+$ calcd. for C_9H_8ClO: 167.0264, found: 167.0259.

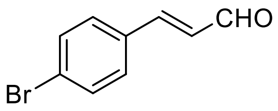

2h

The product **2h** was obtained with flash column chromatography (petroleum ether/ethyl acetate = 7:1) as pale yellow liquid (65% yield). ^{1}H NMR (400 MHz, $CDCl_3$) δ 9.71 (d, *J* = 8.0 Hz, 1H), 7.57 (d, *J* = 8.0 Hz, 2H), 7.44-7.40 (m, 3H), 6.70 (dd, *J* = 16.0, 8.0 Hz, 1H). ^{13}C NMR (101 MHz, $CDCl_3$) δ 193.6, 151.3, 133.0, 132.6, 129.9, 129.1, 125.9. HRMS ESI (m/z): $[M+H]^+$ calcd. for C_9H_8OBr: 210.9759, found: 210.9759.

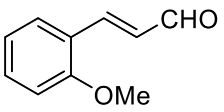

2i

The product **2i**[7] was obtained with flash column chromatography (petroleum ether/ethyl acetate = 8:1) as pale yellow liquid (67% yield).

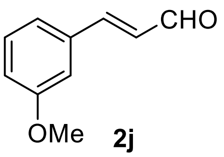

2j

The product **2j** was obtained with flash column chromatography (petroleum ether/ethyl acetate = 7:1) as pale yellow liquid (53% yield). ^{1}H NMR (400 MHz, $CDCl_3$) δ 9.70 (d, *J* = 8.0 Hz, 1H), 7.46 (d, *J* = 16.0 Hz, 1H), 7.35 (t, *J* = 8.0 Hz, 1H), 7.16 (d, *J* = 8.0 Hz, 1H), 7.08 (s, 1H), 7.00 (dd, *J* = 8.0, 4.0 Hz, 1H), 6.71 (dd, *J* = 16.0, 8.0 Hz, 1H), 3.85 (s, 3H). ^{13}C NMR (101 MHz, $CDCl_3$) δ 193.9, 160.1, 152.9, 135.4, 130.3, 129.0, 121.4, 117.2, 113.4, 55.5. HRMS ESI (m/z): $[M+H]^+$ calcd. for $C_{10}H_{11}O_2$: 163.0759, found: 163.0788.

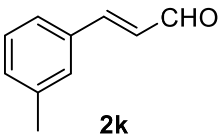

2k

The product **2k** was obtained with flash column chromatography (petroleum ether/ethyl acetate = 7:1) as pale yellow liquid (69% yield). ^{1}H NMR (400 MHz, $CDCl_3$) δ 9.70 (d, *J* = 8.0 Hz, 1H), 7.46 (d, *J* = 12.0 Hz, 1H), 7.38-7.37 (m, 2H), 7.31 (t, *J* = 12.0 Hz,

1H), 7.26 (d, *J* = 8.0 Hz, 1H), 6.71 (dd, *J* = 16.0, 8.0 Hz, 1H), 2.40 (s, 3H). ^{13}C NMR (101 MHz, $CDCl_3$) δ 194.0, 153.3, 139.0, 134.1, 132.3 129.3, 129.1, 128.6, 125.9, 21.5. HRMS ESI (m/z): $[M+H]^+$ calcd. for $C_{10}H_{11}O$: 147.0810, found: 147.0809.

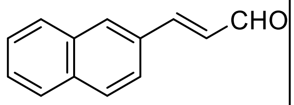

2l

The product **2l** was obtained with flash column chromatography (petroleum ether/ethyl acetate = 10:1) as white solid (78% yield). ^{1}H NMR (400 MHz, $CDCl_3$) δ 9.76 (d, *J* = 8.0 Hz, 1H), 7.98 (s, 1H), 7.90-7.85 (m, 3H), 7.69-7.60 (m, 2H), 7.55(s, 2H), 6.83 (dd, *J* = 16.0, 8.0 Hz, 1H). ^{13}C NMR (101 MHz, $CDCl_3$) δ 193.8, 152.9, 134.8, 133.3, 131.7, 130.8, 129.1, 128.9, 128.8, 128.0, 127.9, 127.1, 123.6. HRMS ESI (m/z): $[M+H]^+$ calcd. for $C_{13}H_{11}O$: 183.0810, found: 183.0811.

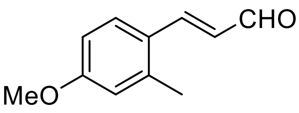

2m(*E/Z* =10:1)

The product **2m** was obtained with flash column chromatography (petroleum ether/ethyl acetate = 10:1) as white solid (55% yield). **2m** (*E*): ^{1}H NMR (400 MHz, $CDCl_3$) δ 9.64 (d, *J* = 8.0 Hz, 1H), 7.42-7.36 (m, 3H), 6.86 (d, *J* = 8.0 Hz, 1H), 6.61 (dd, *J* = 16.0, 8.0 Hz, 1H), 3.88(s, 3H), 2.24 (s, 3H). **2m** (*Z*): ^{1}H NMR (400 MHz, $CDCl_3$) δ 9.64 (d, *J* = 8.0 Hz, 1H), 7.70 (d, *J* = 16.0 Hz, 2H), 7.42-7.36 (m, 1H), 6.83 (d, *J* = 8.0 Hz, 1H), 6.31 (d, *J* = 16.0 Hz, 1H), 3.87(s, 3H), 2.23 (s, 3H). ^{13}C NMR (101 MHz, $CDCl_3$) δ 194.0, 160.7, 153.4, 130.8, 128.7, 127.8, 126.44, 126.41, 110.2, 55.7, 16.4. HRMS ESI (m/z): $[M+H]^+$ calcd. for $C_{11}H_{13}O_2$: 177.0916, found: 177.0916.

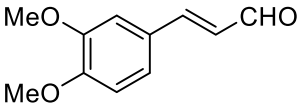

2n (*E/Z*>20:1)

The product **2m** was obtained with flash column chromatography (petroleum ether/ethyl acetate = 3:1) as

white solid (70% yield). ^{1}H NMR (400 MHz, $CDCl_3$) δ 9.66 (d, *J* = 8.0 Hz, 1H), 7.41 (d, *J* = 16.0 Hz, 1H), 7.16 (dd, *J* = 8.0, 1.6 Hz, 1H), 7.08 (s, 1H), 6.90 (d, *J* = 8.0 Hz, 1H), 6.61 (dd, *J* = 16.0, 8.0 Hz, 1H), 3.93 (s, 3H), 3.92 (s, 3H). ^{13}C NMR (101 MHz, $CDCl_3$) δ 193.7, 153.0, 152.1, 149.5, 127.2, 126.8, 123.6, 112.2, 109.9, 56.2, 56.1. HRMS ESI (m/z): $[M+H]^+$ calcd. for $C_{11}H_{13}O_3$: 193.0865, found: 193.0863.

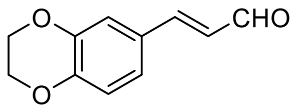

2o

The product **2o** was obtained with flash column chromatography (petroleum ether/ethyl acetate = 7:1) as pale yellow liquid (53% yield). ^{1}H NMR (400 MHz, $CDCl_3$) δ 9.65 (d, *J* = 8.0 Hz, 1H), 7.36 (dd, *J* = 16.0 Hz, 1H), 7.11-7.07 (m, 2H), 6.91 (d, *J* = 8.0 Hz, 1H), 6.60 (dd, *J* = 16.0, 8.0 Hz, 1H), 4.33-4.28 (m, 4H). ^{13}C NMR (101 MHz, $CDCl_3$) δ 193.8, 152.8, 146.7, 144.0, 127.8, 127.2, 122.8, 118.1, 117.3, 64.8, 64.3. HRMS ESI (m/z): $[M+H]^+$ calcd. for $C_{11}H_{11}O_3$: 191.0708, found: 191.0710.

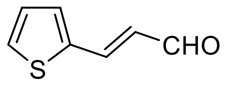

2p (*E/Z*>*20:1*)

The product **2p** was obtained with flash column chromatography (petroleum ether/ethyl acetate = 10:1) as yellow liquid (63% yield). ^{1}H NMR (400 MHz, $CDCl_3$) δ 9.61 (d, *J* = 8.0 Hz, 1H), 7.58 (d, *J* = 16.0 Hz, 1H), 7.50 (d, *J* = 4.0 Hz, 1H), 7.36 (d, *J* = 4.0 Hz, 1H), 7.11 (d, *J* = 4.0 Hz, 1H), 6.51 (dd, *J* = 16.0, 8.0 Hz, 1H). ^{13}C NMR (101 MHz, $CDCl_3$) δ 193.1, 144.6, 139.4, 132.2, 130.5, 128.7, 127.4. HRMS ESI (m/z): $[M+H]^+$ calcd. for C_7H_7OS:139.0218, found: 139.0216.

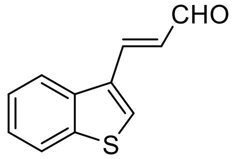

2q

The product **2q** was obtained with flash column chromatography (petroleum ether/ethyl acetate = 8:1) as yellow liquid (75% yield). ^{1}H NMR (400 MHz, $CDCl_3$) δ

9.75 (d, J = 8.0 Hz, 1H), 8.02 (d, J = 8.0 Hz, 1H), 7.92-7.90 (m, 2H), 7.52 (d, J = 8.0 Hz, 1H), 7.53-7.43 (m, 2H), 6.84 (dd, J = 16.0, 8.0Hz, 1H). ^{13}C NMR (101 MHz, $CDCl_3$) δ 194.0, 144.1, 140.7, 136.9, 131.5, 130.3, 129.0, 125.5, 125.4, 123.3, 122.0. HRMS ESI (m/z): $[M+H]^+$ calcd. for $C_{11}H_9OS$: 189.0374, found: 189.0375.

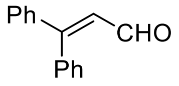

2r

The product **2r** was obtained with flash column chromatography (petroleum ether/ethyl acetate = 10:1) as yellow solid (62% yield). ^{1}H NMR (400 MHz, $CDCl_3$) δ 9.54 (d, J = 8.0 Hz, 1H), 7.49-7.30 (m, 10H), 6.61 (d, J = 8.0 Hz, 1H). ^{13}C NMR (101 MHz, $CDCl_3$) δ 193.7, 162.1, 139.8, 136.8, 130.9, 130.6, 129.6, 128.8, 128.7, 128.5, 127.4. HRMS ESI (m/z): $[M+H]^+$ calcd. for $C_{15}H_{13}O$: 209.0966, found: 209.0965.

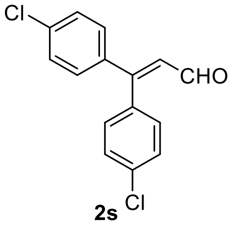

2s

The product **2s** was obtained with flash column chromatography (petroleum ether/ethyl acetate = 10:1) as yellow solid (55% yield). ^{1}H NMR (400 MHz, $CDCl_3$) δ 9.54 (d, J = 8.0 Hz, 1H), 7.45 (d, J = 8.0 Hz, 2H), 7.37 (d, J = 16.0 Hz, 2H), 7.28-7.23 (m, 4H), 6.56 (d, J = 8.0 Hz, 1H). ^{13}C NMR (101 MHz, $CDCl_3$) δ 192.8, 159.6, 137.9, 137.1, 136.2, 134.7, 132.1, 130.0, 129.2, 129.0, 127.8. HRMS ESI (m/z): $[M+H]^+$ calcd. for $C_{15}H_{11}Cl_2O$: 277.0187, found: 277.0187.

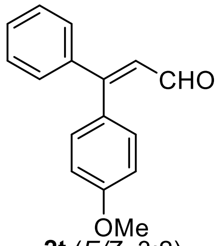

2t (*E/Z*=3:2)

The product **2t** was obtained with flash column chromatography (Petroleum ether/ethyl acetate = 10:1) as yellow solid (68% yield). **2t** (*E*): ^{1}H NMR (400 MHz, $CDCl_3$) δ 9.56 (d, J = 8.0 Hz, 1H), 7.46-7.23 (m,

7H), 6.96 (d, J = 8.0 Hz, 2H), 6.52 (d, J = 8.0 Hz, 1H), 3.88 (s, 3H). ^{13}C NMR (101 MHz, $CDCl_3$) δ 193.7, 162.5, 161.0, 132.7, 130.8, 130.6, 130.5, 129.1, 128.7, 127.2, 113.9, 55.6. **2t** (*Z*): ^1H NMR (400 MHz, $CDCl_3$) δ 9.45 (d, J = 8.0Hz, 1H), 7.46-7.23 (m, 7H), 6.81 (d, J = 8.0 Hz, 2H), 6.57 (d, J=8.0 Hz, 1H) 3.84 (s, 3H). ^{13}C NMR (101 MHz, $CDCl_3$) δ 193.8, 162.2, 161.8, 140.4, 137.0, 132.0, 129.5, 129.1, 128.4, 125.7, 114.2, 55.6. HRMS ESI (m/z): $[M+H]^+$ calcd. for $C_{16}H_{15}O_2$: 239.1072, found: 239.1071.

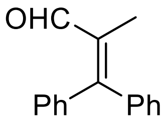

2u

The product **2u** was obtained with flash column chromatography (petroleum ether/ethyl acetate = 6:1) as white solid (83% yield). ^1H NMR (400 MHz, $CDCl_3$) δ 9.59 (s, 1H), 7.41-7.33 (m, 6H), 7.20-7.16 (m, 4H), 1.97 (s, 3H). ^{13}C NMR (101 MHz, $CDCl_3$) δ 194.5, 159.6, 140.9, 139.0, 135.4, 131.1, 129.8, 129.1, 128.8, 128.3, 128.2, 14.4. HRMS ESI (m/z): $[M+H]^+$ calcd. for $C_{16}H_{15}O$: 223.1123, found: 223.1122.

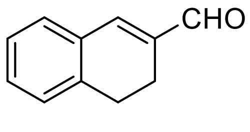

2v

The product **2v** was obtained with flash column chromatography (petroleum ether/ethyl acetate = 7:1) as colorless liquid (57% yield). ^1H NMR (400 MHz, $CDCl_3$) δ 9.66 (s, 1H), 7.32-7.19 (m, 5H), 2.87 (t, J = 8.0 Hz, 2H), 2.56 (t, J = 8.0 Hz, 2H). ^{13}C NMR (101 MHz, $CDCl_3$) δ 193.0, 145.9, 139.3, 138.2, 132.2, 130.7, 129.0, 128.2, 127.0, 27.1, 19.3. HRMS ESI (m/z): $[M+H]^+$ calcd. for $C_{11}H_{11}O$: 159.0810, found: 159.0810.

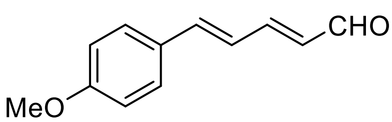

2w (*E*/*Z* =8:1)

The product **2w** was obtained with flash column chromatography (petroleum ether/ethyl acetate =

7:1) as yellow liquid (50% yield). **2w** (*E*): ^{1}H NMR (400 MHz, $CDCl_3$) δ 9.59 (d, *J* = 8.0 Hz, 1H), 7.46 (d, *J* = 12.0 Hz, 2H), 7.26 (dd, *J* = 8.0, 1.6 Hz, 1H), 6.99-6.85 (m, 4H), 6.23 (dd, *J* = 16.0, 8.0 Hz, 1H), 3.84 (s, 3H). ^{13}C NMR (101 MHz, $CDCl_3$) δ 193.8, 161.0, 152.9, 142.5, 130.7, 129.3, 128.5, 124.2, 114.5, 55.5. **2w** (*Z*): ^{1}H NMR (400 MHz, $CDCl_3$) δ 9.65 (d, *J* = 8.0 Hz, 1H), 7.53 (d, *J* = 12.0 Hz, 2H), 7.45 (dd, *J* = 8.0, 1.6 Hz, 1H), 6.99-6.85 (m, 4H), 6.61 (dd, *J* = 16.0, 8.0Hz, 1H), 3.86 (s, 3H). HRMS ESI (m/z): $[M+H]^+$ calcd. for $C_{12}H_{13}O_2$: 189.0916, found: 189.0916.

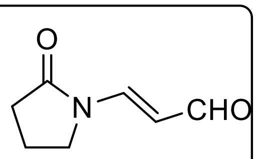

2x

The product **2x** was obtained with flash column chromatography (petroleum ether/ethyl acetate = 3:1) as yellow solid (47% yield). ^{1}H NMR (400 MHz, $CDCl_3$) δ 9.48 (d, *J* = 8.0 Hz, 1H), 7.89 (d, *J* = 12.0 Hz, 1H), 5.56 (dd, *J* = 16.0, 8.0 Hz, 1H), 3.62 (t, *J* = 8.0 Hz, 2H), 2.60 (t, *J* = 8.0 Hz, 2H), 2.24-2.18 (m, 2H). ^{13}C NMR (101 MHz, $CDCl_3$) δ 192.1, 174.6, 144.5, 112.5, 45.2, 31.0, 17.6. HRMS ESI (m/z): $[M+H]^+$ calcd. for $C_7H_{10}NO_2$: 140.0712, found: 140.0711.

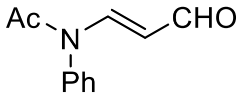

2y

The product **2y** was obtained with flash column chromatography (petroleum ether/ethyl acetate = 3:1) as white solid (53% yield). ^{1}H NMR (400 MHz, $CDCl_3$) δ 9.47 (d, *J* = 8.0 Hz, 1H), 8.49 (d, *J* = 16.0 Hz, 1H), 7.53-7.47 (m, 3H), 7.17 (d, *J* = 8.0 Hz, 2H), 5.03 (dd, *J* = 16.0, 8.0 Hz, 1H), 1.99(s, 3H). ^{13}C NMR (101 MHz, $CDCl_3$) δ 192.1, 169.8, 149.4, 138.1, 130.8, 130.0, 128.2, 114.5, 23.5. HRMS ESI (m/z): $[M+Na]^+$ calcd. for $C_{11}H_{11}O_2Na$: 212.0687, found: 212.0688.

Procedure of Modification of Estrone Derivative 6.

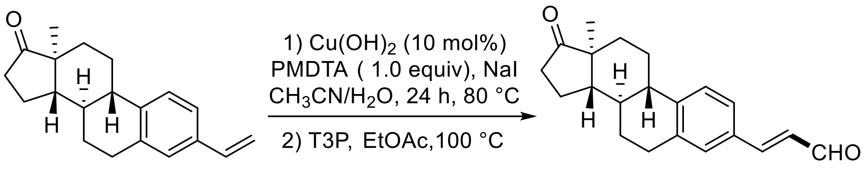

To a 50 mL of Schlenk tube were added estrone derivative **6** (0.2 mmol, 1.0 equiv), $Cu(OH)_2$ (0.02 mmol, 10 mol%) under air, followed by NaI (0.2 mmol, 1.0 equiv). The mixture was then evacuated and back filled with N_2 (3 times). Bromodichoromethane (0.6 mmol, 2.0 equiv), PMDTA (0.2 mmol, 1.0 equiv) and CH_3CN/H_2O (v/v = 1/1) (1 mL) were added subsequently. The Schlenk tube was screw capped and put into a preheated oil bath (80 ˚C). After stirring for 24 hours, the reaction mixture was cooled to room temperature, and then extracted with ethyl acetate for 3 times. After the solvent was removed under rotary evaporation, the residue was resolved in ethyl acetate (5 mL) and T3P (1-Propanephosphonic acid cyclic anhydride 50% ethyl acetate, 0.6 mmol, 3.0 equiv) was added. The mixture was stirred at 100 ˚C overnight, and the reaction was quenched by water (5 mL) and stirred for a few more minutes, extracted with ethyl acetate for 3 times. The solvent was removed under rotary evaporation, and the residue was then purified by flash column chromatography (petroleum ether/ethyl acetate = 5:1) to give **7** as pale yellow solid (50% yield).

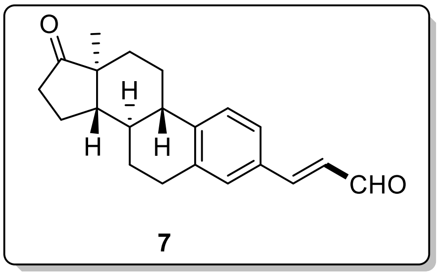

7: ^{1}H NMR (400 MHz, $CDCl_3$) δ 9.67 (d, *J* = 4.0 Hz, 1H), 7.43 (d, *J* = 16.0 Hz, 1H), 7.36 (m, 2H), 7.28 (d, *J* = 16.0 Hz, 1H), 6.68 (dd, *J* = 16.0, 8.0 Hz, 1H), 2.97-2.93 (m,1H), 2.53-2.36 (m, 2H), 2.34-2.31 (m, 1H), 2.20-1.96 (m, 4H), 1.64-1.47 (m, 7H), 0.92 (s, 3H). ^{13}C NMR (101 MHz, $CDCl_3$) δ 194.0, 153.0, 143.8, 137.5, 131.7, 129.3, 128.1, 126.3, 126.1, 50.6, 48.0, 44.8, 38.0, 35.9, 31.6, 29.4, 26.4, 25.7, 21.7, 13.9. HRMS ESI (m/z): $[M+H]^+$

calcd. for $C_{21}H_{25}O_2$: 309.1855. found: 309.1853.

Mechanistic Studies

1. Isolation of the Alcohol Intermediate 2aa

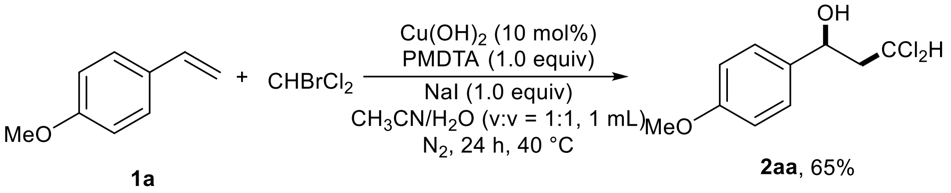

To a 50 mL of Schlenk tube was added $Cu(OH)_2$ (0.02 mmol, 10 mol%) under air, followed by NaI (0.2 mmol, 1 equiv). The mixture was then evacuated and back filled with N_2 (3 times). 4-Methoxystrene **1a** (0.2 mmol, 1 equiv), bromodichoromethane (0.4mmol, 2 equiv), PMDTA (0.2 mmol, 1.0 equiv) and CH_3CN/H_2O (v/v=1/1) (1 mL) were added subsequently. The Schlenk tube was screw capped and put into a preheated oil bath (40 ˚C). After stirring for 24 hrs, the reaction mixture was cooled to room temperature. The reaction mixture was extracted with ethyl acetate for 3 times and the solvent was removed under rotary evaporation. The residue was then resolved in MeOH (5 mL), and $NaBH_4$ (11.4 mg, 0.3 mmol, 1.5 equiv) was added. After 6 more hours' stirring at room temperature, the reaction was quenched with water and extracted with ethyl acetate for 3 times. The solvent was then removed under rotary evaporation, and the residue was purified by flash column chromatography (petroleum ether/ethyl acetate = 6:1) to give **2aa** as pale yellow liquid (65% yield).

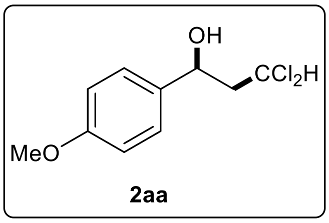

The product **2aa**: ^{1}H NMR (400 MHz, $CDCl_3$) δ 7.27 (d, *J* = 8.0 Hz, 2H), 6.89 (d, *J* = 8.0 Hz, 2H), 5.86 (dd, *J* = 8.0, 4.0 Hz, 1H), 4.87 (dd, *J* = 4.0, 1.2 Hz, 1H), 3.80 (s, 3H),

2.68-2.61 (m,1H), 2.46-2.39 (m, 1H), 2.16 (b, 1H). ^{13}C NMR (101 MHz, $CDCl_3$) δ 159.6, 134.9, 127.2, 114.3, 71.0, 71.0, 55.5, 52.3. HRMS ESI (m/z): $[M+H]^+$ calcd. for $C_{10}H_{13}Cl_2O_2$: 235.0293, found: 235.0278.

2. Isolation of Compound 2ab

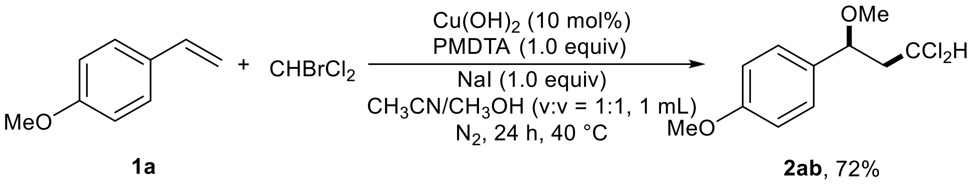

To a 50 mL of Schlenk tube was added $Cu(OH)_2$ (0.02 mmol, 10 mol%) under air, followed by NaI (0.2 mmol, 1 equiv). The mixture was then evacuated and back filled with N_2 (3 times). 4-Methoxystrene **1a** (0.2 mmol, 1 equiv), bromodichoromethane (0.6 mmol, 3.0 equiv), PMDTA (0.2 mmol, 1.0 equiv) and CH_3CN/CH_3OH (v/v=1/1) (1 mL) were added subsequently. The Schlenk tube was screw capped and put into a preheated oil bath (40 ˚C). After stirring for 24 hours, the reaction mixture was cooled to room temperature. The reaction mixture was extracted with ethyl acetate for 3 times and the solvent was removed under rotary evaporation. The residue was then purified by flash column chromatography (petroleum ether/ethyl acetate = 6:1) to give **2ab** as pale yellow liquid (72% yield).

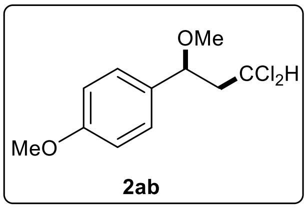

2ab: ^{1}H NMR (400 MHz, $CDCl_3$) δ 7.25-7.21 (m, 2H), 6.93-6.90 (m, 2H), 5.87 (dd, *J* = 9.2, 4.4 Hz, 1H), 4.31 (dd, *J* = 9.6, 4.0 Hz, 1H), 3.82 (s, 3H), 3.18 (s, 3H) 2.68-2.63 (m,1H), 2.40-2.34 (m, 1H). ^{13}C NMR (101 MHz, $CDCl_3$) δ 159.7, 132.0, 128.0, 114.2, 80.0, 70.9, 56.6, 55.4, 52.0. HRMS ESI (m/z): $[M+H]^+$ calcd. for $C_{11}H_{15}Cl_2O_2$: 249.0449, found: 249.0455.

3. Radical Trapping Experiment with Radical Clock.

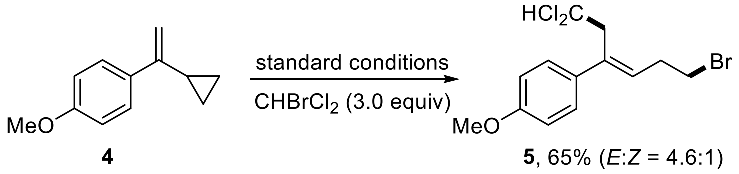

To a 50 mL of Schlenk tube was added $Cu(OH)_2$ (0.02 mmol, 10 mol%) under air, followed by NaI (0.2 mmol, 1 equiv). The mixture was then evacuated and back filled with N_2 (3 times). 1-(1-cyclopropylvinyl)-4-methoxybenzene **4** (0.2 mmol, 1 equiv), bromodichoromethane (0.6 mmol, 3.0 equiv), PMDTA (0.2 mmol, 1.0 equiv) and CH_3CN/H_2O (v/v=1/1) (1 mL) were added subsequently. The Schlenk tube was screw capped and put into a preheated oil bath (40 ˚C). After stirring for 24 hours, the reaction mixture was cooled to room temperature. The reaction mixture was extracted with ethyl acetate for 3 times and the solvent was removed under rotary evaporation. The residue was then purified by preparative TLC (petroleum ether only) to give **5** as colorless liquid (65% yield).

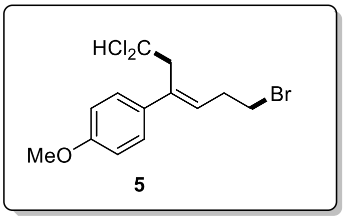

5 (*E*): ^1H NMR (400 MHz, $CDCl_3$) δ 7.25 (d, J = 8.0 Hz, 2H), 6.90 (d, J = 8.0 Hz, 2H), 5.78-5.69 (m, 1H), 5.55 (t, J = 8.0 Hz, 1H), 3.82 (s, 3H), 3.48 (t, J = 8.0 Hz, 1H), 3.40 (t, J = 8.0 Hz, 2H), 3.23 (t, J = 8.0 Hz, 1H), 2.90-2.83 (m, 2H).^{13}C NMR (101 MHz, $CDCl_3$) δ 159.4, 136.1, 130.8, 129.1, 128.8, 114.2, 71.4, 55.4, 44.4, 32.8, 32.2. **5** (*Z*): ^1H NMR (400 MHz, $CDCl_3$) δ 7.08 (d, J = 8.0 Hz, 2H), 6.91 (d, J = 8.0 Hz, 2H), 5.66-5.60 (m, 1H), 5.43 (t, J = 8.0 Hz, 1H), 3.82 (s, 3H), 3.34 (t, J = 8.0 Hz, 1H), 3.20 (t, J = 8.0 Hz, 2H). ^{13}C NMR (101 MHz, $CDCl_3$) δ 159.2, 136.5, 132.9, 130.1, 130.0, 129.2, 71.3, 55.4, 53.6, 32.8, 32.3. EI (m/z): $[M+H]^+$ calcd. for $C_{13}H_{16}BrCl_2O$:

336.9762, found: 336.9736.

4. Radical Trapping Experiment with BHT.

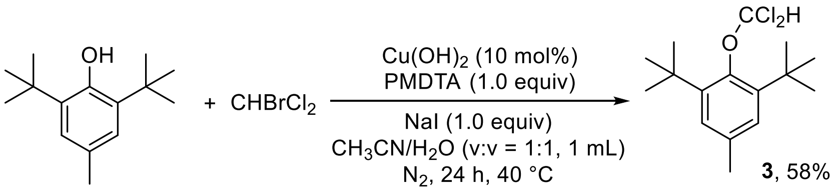

To a 50 mL of Schlenk tube were added $Cu(OH)_2$ (0.02 mmol, 10 mol%) and 2,6-di-tert-butyl-4-methylphenol (BHT) (0.2 mmol, 1.0 equiv) under air, followed by NaI (0.2 mmol, 1.0 equiv). The mixture was then evacuated and back filled with N_2 (3 times). Bromodichoromethane (0.6 mmol, 3.0 equiv), PMDTA (0.2 mmol, 1.0 equiv) and CH_3CN/H_2O (v/v=1/1) (1 mL) were added subsequently. The Schlenk tube was screw capped and put into a preheated oil bath (40 °C). After stirring for 24 hours, the reaction mixture was cooled to room temperature. The reaction mixture was extracted with ethyl acetate for 3 times and the solvent was removed under rotary evaporation. The residue was then purified by preparative TLC (petroleum ether only) to give **3** as colorless solid (58% yield).

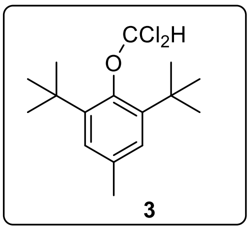

3: ^{1}H NMR (400 MHz, $CDCl_3$) δ 6.54 (s, 2H), 5.63 (s, 1H), 1.42 (s, 3H), 1.24 (s, 18H). ^{13}C NMR (101 MHz, $CDCl_3$) δ 185.8, 149.3, 139.9, 78.9, 46.5, 35.2, 29.5. EI (m/z): $[M+Na]^+$ calcd. for $C_{16}H_{24}Cl_2ONa$: 325.1102, found: 325.1101.

4. Radical Trapping Experiment with TEMPO.

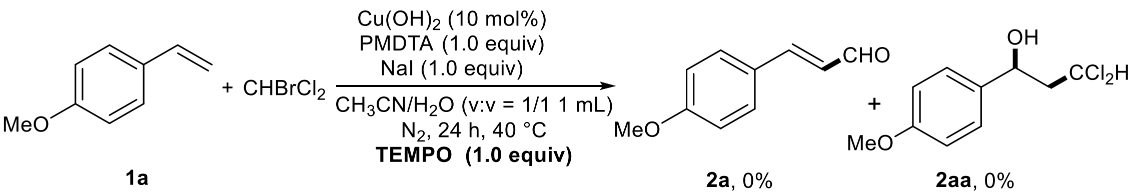

To a 50 mL of Schlenk tube were added $Cu(OH)_2$ (0.02 mmol, 10 mol%), 2,2,6,6-tetramethyl-1-piperidinyloxy (TEMPO) (0.2 mmol, 1.0 equiv) under air, followed by NaI (0.2 mmol, 1.0 equiv). The mixture was then evacuated and back filled with N_2 (3 times). 4-Methoxystrene **1a** (0.2 mmol, 1.0 equiv),

bromodichoromethane (0.6 mmol, 3.0 equiv), PMDTA (0.2 mmol, 1.0 equiv) and CH_3CN/H_2O (v/v = 1/1, 1 mL) were added subsequently. The Schlenk tube was screw capped and put into a preheated oil bath (40 ˚C). After stirring for 24 hours, the reaction mixture was cooled to room temperature. The reaction mixture was extracted with ethyl acetate for 3 times and the solvent was removed under rotary evaporation. The residue was detected by GC-MS directly, which indicated that TEMPO quenched the reaction completely.

Dichoromethylation of 2-phenylpropene

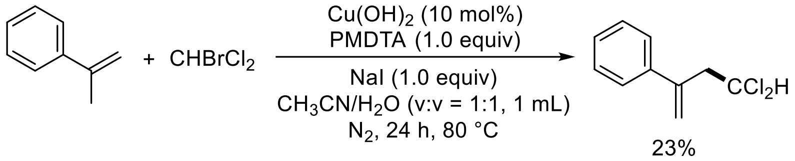

To a 50 mL of Schlenk tube was added $Cu(OH)_2$ (0.02 mmol, 10 mol%) under air, followed by NaI (0.2 mmol, 1.0 equiv). The mixture was then evacuated and back filled with N_2 (3 times). 2-phenylpropene (0.2 mmol, 1.0 equiv), bromodichoromethane (0.6 mmol, 3.0 equiv), PMDTA (0.2 mmol, 1.0 equiv) and CH_3CN/H_2O (v/v = 1/1, 1 mL) were added subsequently. The Schlenk tube was screw capped and put into a preheated oil bath (40 ˚C). After stirring for 24 hours, the reaction mixture was cooled to room temperature, and then extracted with ethyl acetate for 3 times. The solvent was removed under rotary evaporation, and the residue was then purified by preparative TLC (petroleum ether) to give 4,4-dichloro-2-phenyl-1-butene as a yellow liquid (23%, yield).[8]

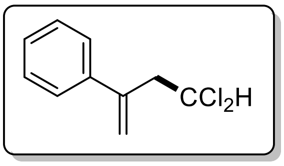

4,4-dichloro-2-phenyl-1-butene:[8] ^{1}H NMR (400 MHz, $CDCl_3$) δ 7.23 (m, 5H), 5.65 (t, *J* = 8.0 Hz, 1H), 5.45 (d, *J* = 1.2 Hz 1H), 5.27 (dd, *J* = 2.0, 1.2 Hz, 1H), 3.40 (dd, *J* = 6.8, 0.8 Hz, 2H). ^{13}C NMR (101 MHz, $CDCl_3$) δ 142.8, 139.3, 128.8, 128.3, 126.4, 117.8, 71.3, 50.5.

I/Br exchange of the starting material

The ^{1}H NMR analysis of the mixture from the crude reaction system with CD_3Cl as the solvent gave a new signal at 7.00 ppm, which could also be detected by mixing $BrCCl_2H$ and NaI in CD_3Cl accordingly. Further GC-MS analysis indicated an in situ-generated $ICCl_2H$.[9]

References:

(1) D. S. Seferos, D. A. Banach, N. A. Alcantar, J. A. Israelachvili and G. C. Bazan, *J. Org. Chem*., 2004, **69**, 1110.
(2) J. Dörfler, T. Preuß, A. Schischko, M. Schmidtmann and S. Doye, *Angew. Chem., Int. ed.*, 2014, **53**, 7918.
(3) C. A. Faler and M. M. Joulilié, *Org. Lett*., 2007, **9**, 1987.
(4) J. B. Feltenberger, R. Hayashi, Y. Tang, E. S. C. Babiash and R. P. Hsung, *Org. Lett*., 2009, **11**, 3666.
(5) C. Chatalova-Sazepin, Q. Wang, G. M. Sammis and J. Zhu, *Angew. Chem., Int. ed.* 2015, **54**, 5443.
(6) L. Crespin, L. Biancalana, T. Morack, D. C. Balkemore and S. V. Ley, *Org. Lett*., 2017, **19**, 1084.
(7) G. Battistuzzi, S. Cacchi and G. Fabrizi, *Org. Lett.*, 2003, **5**, 777.
(8) L. K. Sydnes. *Acta Chemica Scandinavica*, 1990, **44**, 603.
(9) (a) A.-Y. Luo, Y. Bao, X. Cheng and X.-S. Wang, *Synthesis*, 2017, **17**, 3962; (b) D. Sülzle, *Tetrahedron Lett.*, 1987, **28**, 2769.

NMR Spectra of new compounds (^{1}H NMR, ^{13}C NMR)

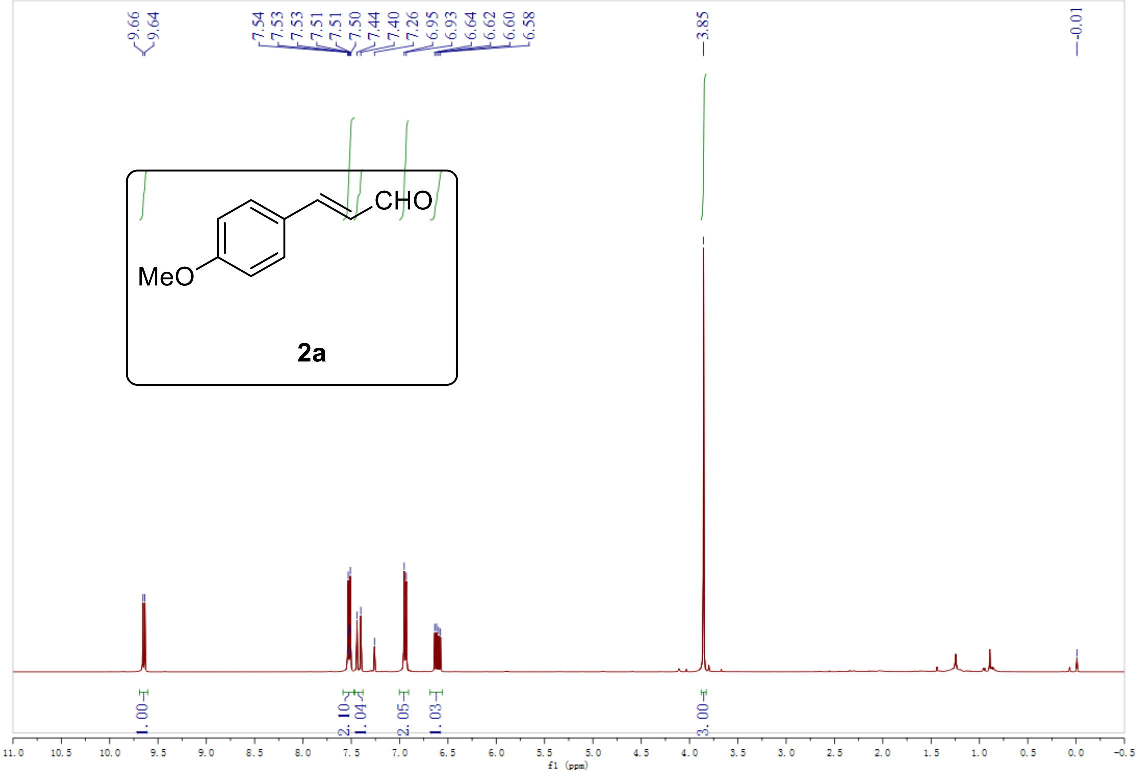

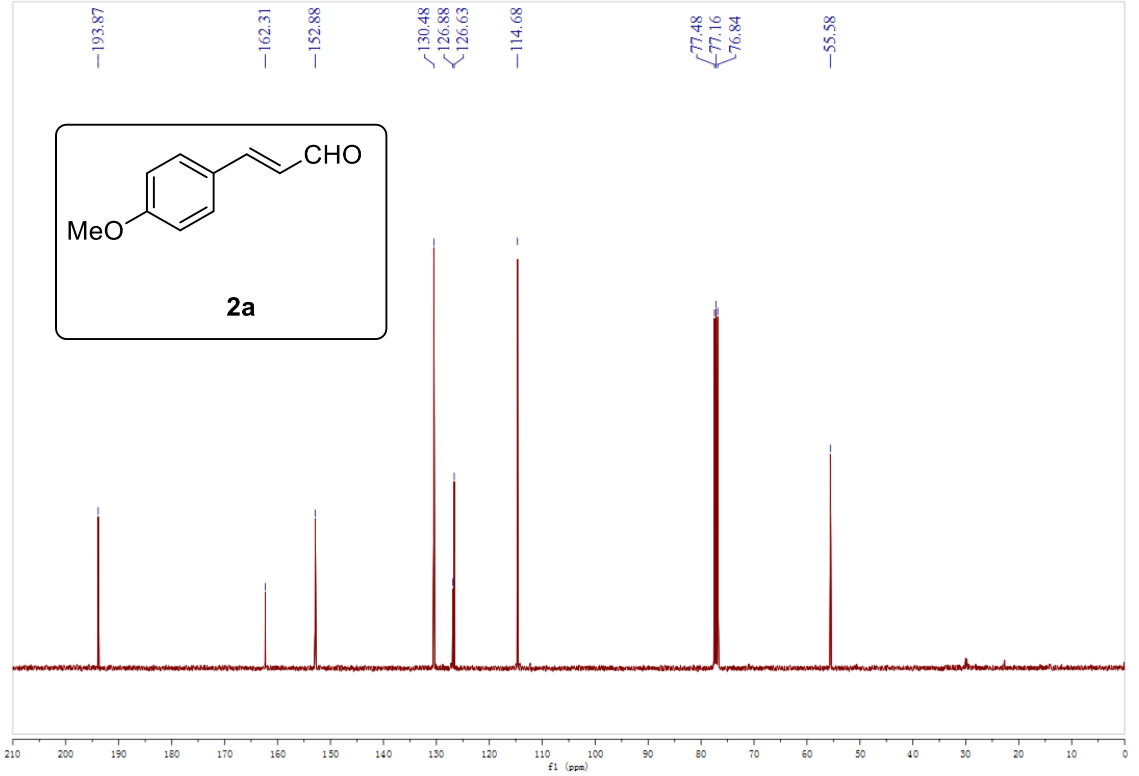

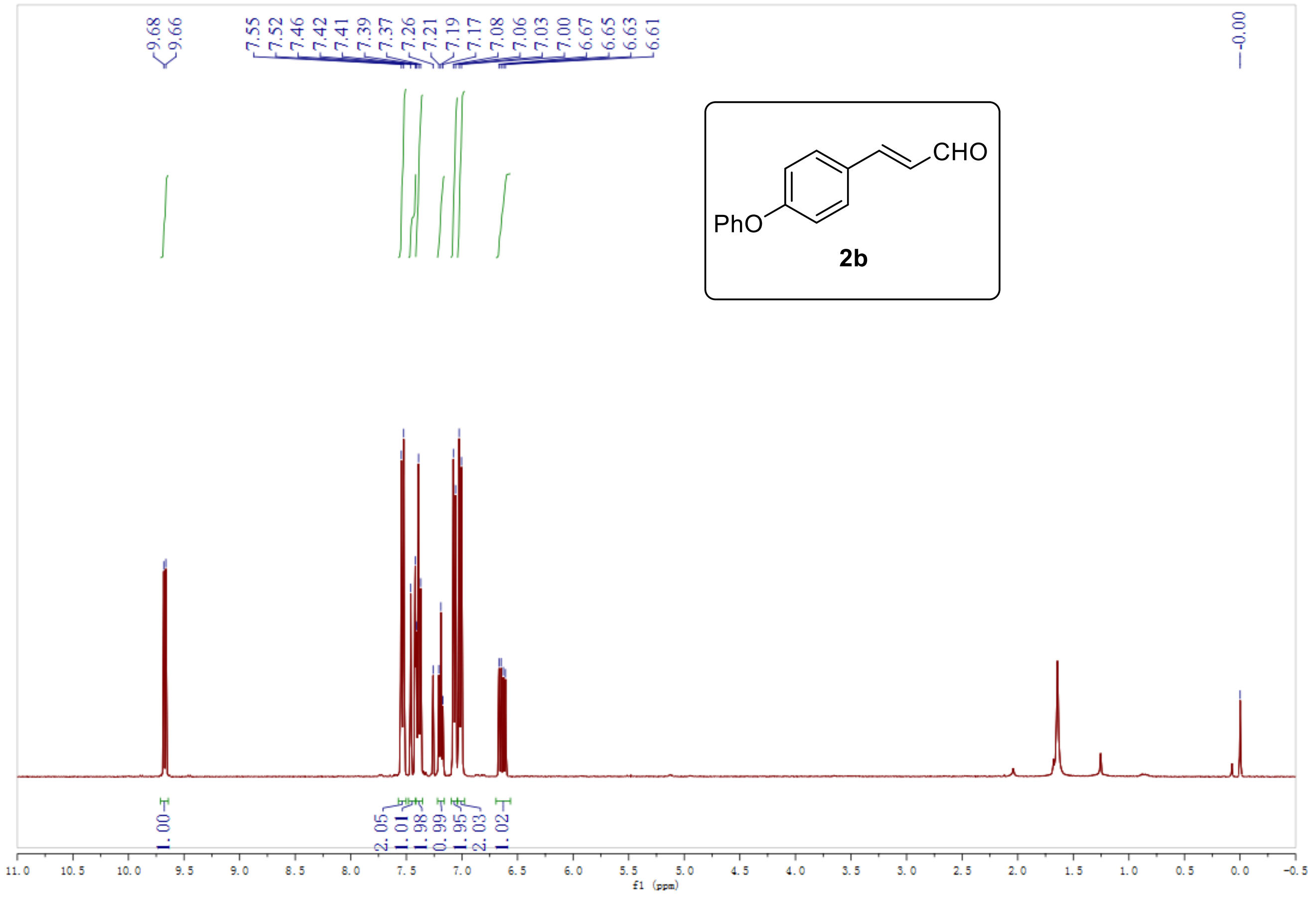
9.68
9.66
7.55
7.52
7.46
7.42
7.41
7.39
7.37
7.26
7.21
7.19
7.17
7.08
7.06
7.03
7.00
6.67
6.65
6.63
6.61
-0.00
CHO
PhO
2b
1.00
2.05
1.01
1.98
0.99
1.95
2.03
1.02
f1 (ppm)

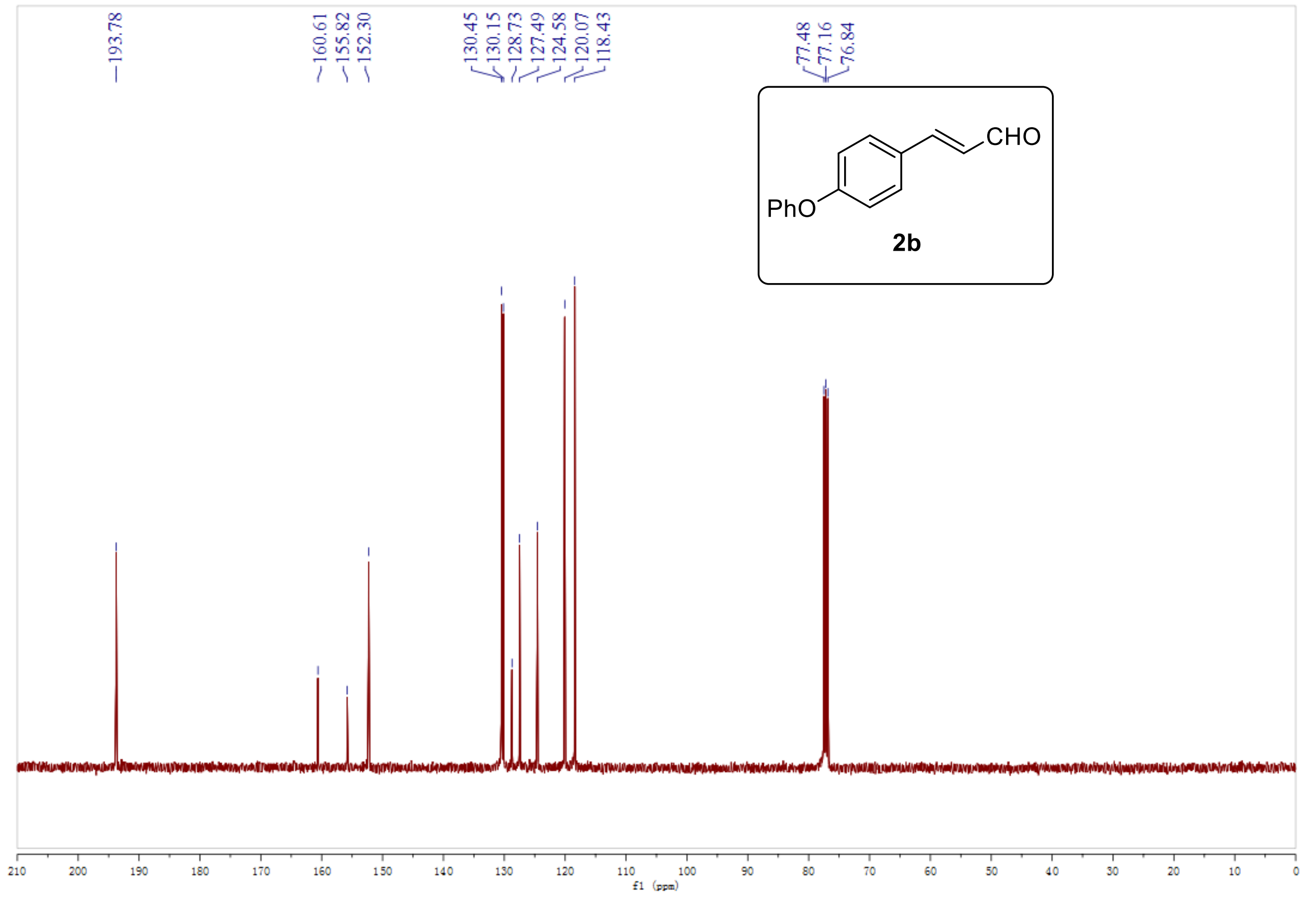
193.78
160.61
155.82
152.30
130.45
130.15
128.73
127.49
124.58
120.07
118.43
77.48
77.16
76.84
CHO
PhO
2b
f1 (ppm)

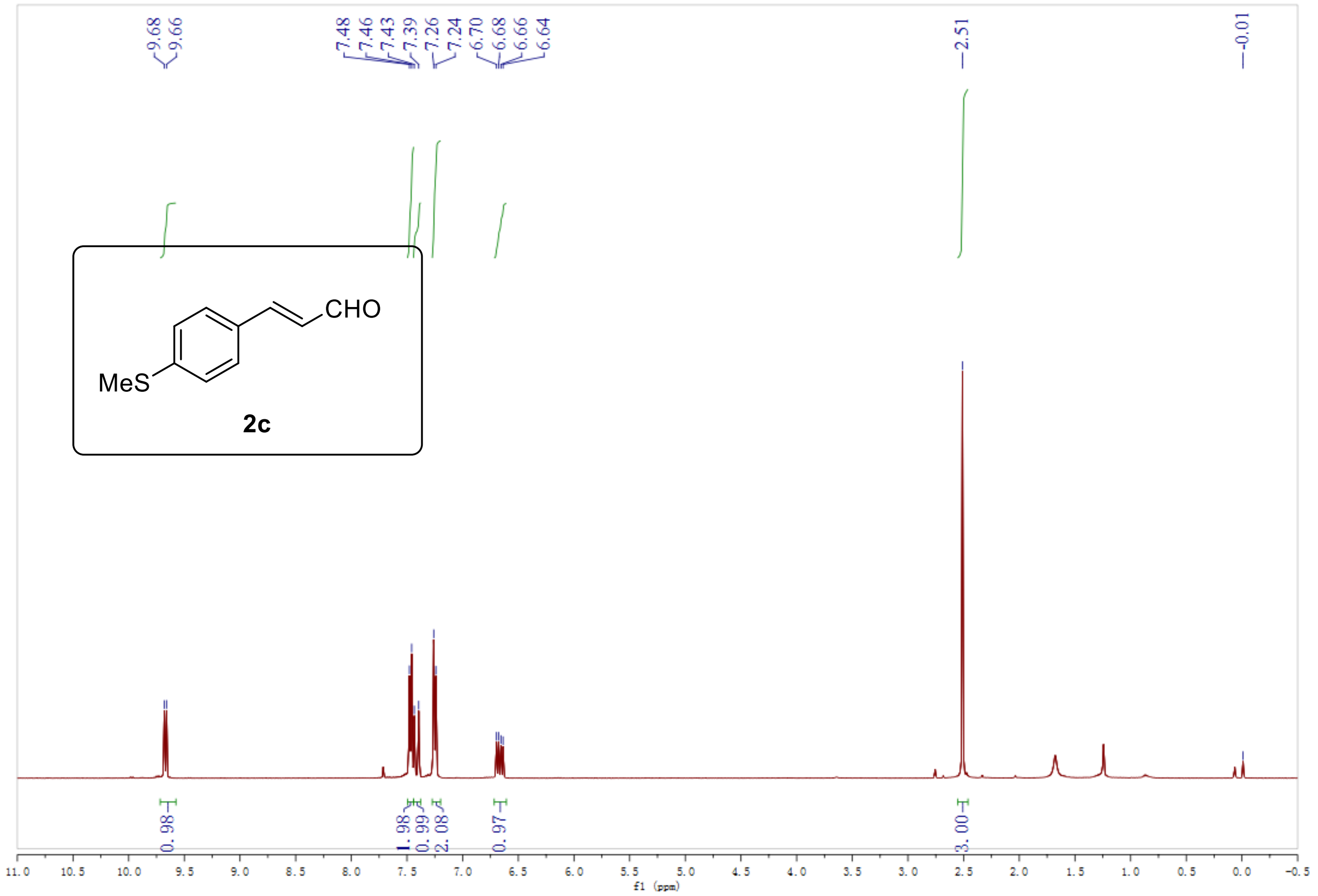
9.68
9.66
7.48
7.46
7.43
7.39
7.26
7.24
6.70
6.68
6.66
6.64
2.51
-0.01
CHO
MeS
2c
0.98
1.98
0.99
2.08
0.97
3.00
f1 (ppm)

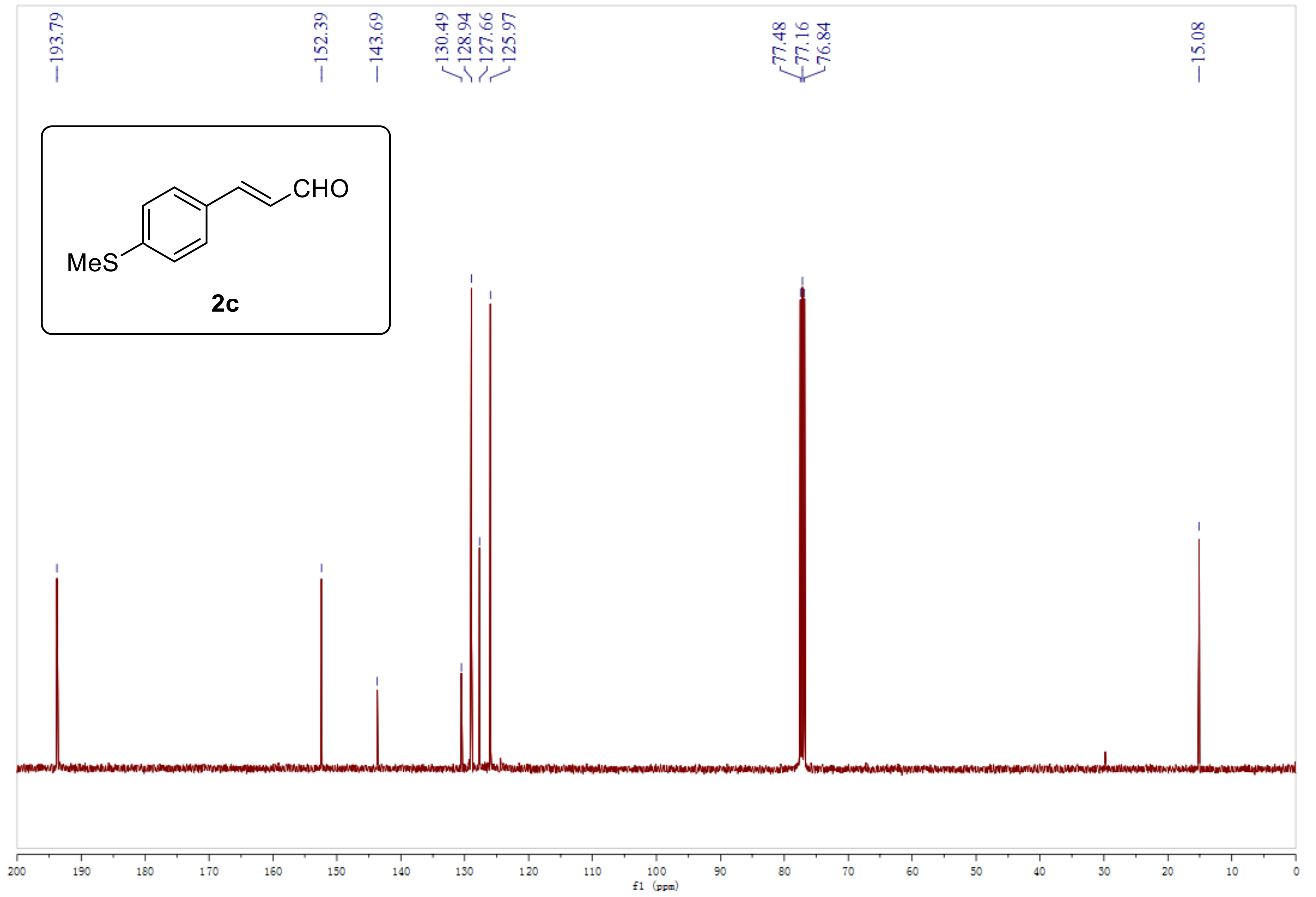
193.79
152.39
143.69
130.49
128.94
127.66
125.97
77.48
77.16
76.84
15.08
CHO
MeS
2c
f1 (ppm)

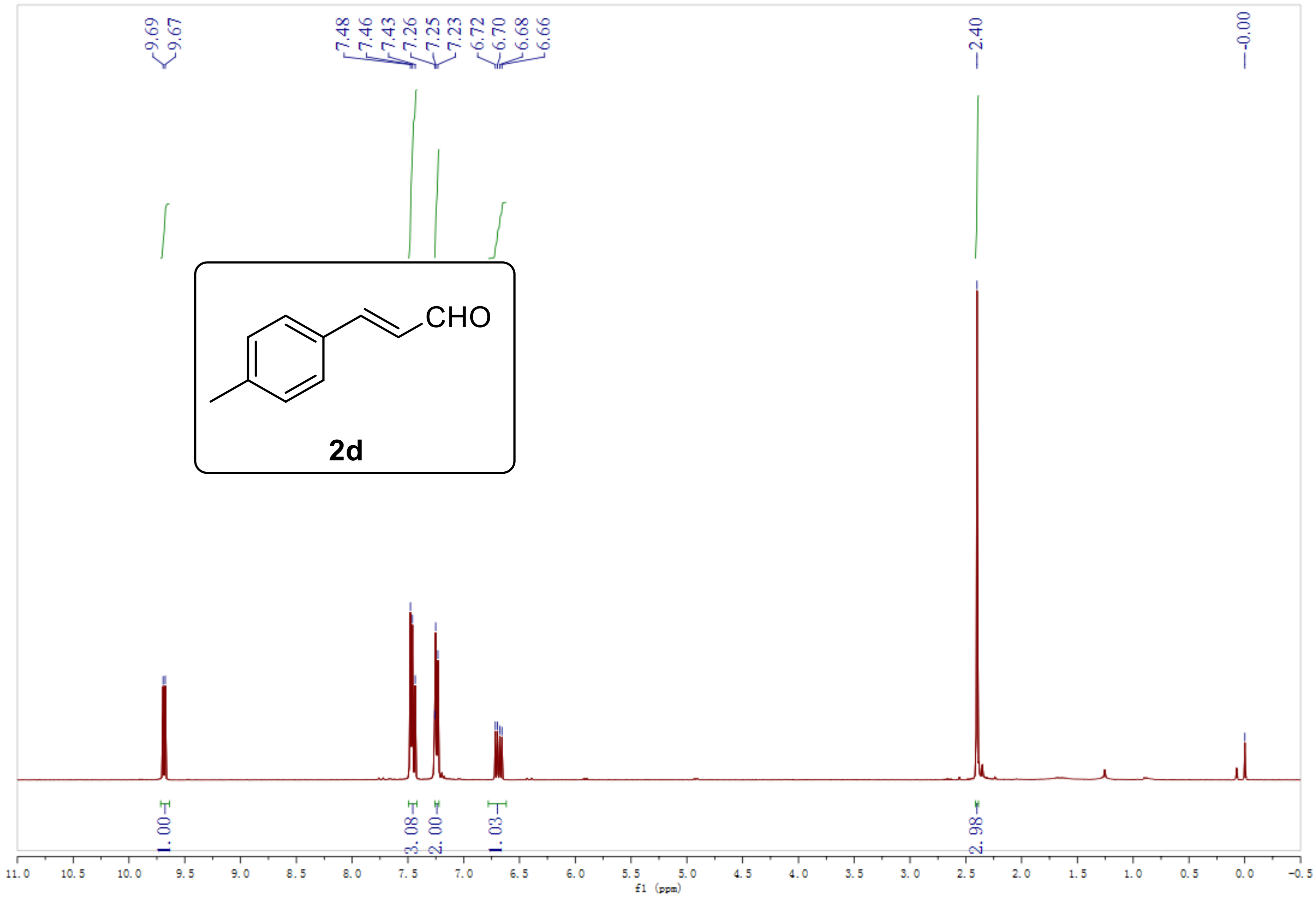

9.69
9.67
7.48
7.46
7.43
7.26
7.25
7.23
6.72
6.70
6.68
6.66
2.40
-0.00
CHO
2d
1.00
3.08
2.00
1.03
2.98
11.0 10.5 10.0 9.5 9.0 8.5 8.0 7.5 7.0 6.5 6.0 5.5 5.0 4.5 4.0 3.5 3.0 2.5 2.0 1.5 1.0 0.5 0.0 -0.5
f1 (ppm)

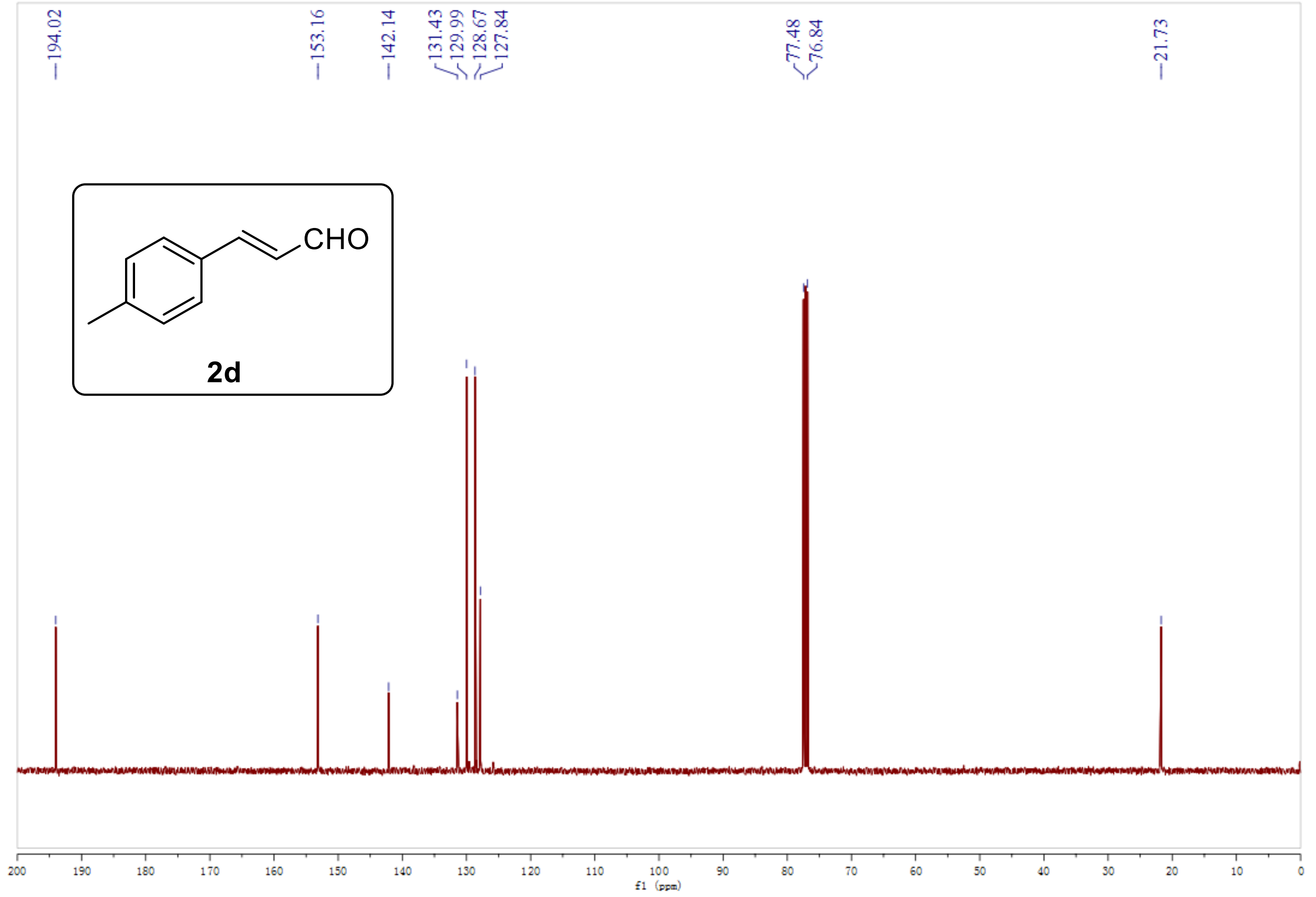

194.02
153.16
142.14
131.43
129.99
128.67
127.84
77.48
76.84
21.73
CHO
2d
200 190 180 170 160 150 140 130 120 110 100 90 80 70 60 50 40 30 20 10 0
f1 (ppm)

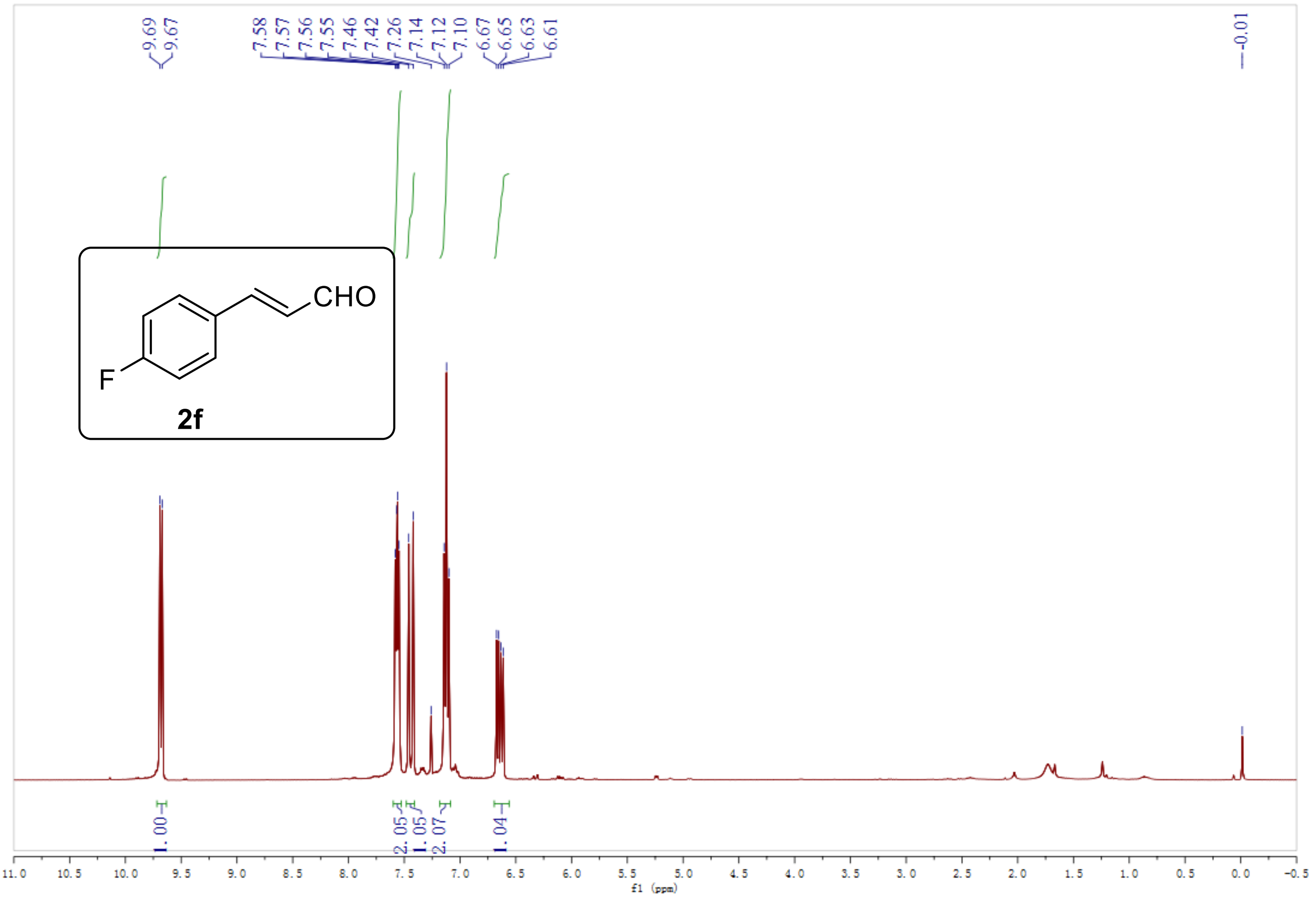
9.69
9.67
7.58
7.57
7.56
7.55
7.46
7.42
7.26
7.14
7.12
7.10
6.67
6.65
6.63
6.61
-0.01
CHO
F
2f
1.00
2.05
1.05
2.07
1.04
f1 (ppm)

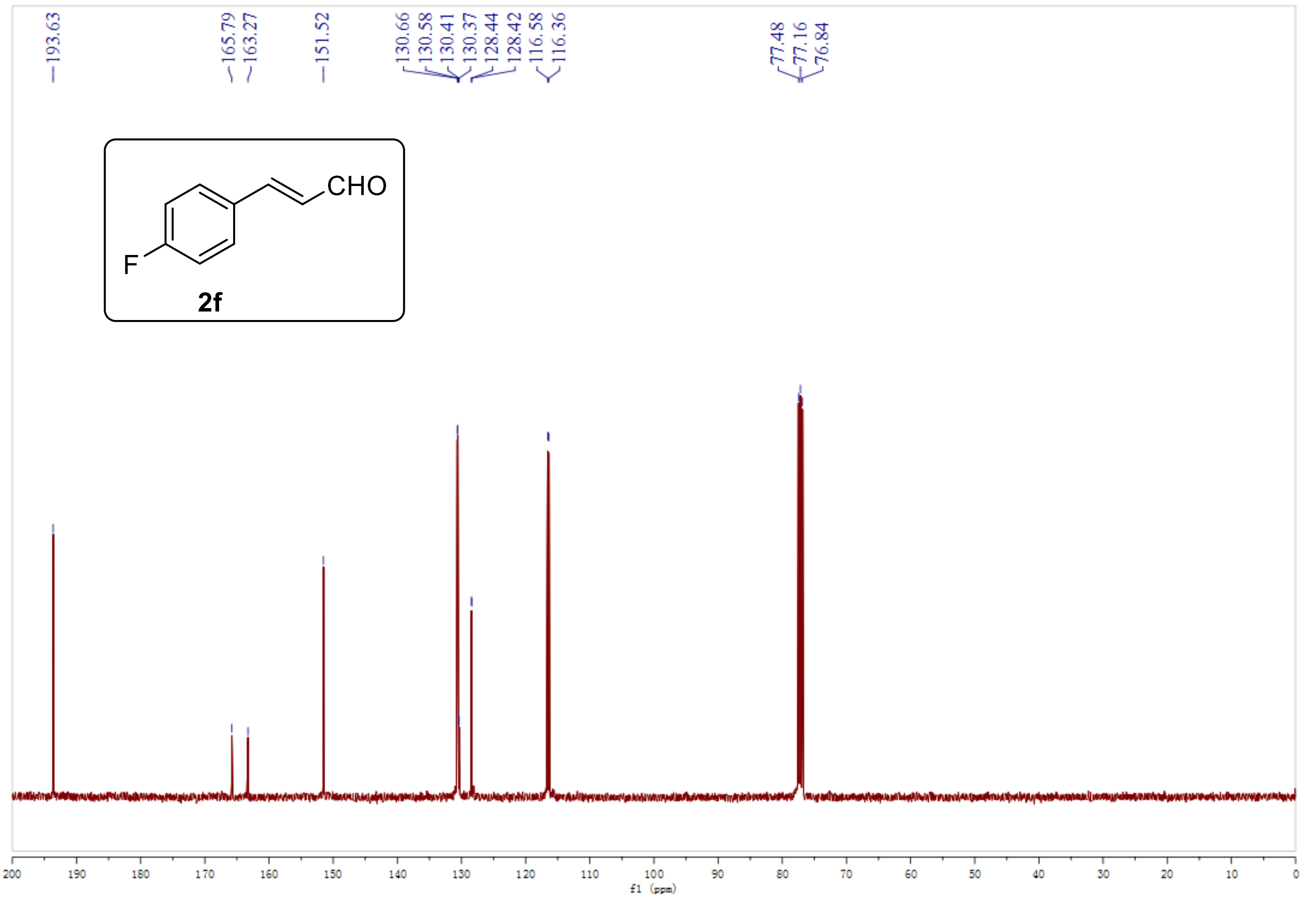
193.63
165.79
163.27
151.52
130.66
130.58
130.41
130.37
128.44
128.42
116.58
116.36
77.48
77.16
76.84
CHO
F
2f
f1 (ppm)

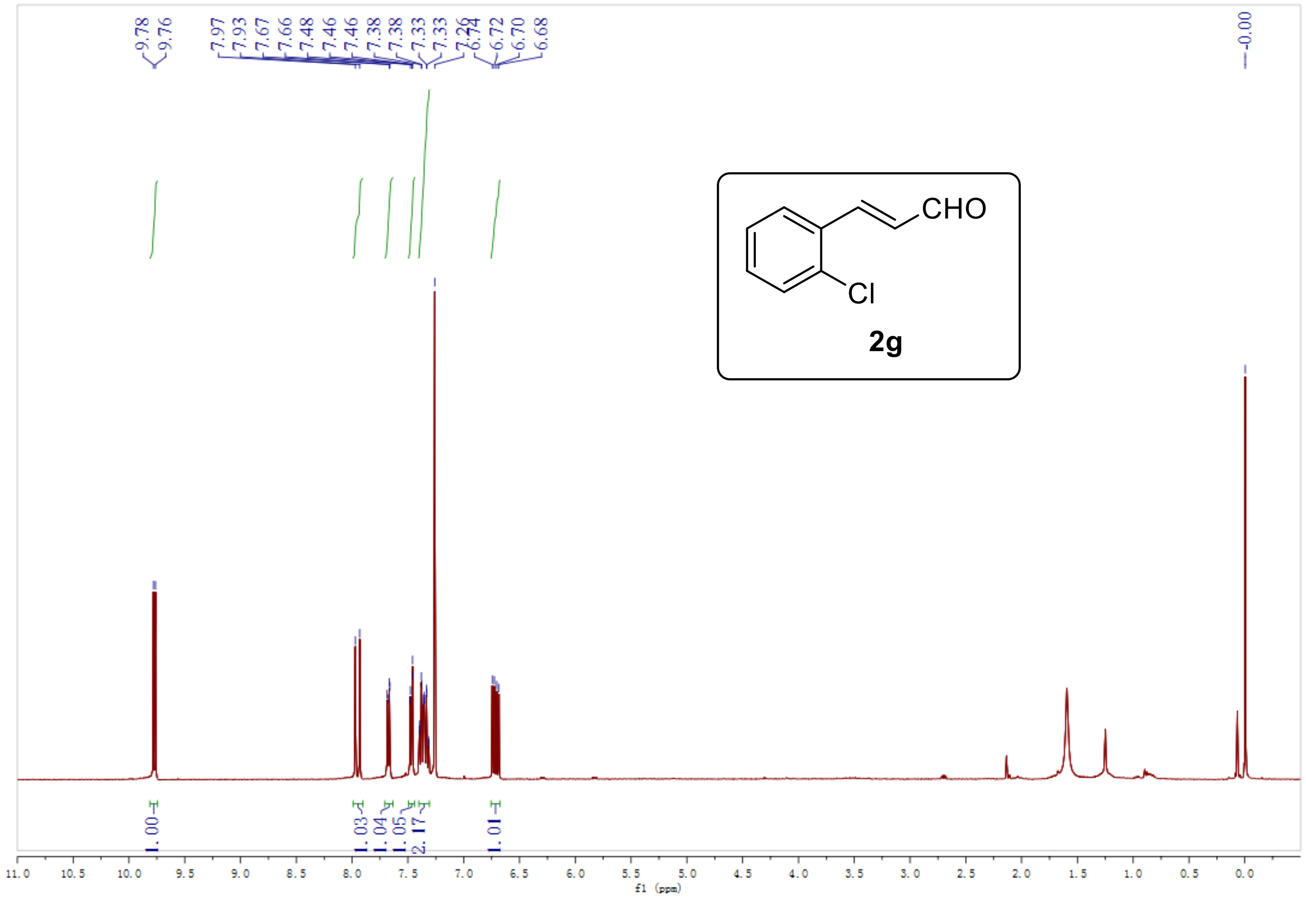
9.78
9.76
7.97
7.93
7.67
7.66
7.48
7.46
7.46
7.38
7.38
7.33
7.33
7.26
6.74
6.72
6.70
6.68
0.00
CHO
Cl
2g
1.00
1.03
1.04
1.05
2.17
1.01
f1 (ppm)

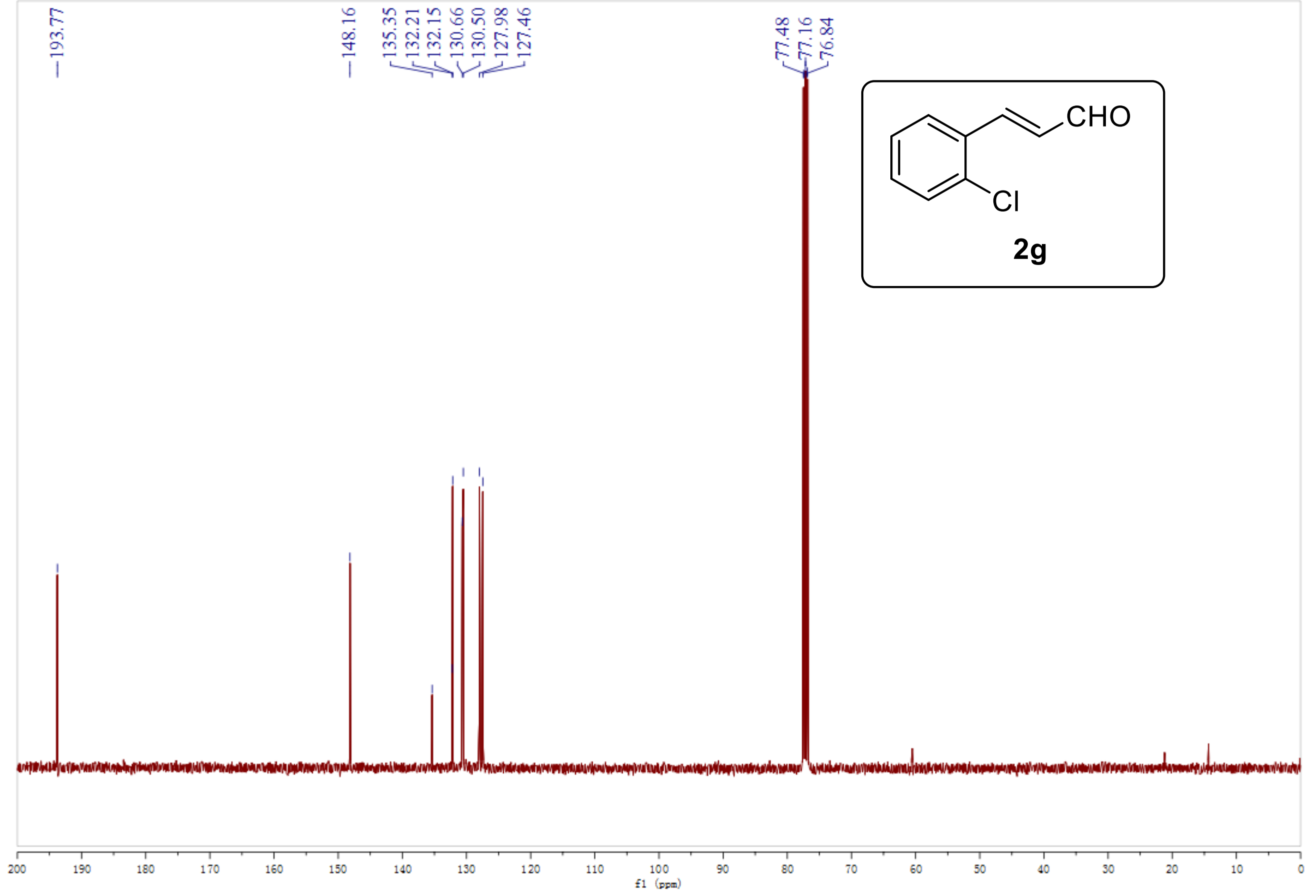
193.77
148.16
135.35
132.21
132.15
130.66
130.50
127.98
127.46
77.48
77.16
76.84
CHO
Cl
2g
f1 (ppm)

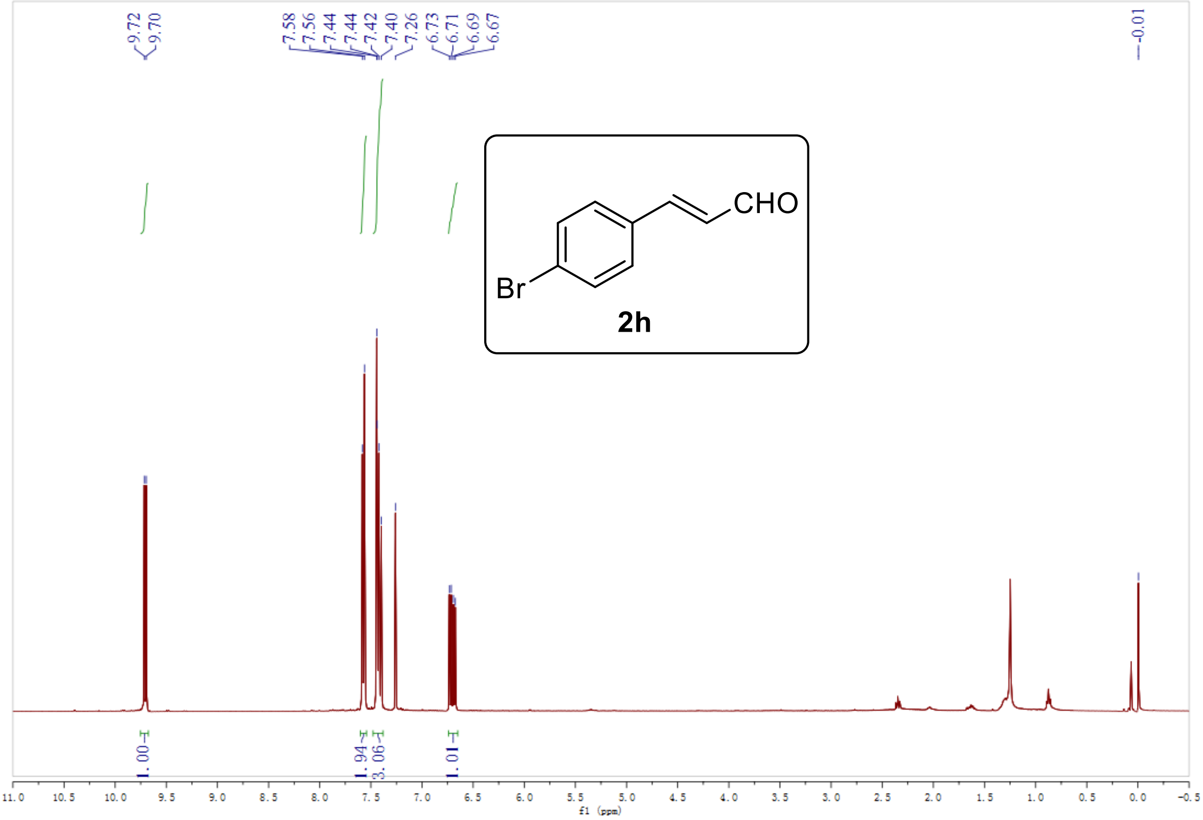
9.72
9.70
7.58
7.56
7.44
7.44
7.42
7.40
7.26
6.73
6.71
6.69
6.67
-0.01
CHO
Br
2h
1.00
1.94
3.06
1.01
f1 (ppm)

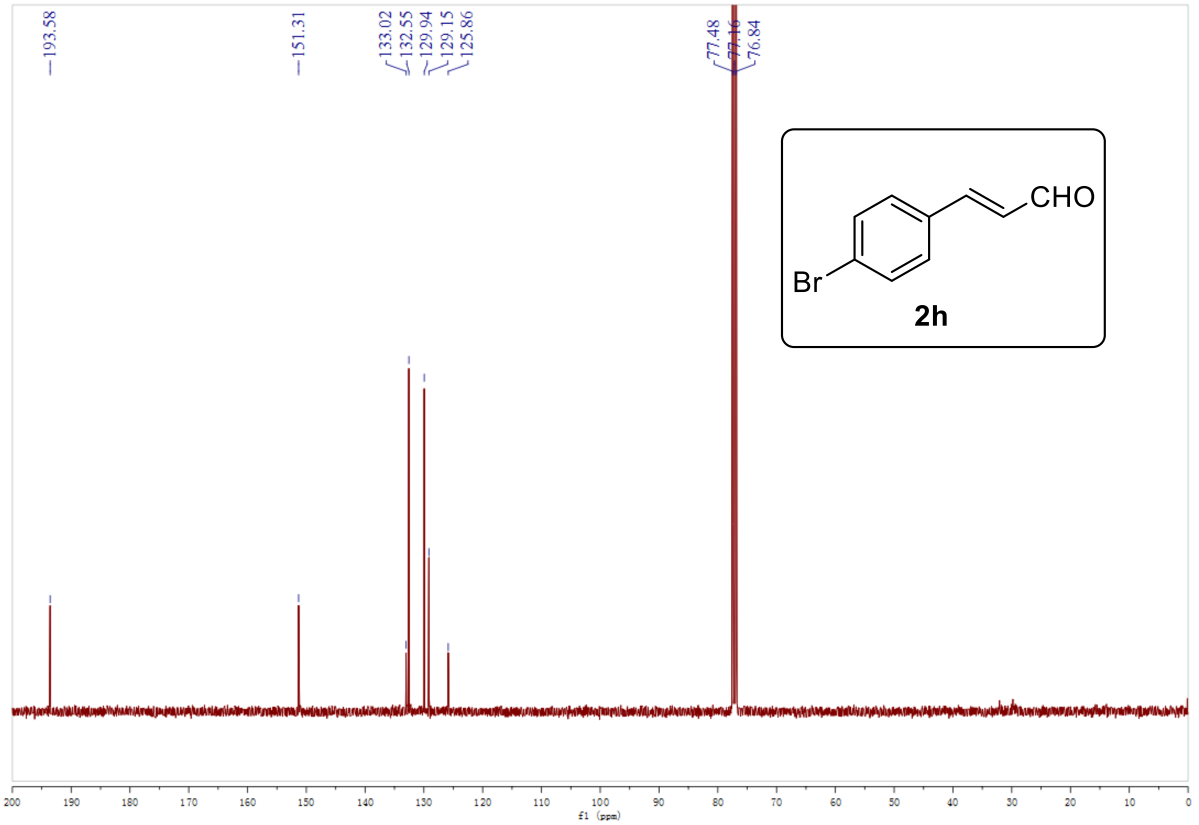
193.58
151.31
133.02
132.55
129.94
129.15
125.86
77.48
77.16
76.84
CHO
Br
2h
f1 (ppm)

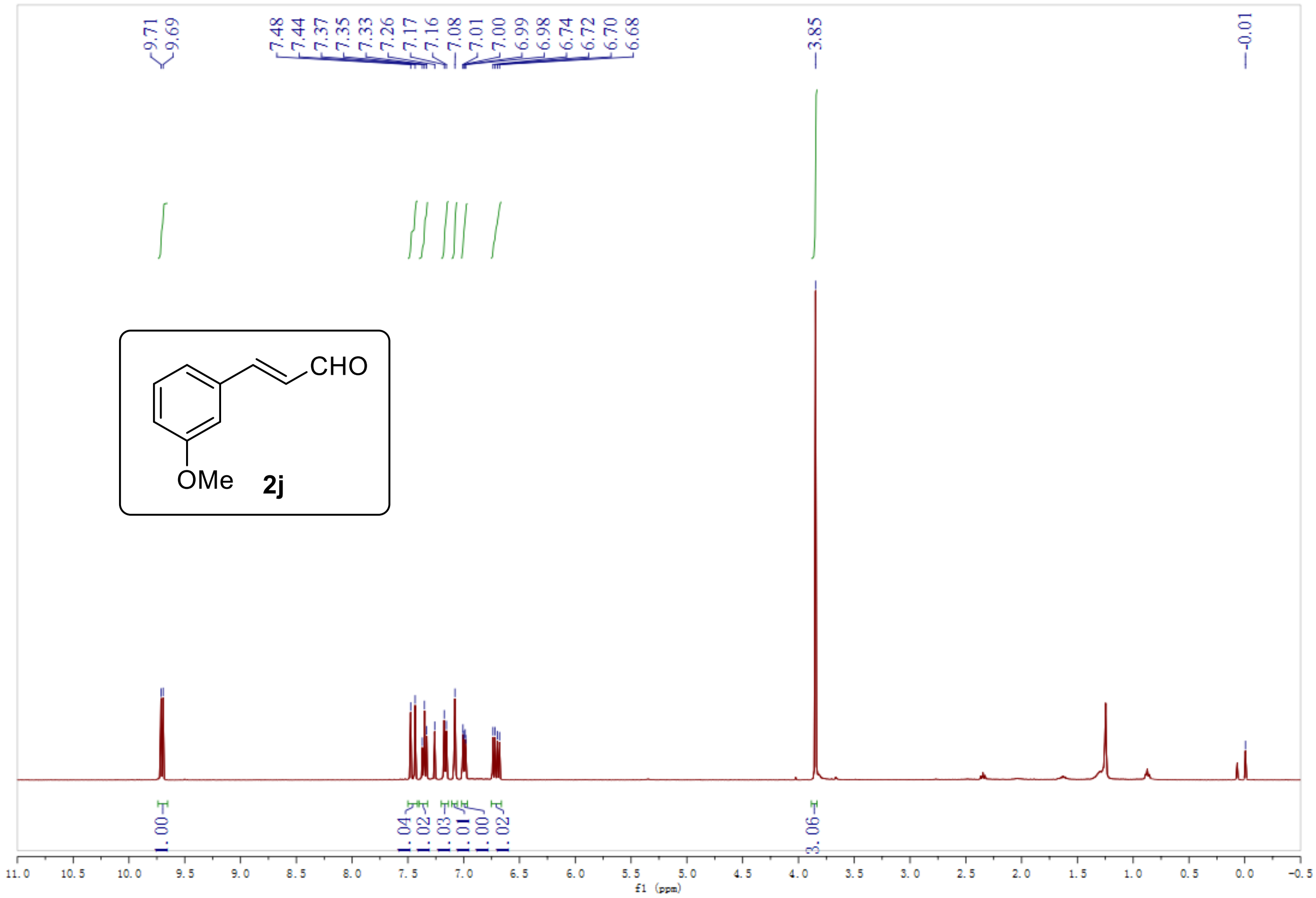

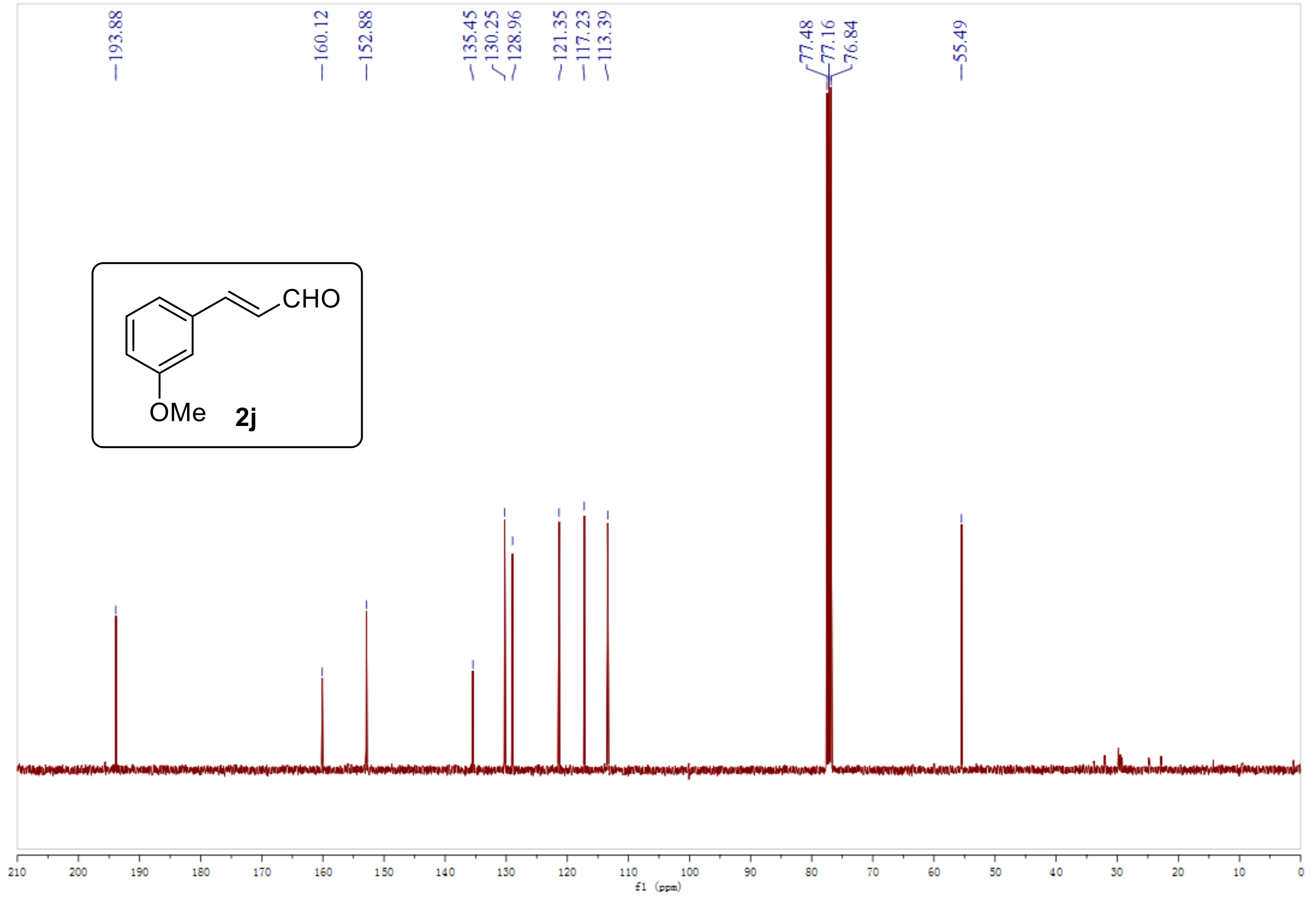

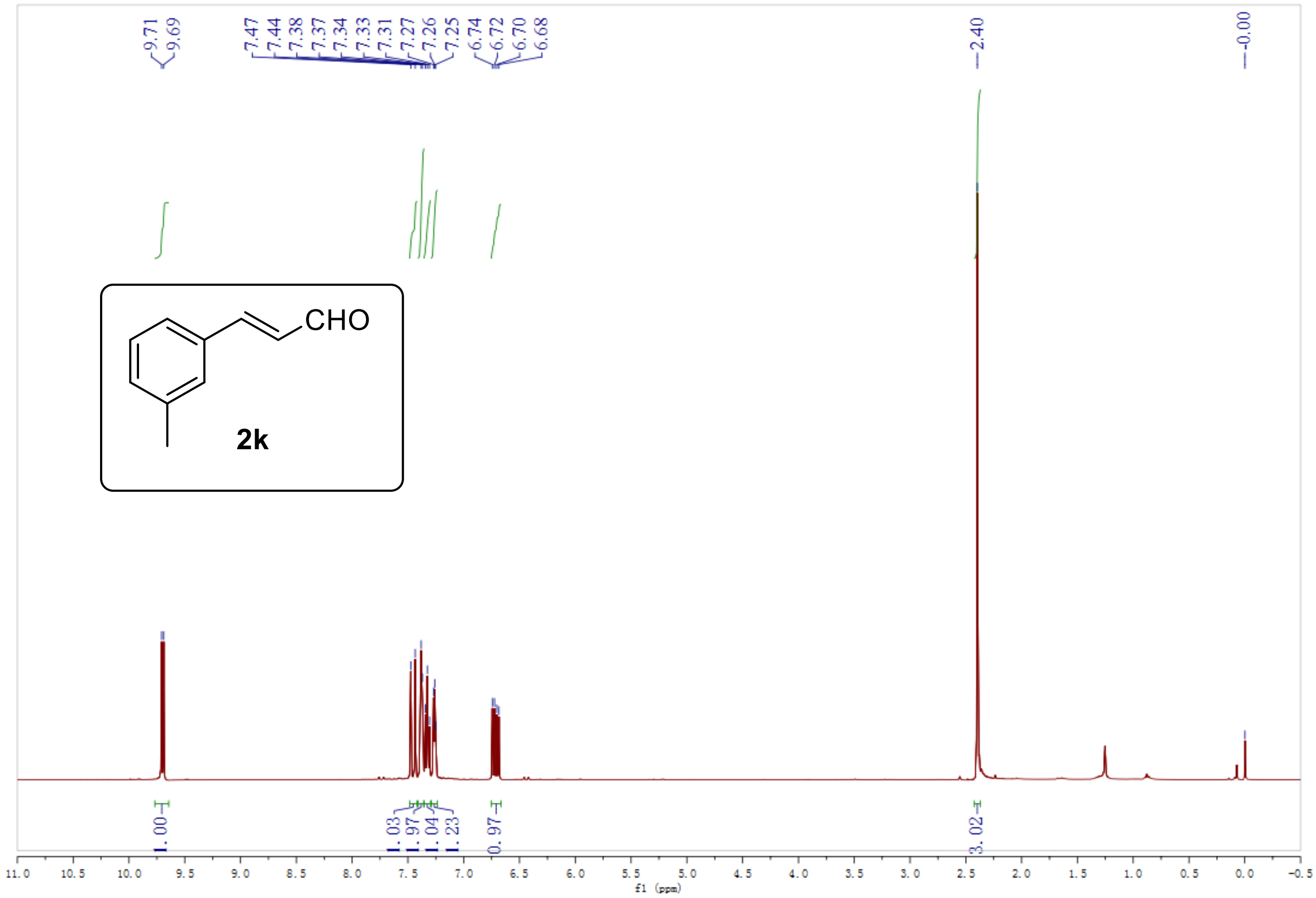
9.71
9.69
7.47
7.44
7.38
7.37
7.34
7.33
7.31
7.27
7.26
7.25
6.74
6.72
6.70
6.68
2.40
0.00
CHO
2k
1.00
1.03
1.97
1.04
1.23
0.97
3.02
f1 (ppm)

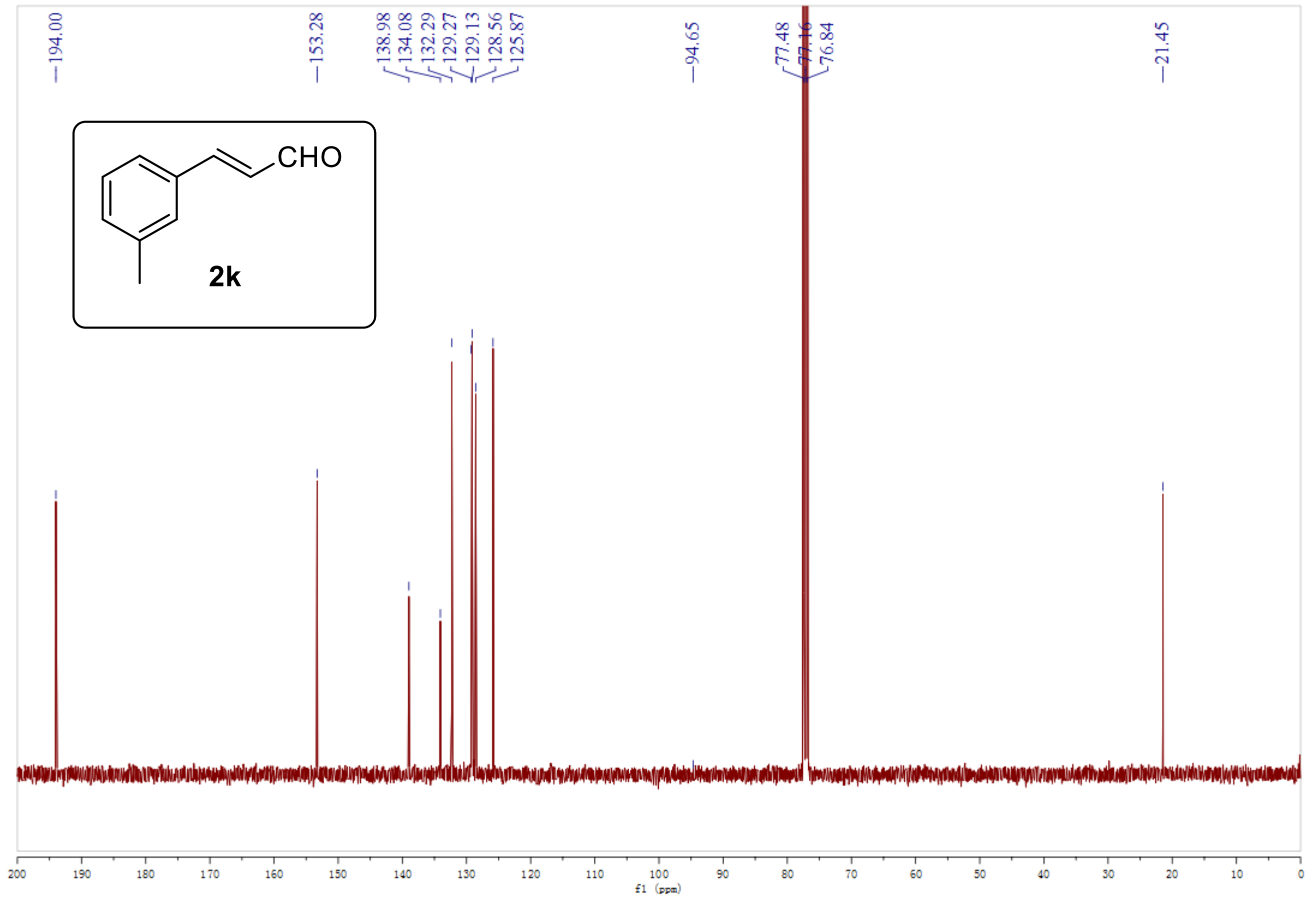
194.00
153.28
138.98
134.08
132.29
129.27
129.13
128.56
125.87
94.65
77.48
77.16
76.84
21.45
CHO
2k
f1 (ppm)

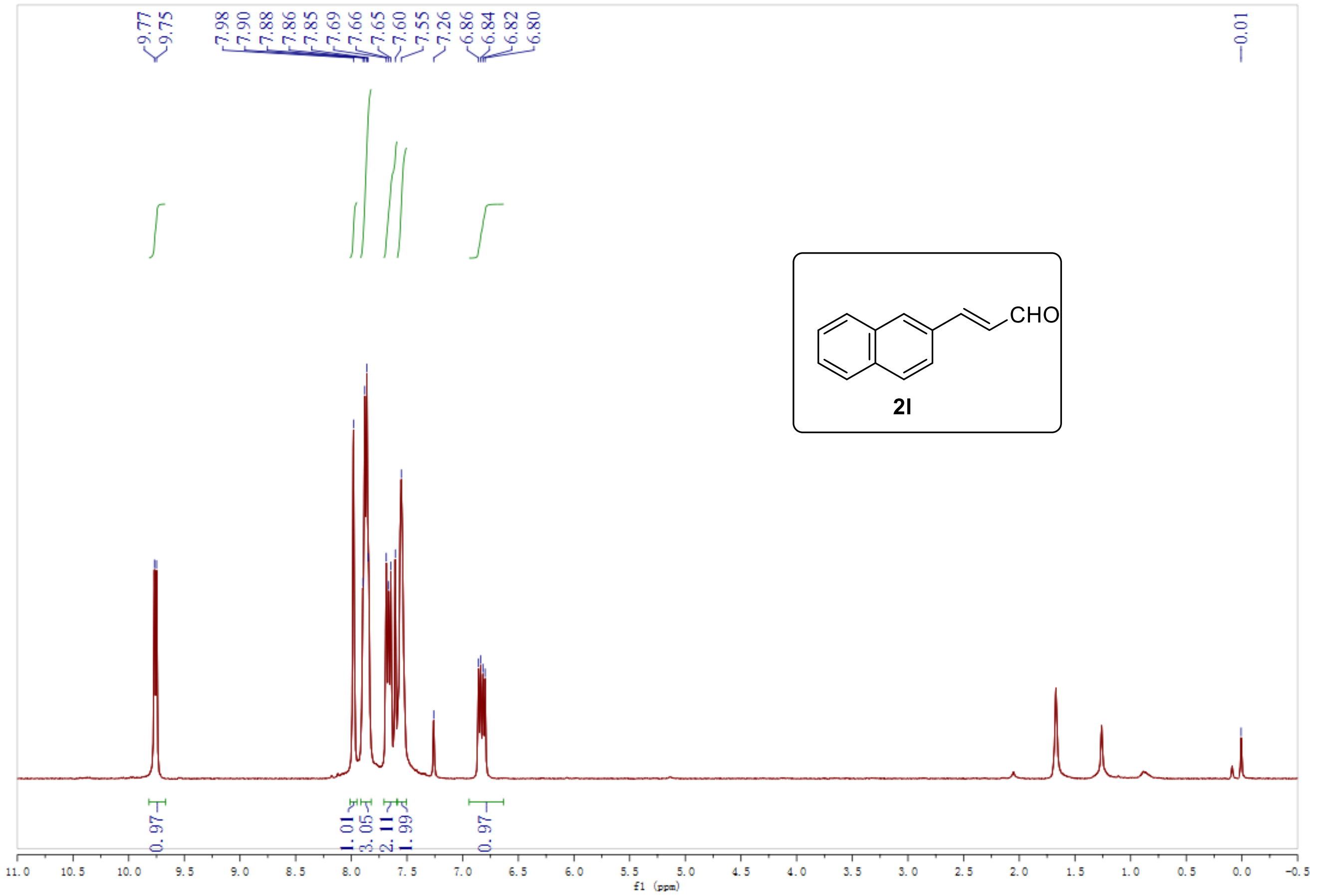
9.77
9.75
7.98
7.90
7.88
7.86
7.85
7.69
7.66
7.65
7.60
7.55
7.26
6.86
6.84
6.82
6.80
-0.01
CHO
2l
0.97
1.01
3.05
2.11
1.99
0.97
f1 (ppm)

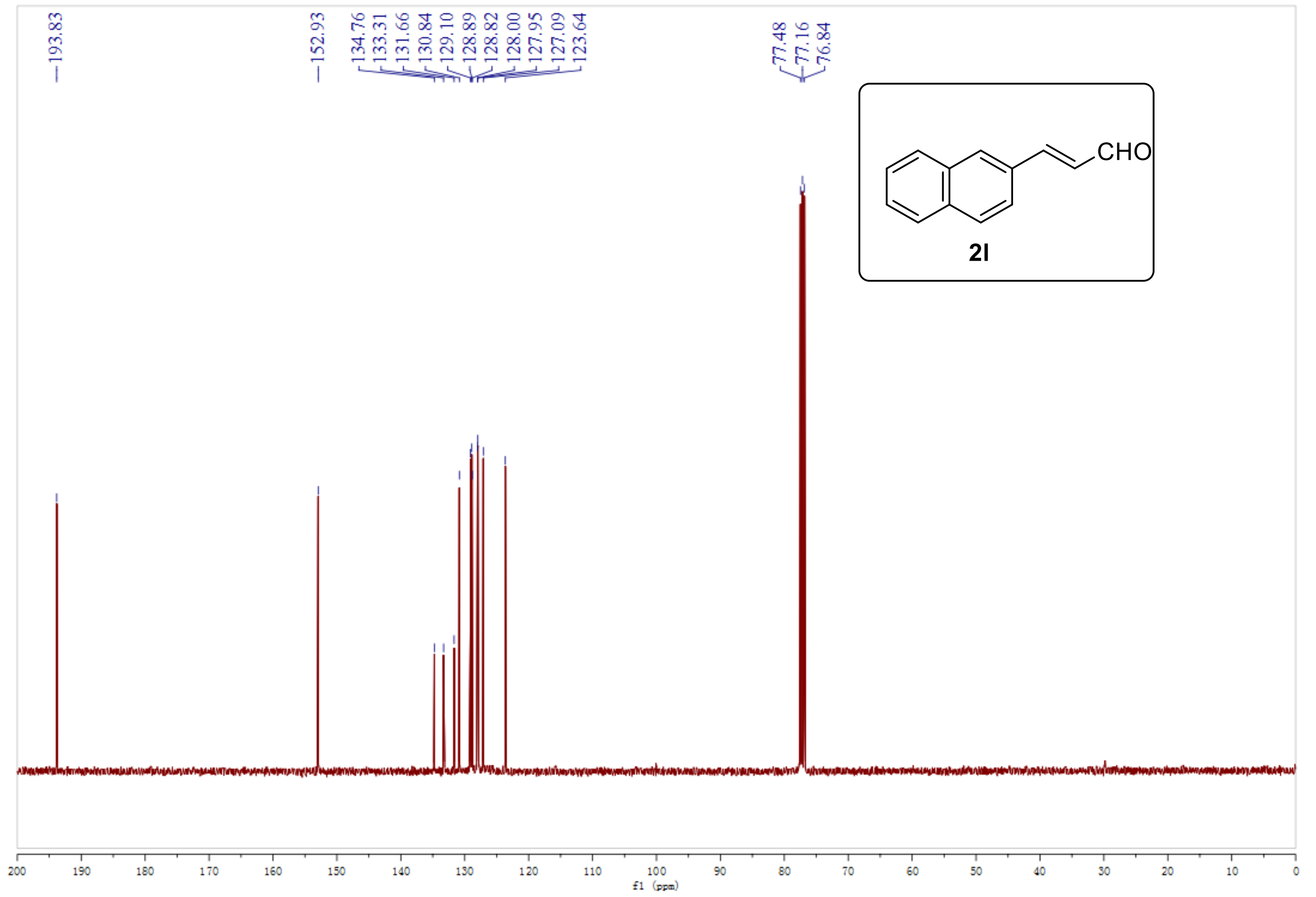
193.83
152.93
134.76
133.31
131.66
130.84
129.10
128.89
128.82
128.00
127.95
127.09
123.64
77.48
77.16
76.84
CHO
2l
f1 (ppm)

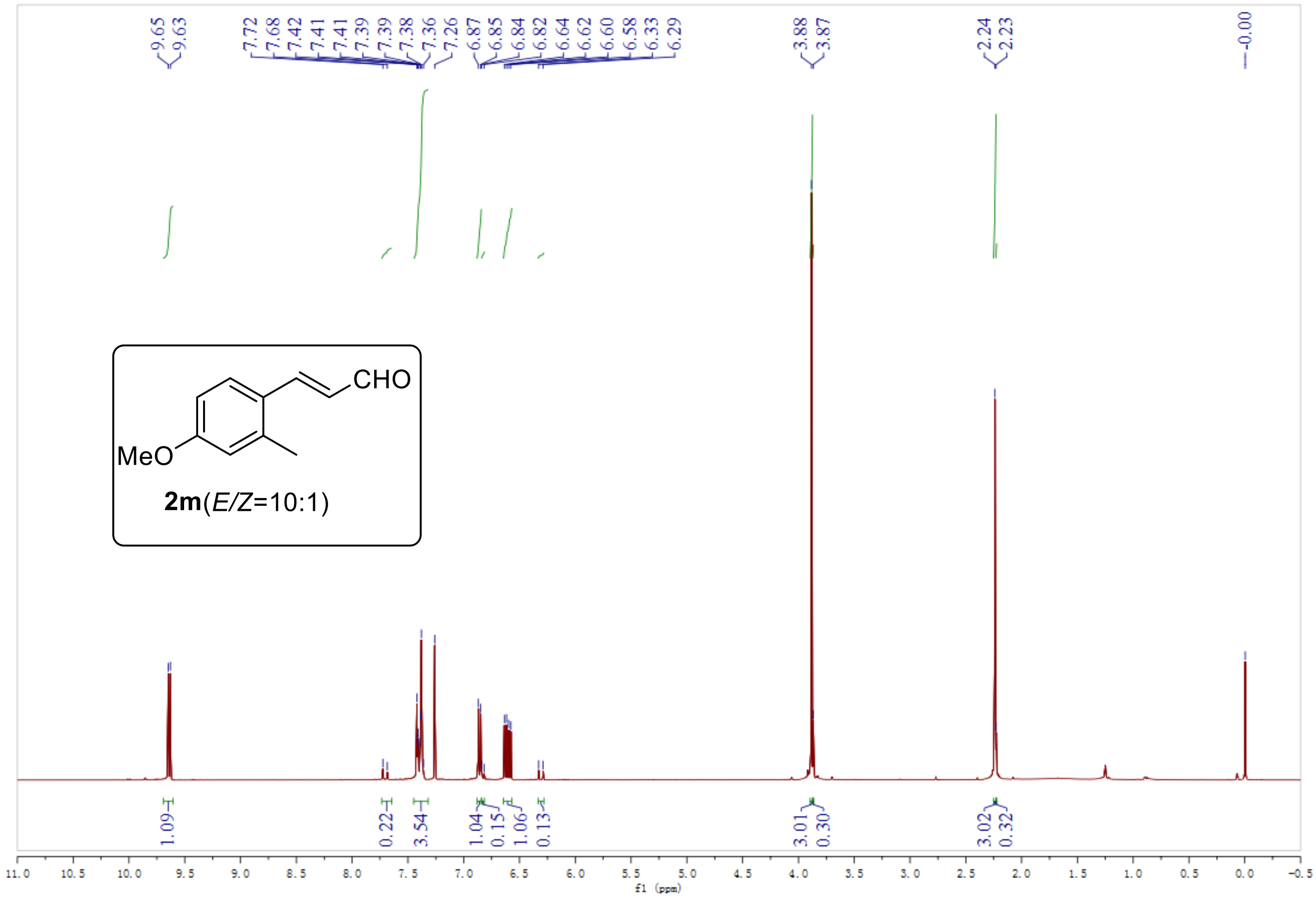
9.65
9.63
7.72
7.68
7.42
7.41
7.41
7.39
7.39
7.38
7.36
7.26
6.87
6.85
6.84
6.82
6.64
6.62
6.60
6.58
6.33
6.29
3.88
3.87
2.24
2.23
-0.00
CHO
MeO
2m(E/Z=10:1)
1.09
0.22
3.54
1.04
0.15
1.06
0.13
3.01
0.30
3.02
0.32
f1 (ppm)

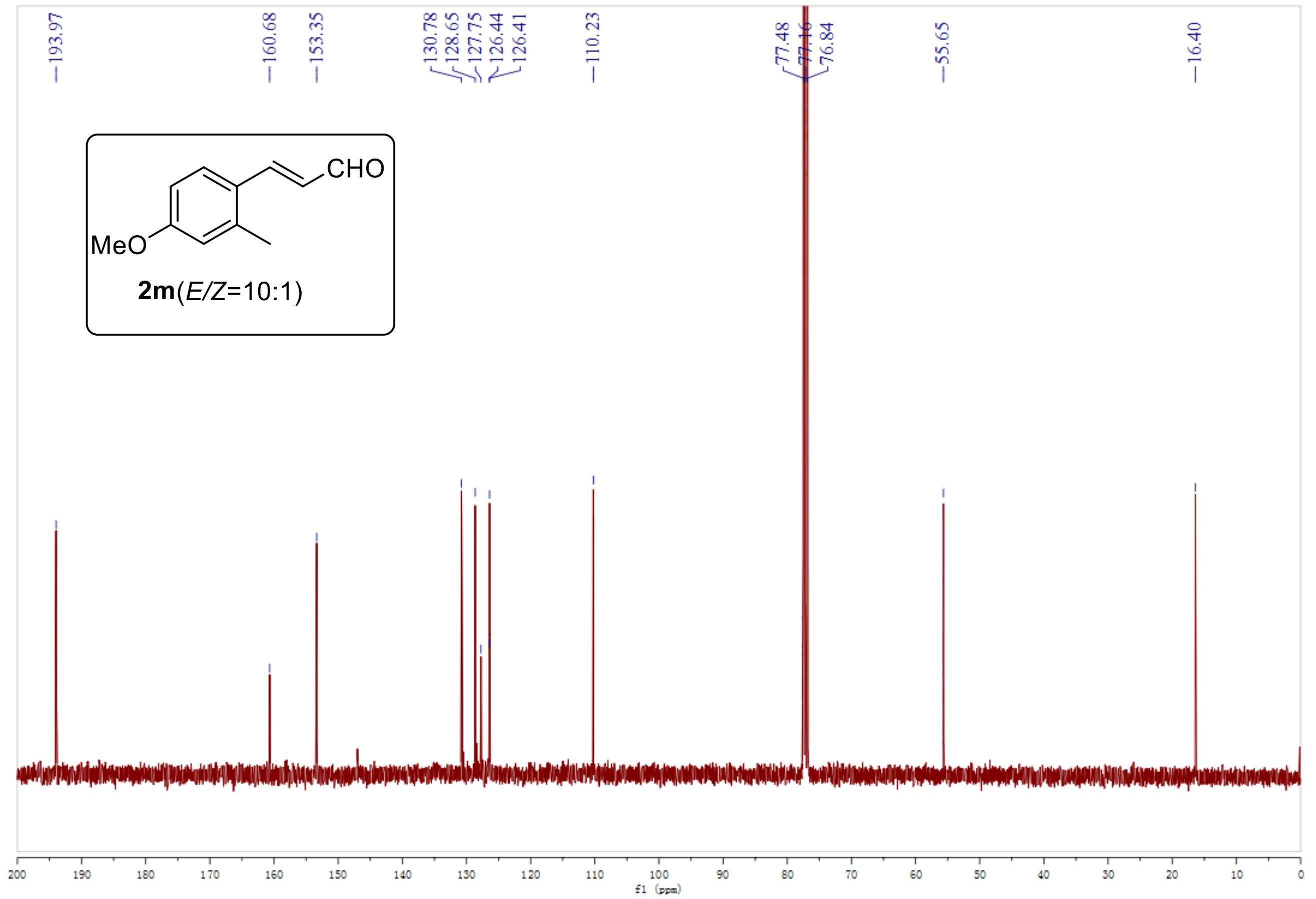
193.97
160.68
153.35
130.78
128.65
127.75
126.44
126.41
110.23
77.48
77.16
76.84
55.65
16.40
CHO
MeO
2m(E/Z=10:1)
f1 (ppm)

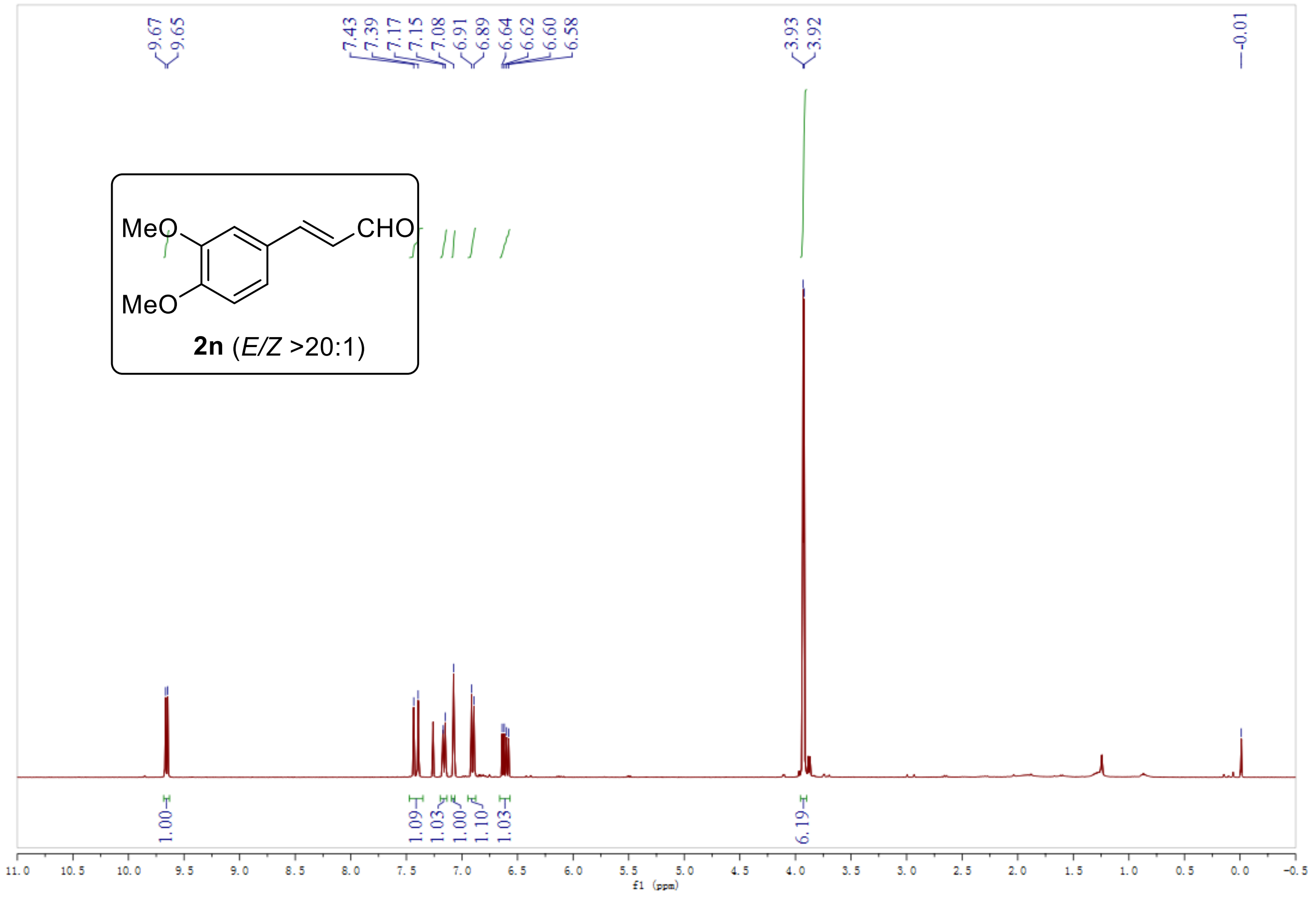
9.67
9.65
7.43
7.39
7.17
7.15
7.08
6.91
6.89
6.64
6.62
6.60
6.58
3.93
3.92
-0.01
MeO
CHO
MeO
2n (E/Z >20:1)
1.00
1.09
1.03
1.00
1.10
1.03
6.19
f1 (ppm)

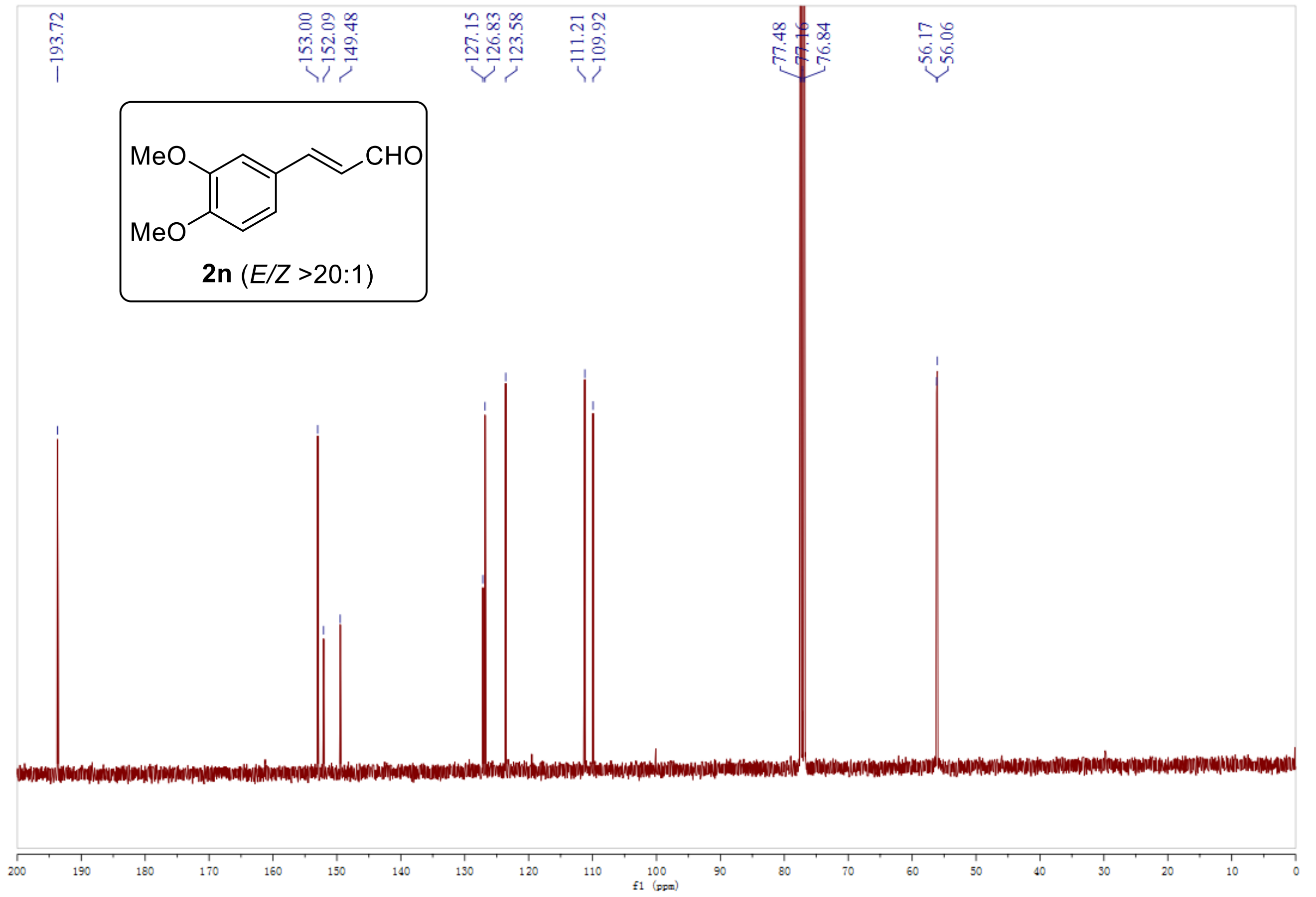
193.72
153.00
152.09
149.48
127.15
126.83
123.58
111.21
109.92
77.48
77.16
76.84
56.17
56.06
MeO
CHO
MeO
2n (E/Z >20:1)
f1 (ppm)

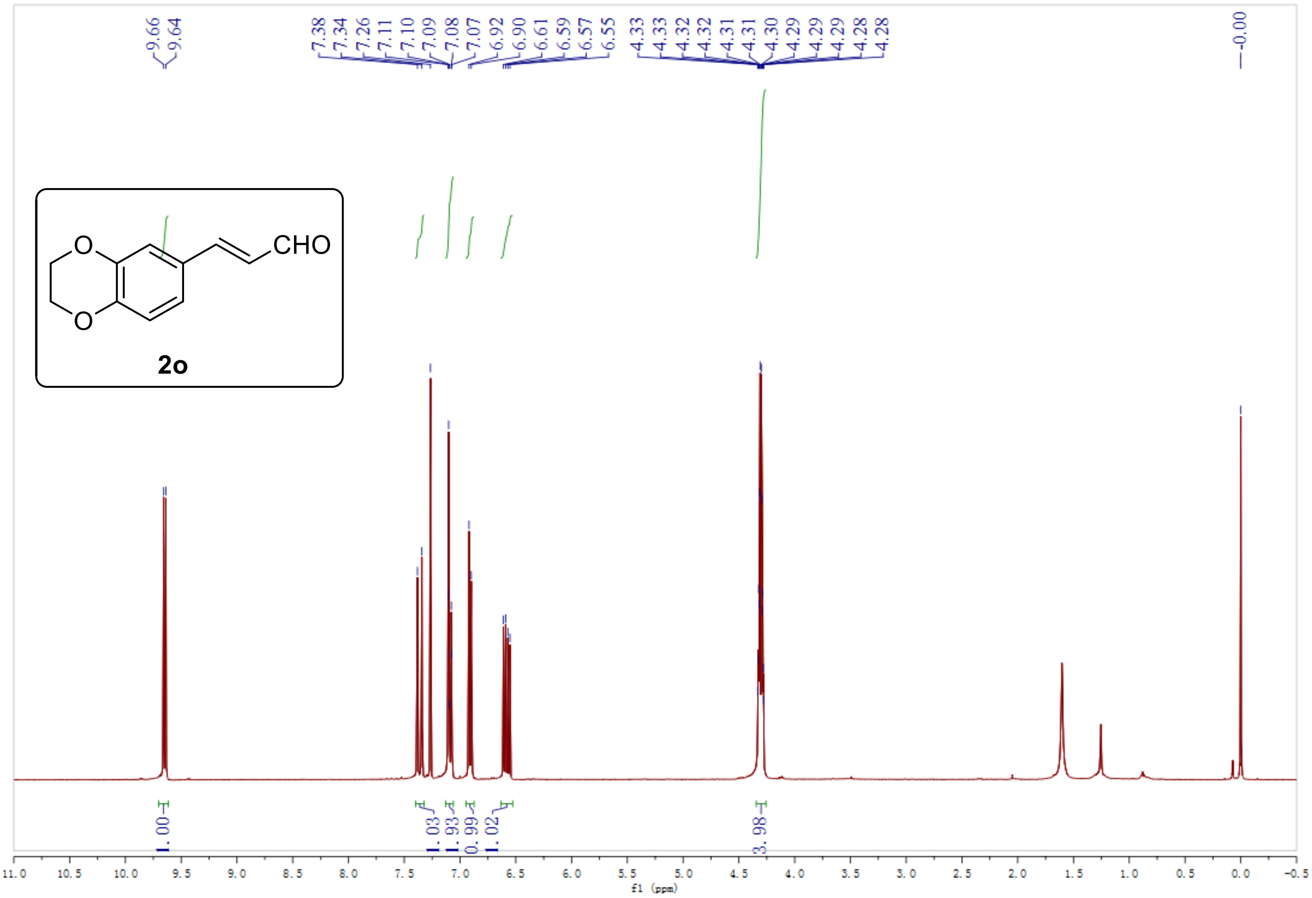
9.66 9.64
7.38 7.34 7.26 7.11 7.10 7.09 7.08 7.07 6.92 6.90 6.61 6.59 6.57 6.55
4.33 4.33 4.32 4.32 4.31 4.31 4.30 4.29 4.29 4.29 4.28 4.28
-0.00
CHO
2o
1.00
1.03
1.93
0.99
1.02
3.98
f1 (ppm)

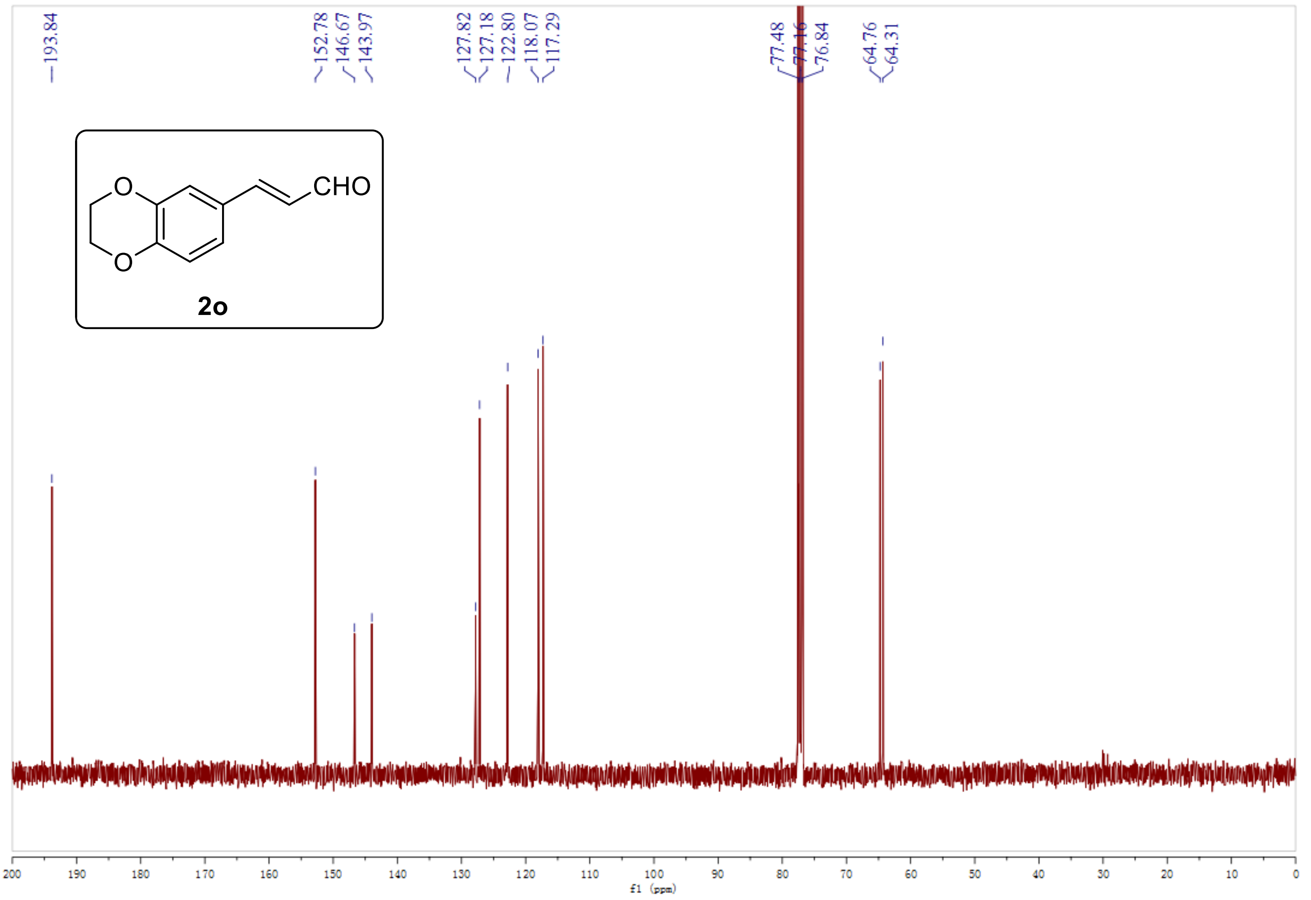
193.84
152.78 146.67 143.97
127.82 127.18 122.80 118.07 117.29
77.48 77.16 76.84
64.76 64.31
CHO
2o
f1 (ppm)

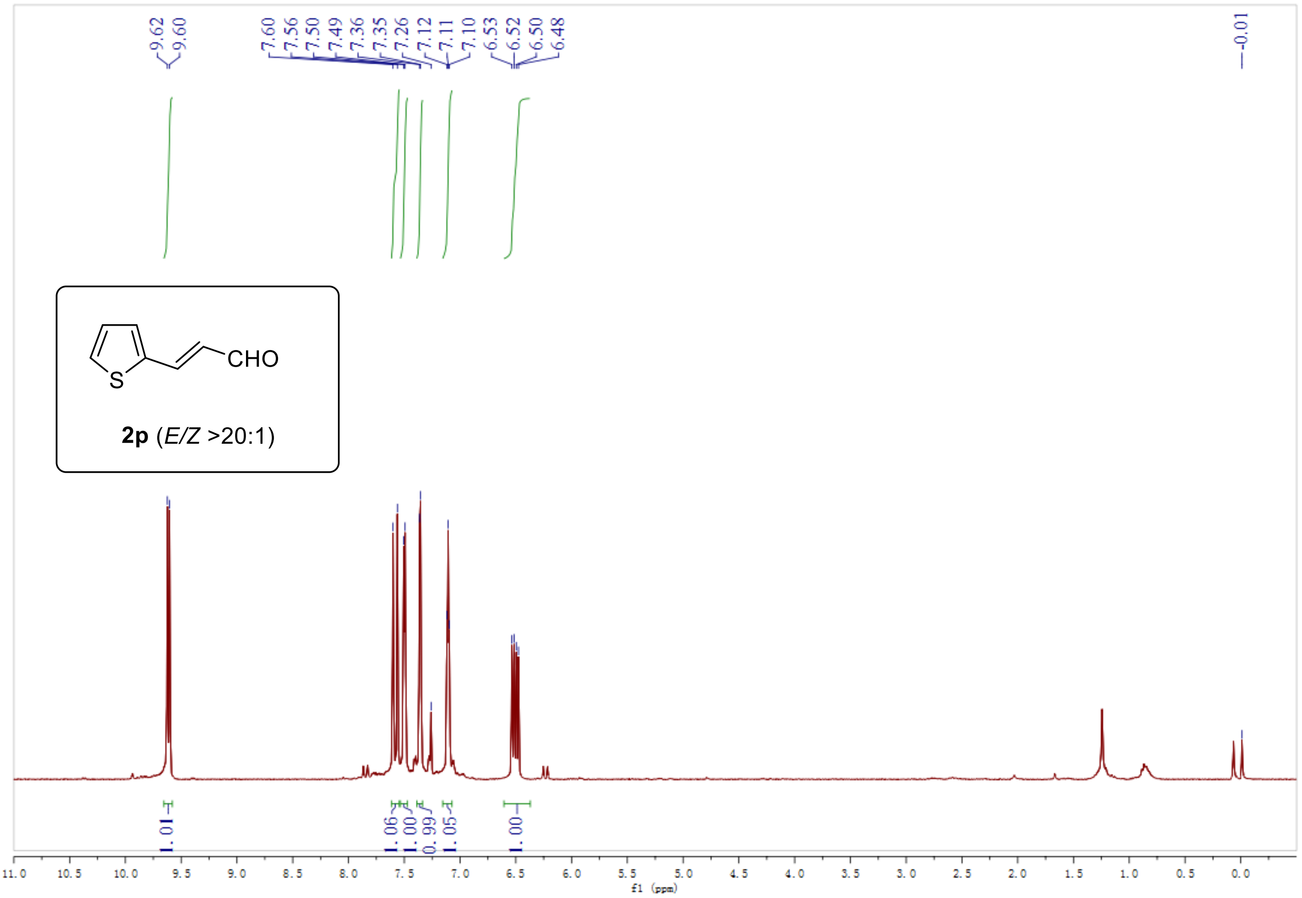
9.62
9.60
7.60
7.56
7.50
7.49
7.36
7.35
7.26
7.12
7.11
7.10
6.53
6.52
6.50
6.48
-0.01
CHO
S
2p (E/Z >20:1)
1.01
1.06
1.00
0.99
1.05
1.00
f1 (ppm)

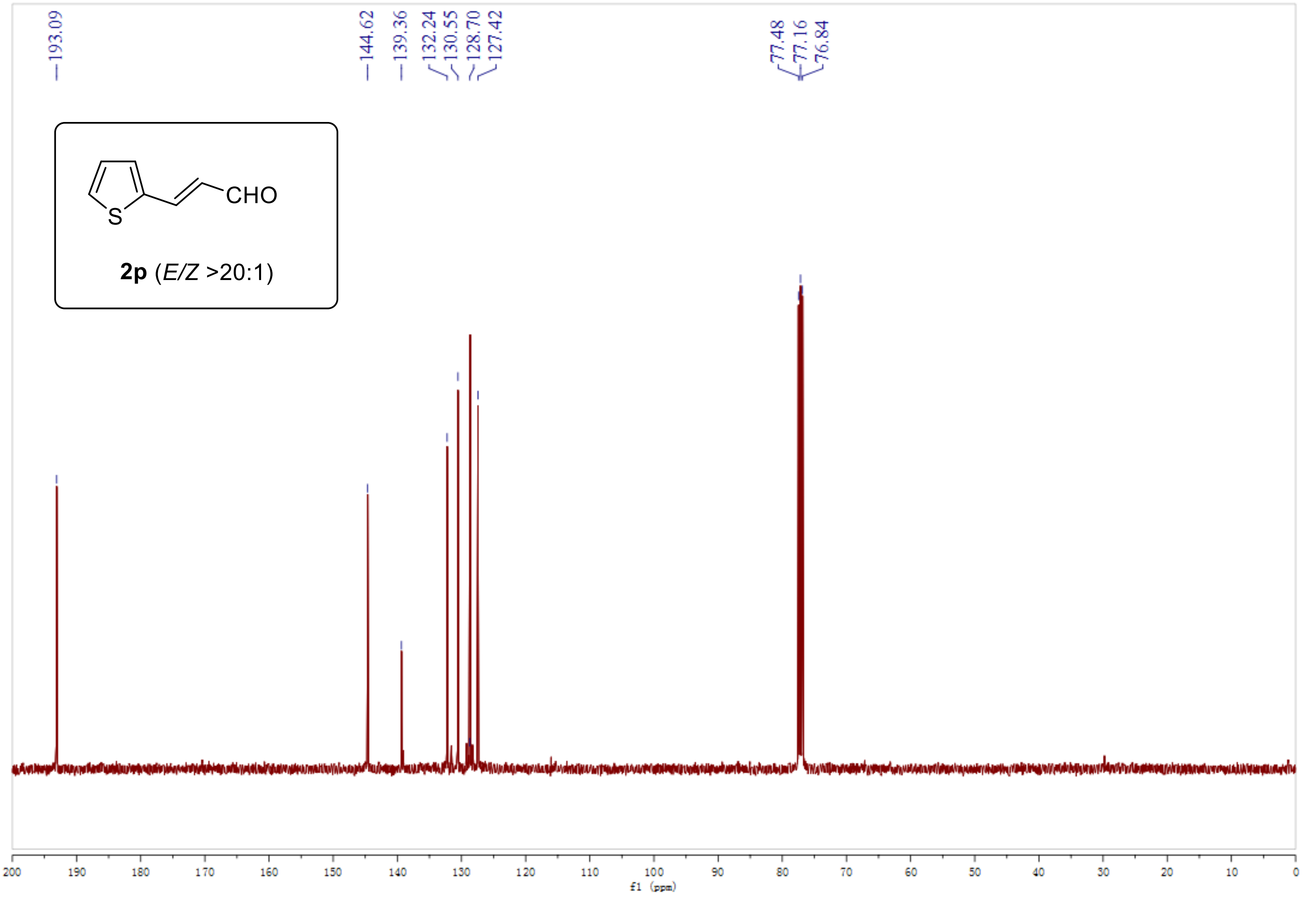
193.09
144.62
139.36
132.24
130.55
128.70
127.42
77.48
77.16
76.84
CHO
S
2p (E/Z >20:1)
f1 (ppm)

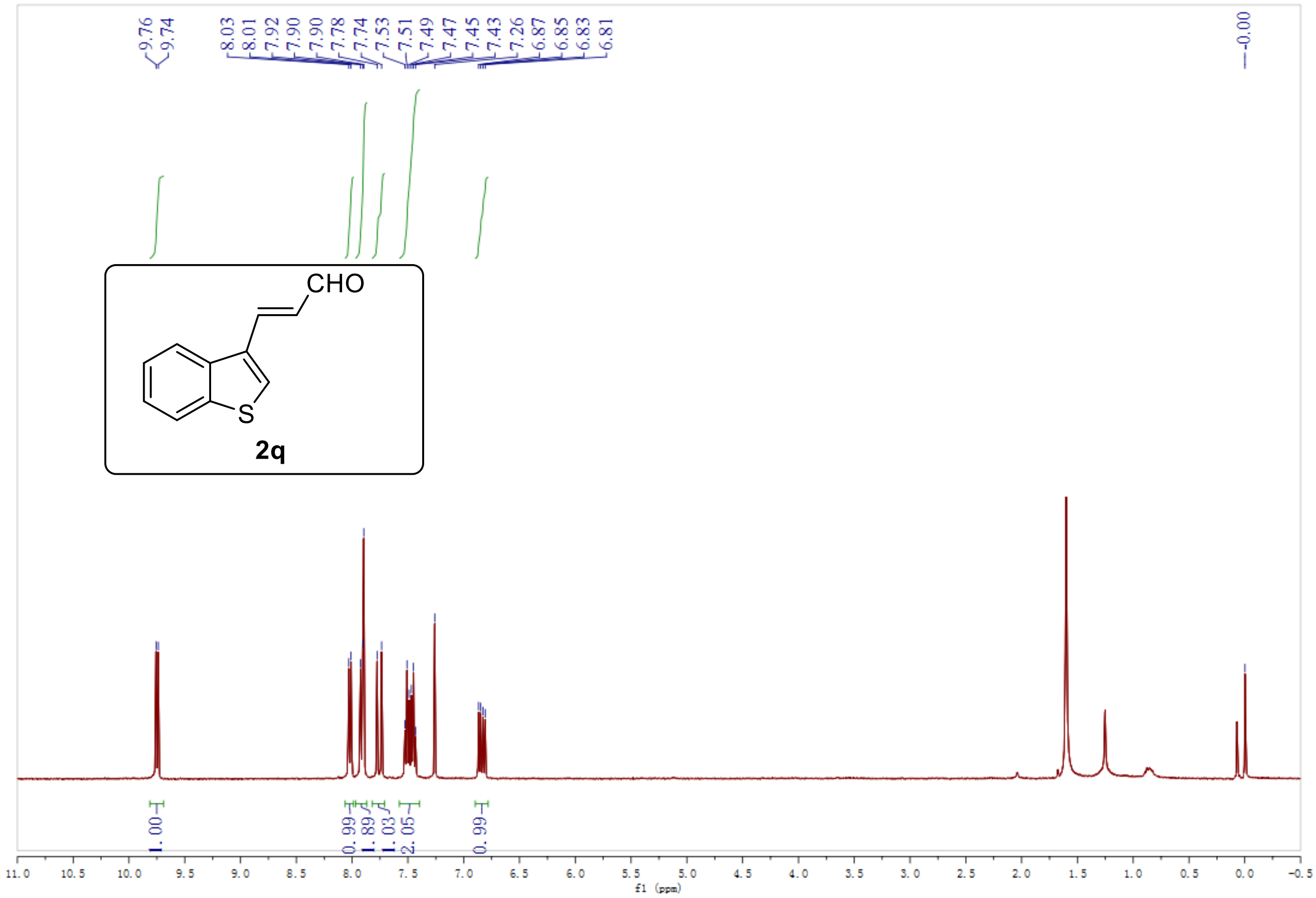

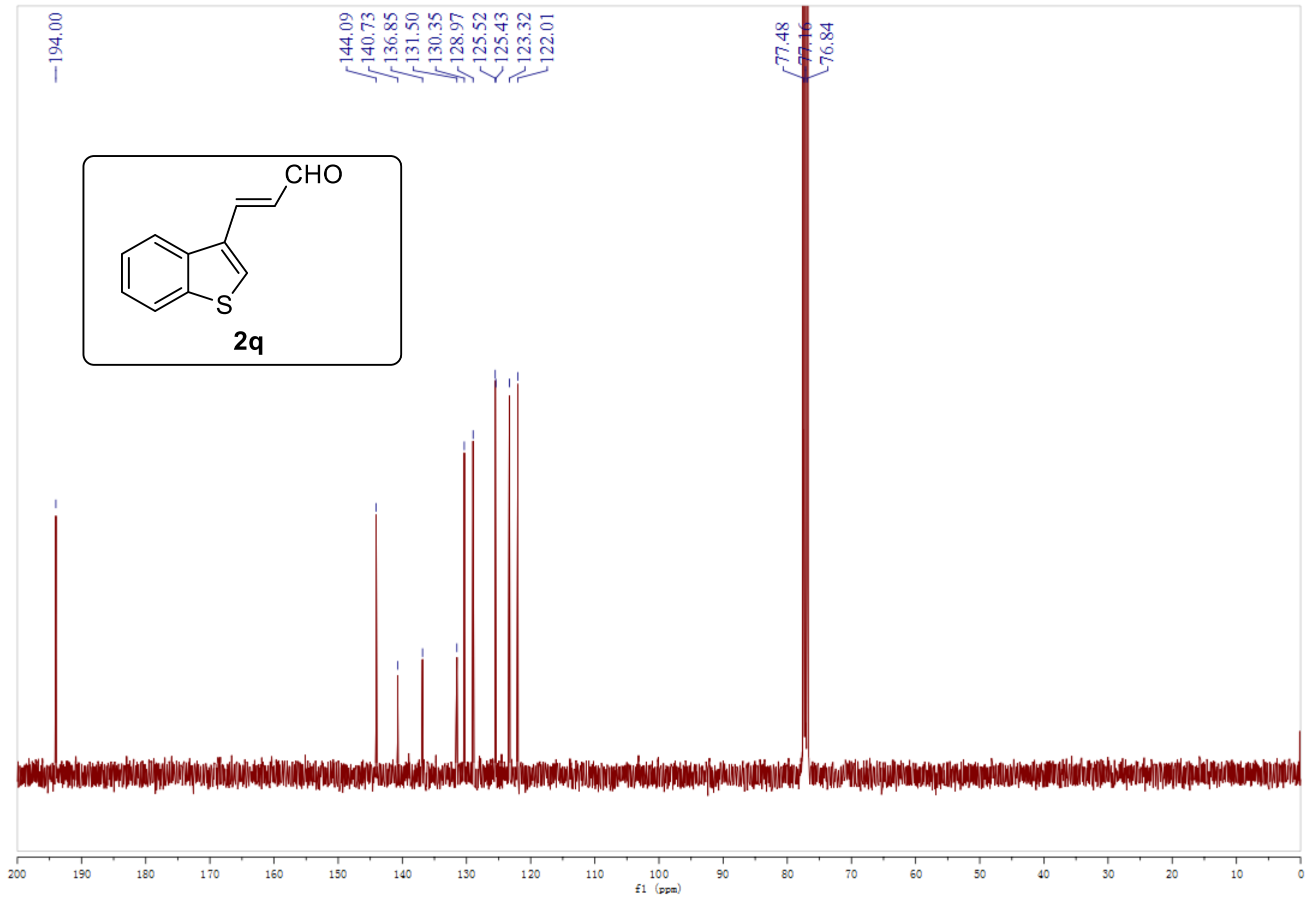

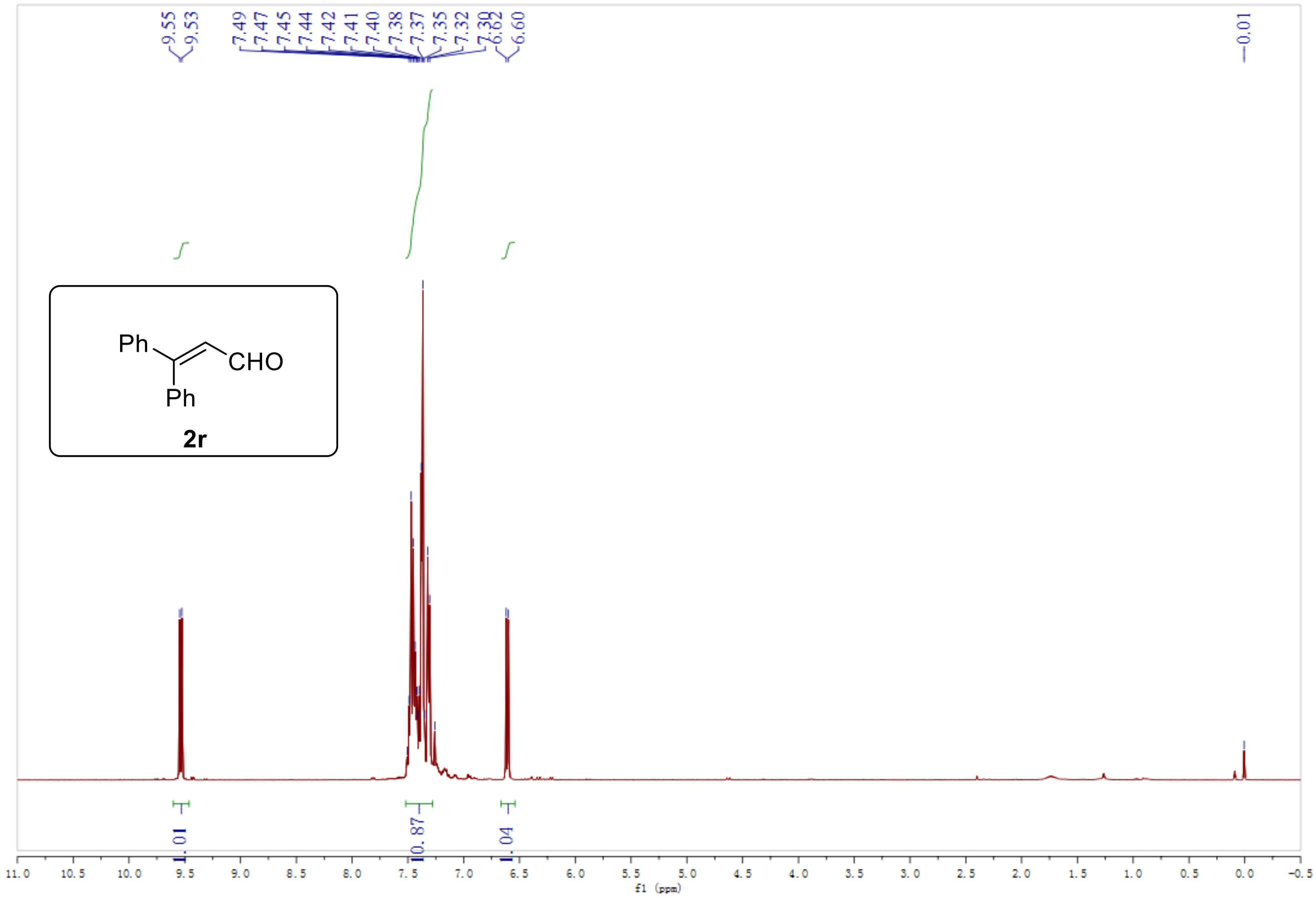

9.55
9.53
7.49
7.47
7.45
7.44
7.42
7.41
7.40
7.38
7.37
7.35
7.32
7.30
6.62
6.60
-0.01
Ph
CHO
Ph
2r
1.01
10.87
1.04
f1 (ppm)

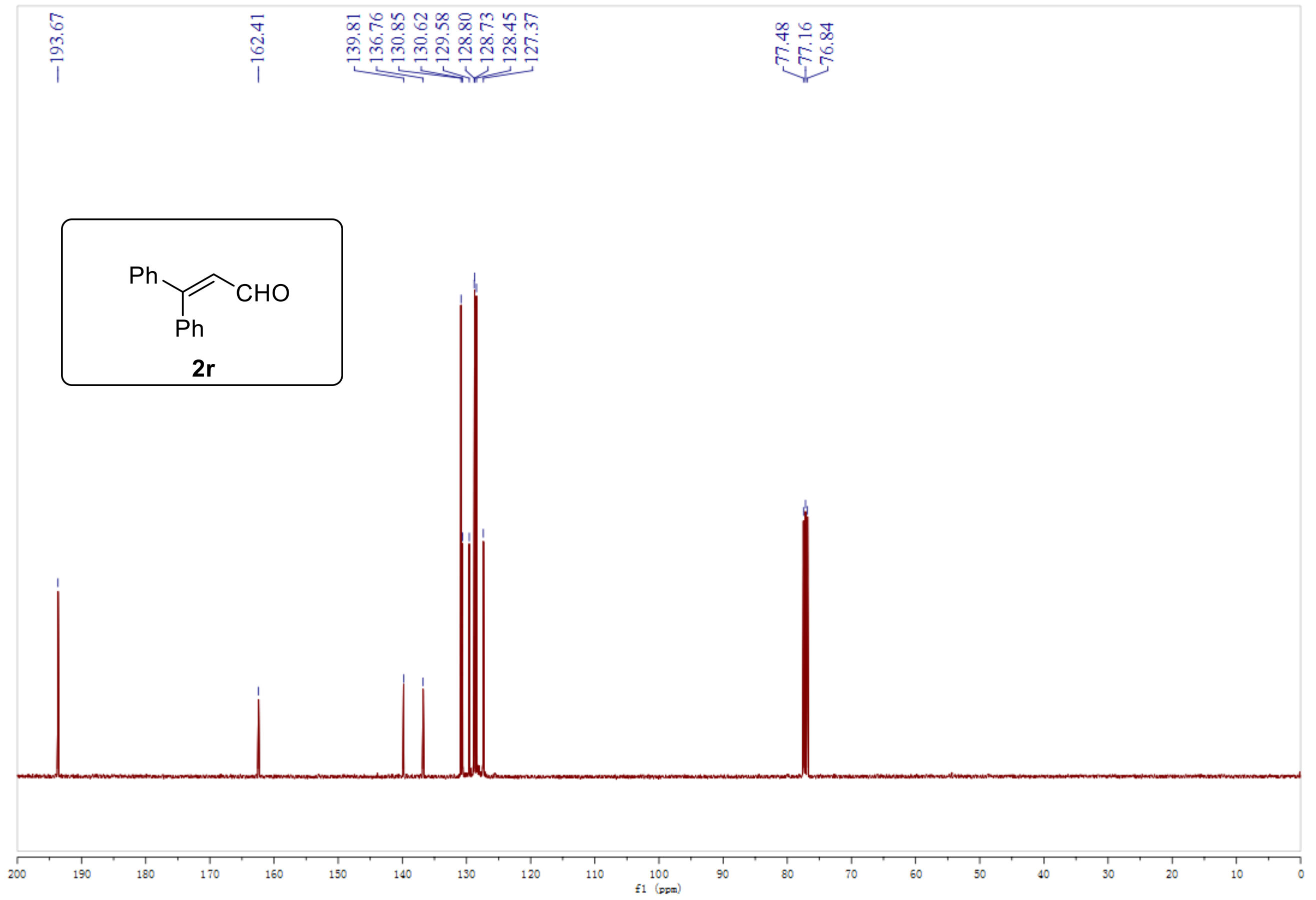

193.67
162.41
139.81
136.76
130.85
130.62
129.58
128.80
128.73
128.45
127.37
77.48
77.16
76.84
Ph
CHO
Ph
2r
f1 (ppm)

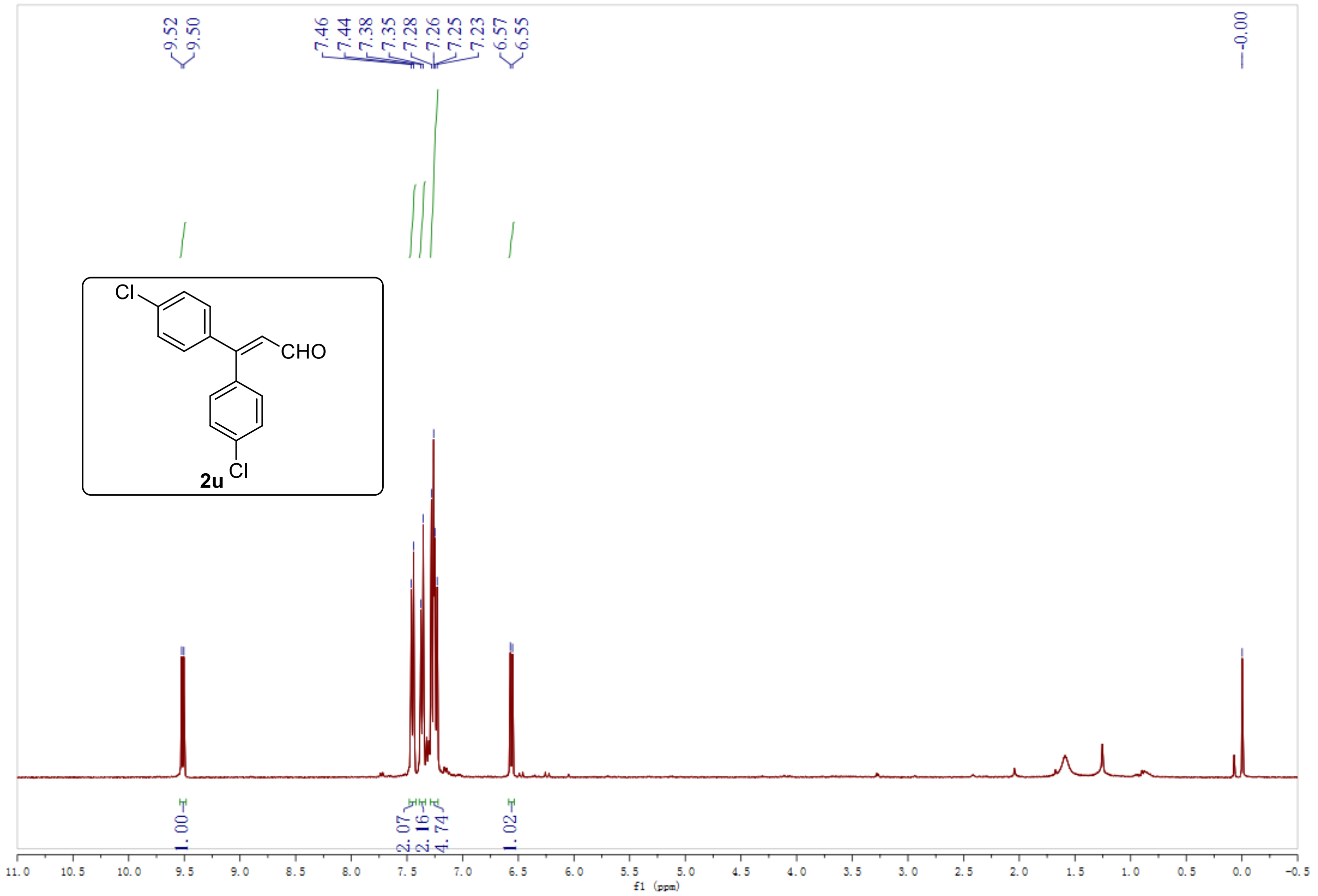

9.52
9.50
7.46
7.44
7.38
7.35
7.28
7.26
7.25
7.23
6.57
6.55
-0.00
Cl
CHO
Cl
2u
1.00
2.07
2.16
4.74
1.02
f1 (ppm)

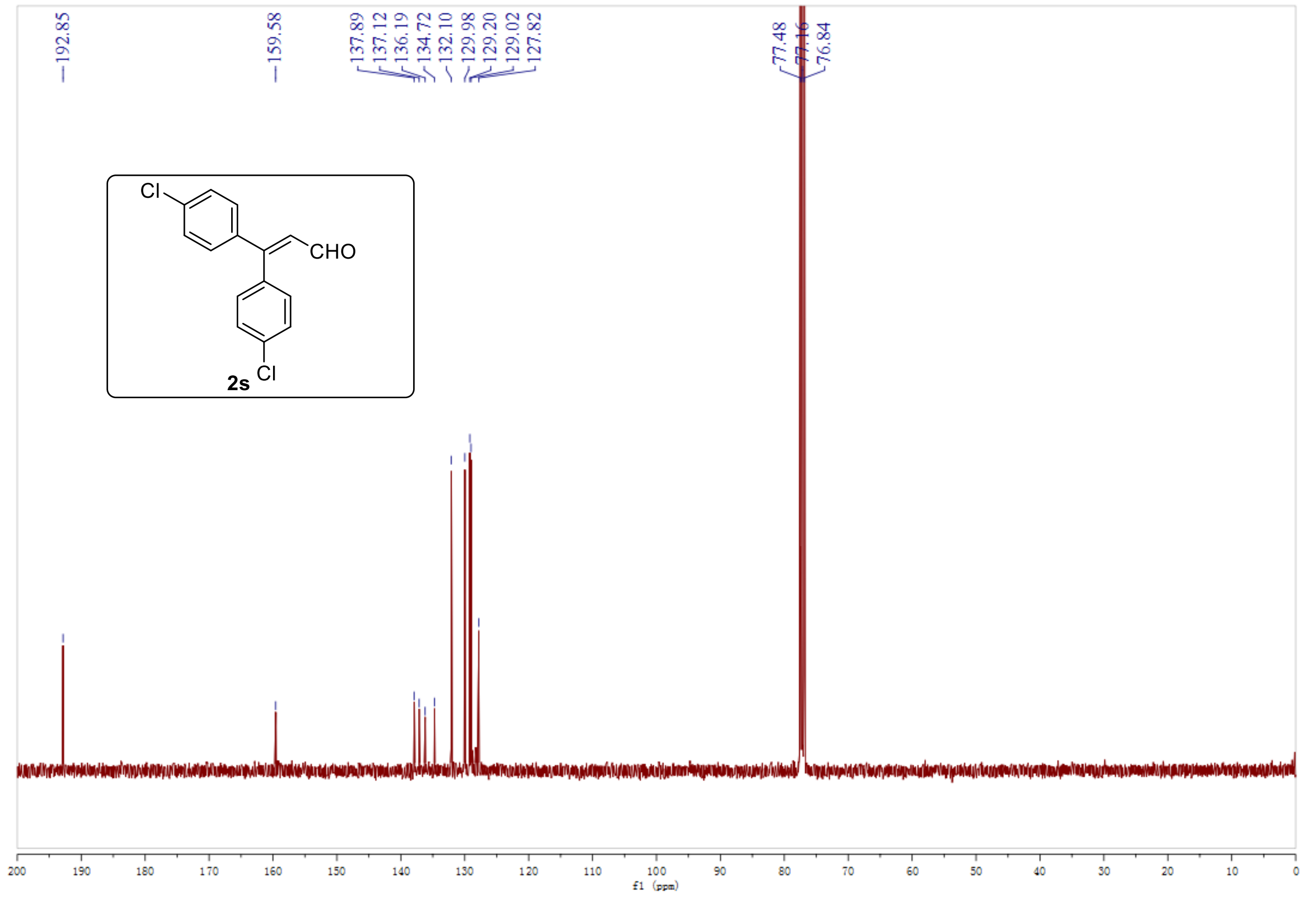

192.85
159.58
137.89
137.12
136.19
134.72
132.10
129.98
129.20
129.02
127.82
77.48
77.16
76.84
Cl
CHO
Cl
2s
f1 (ppm)

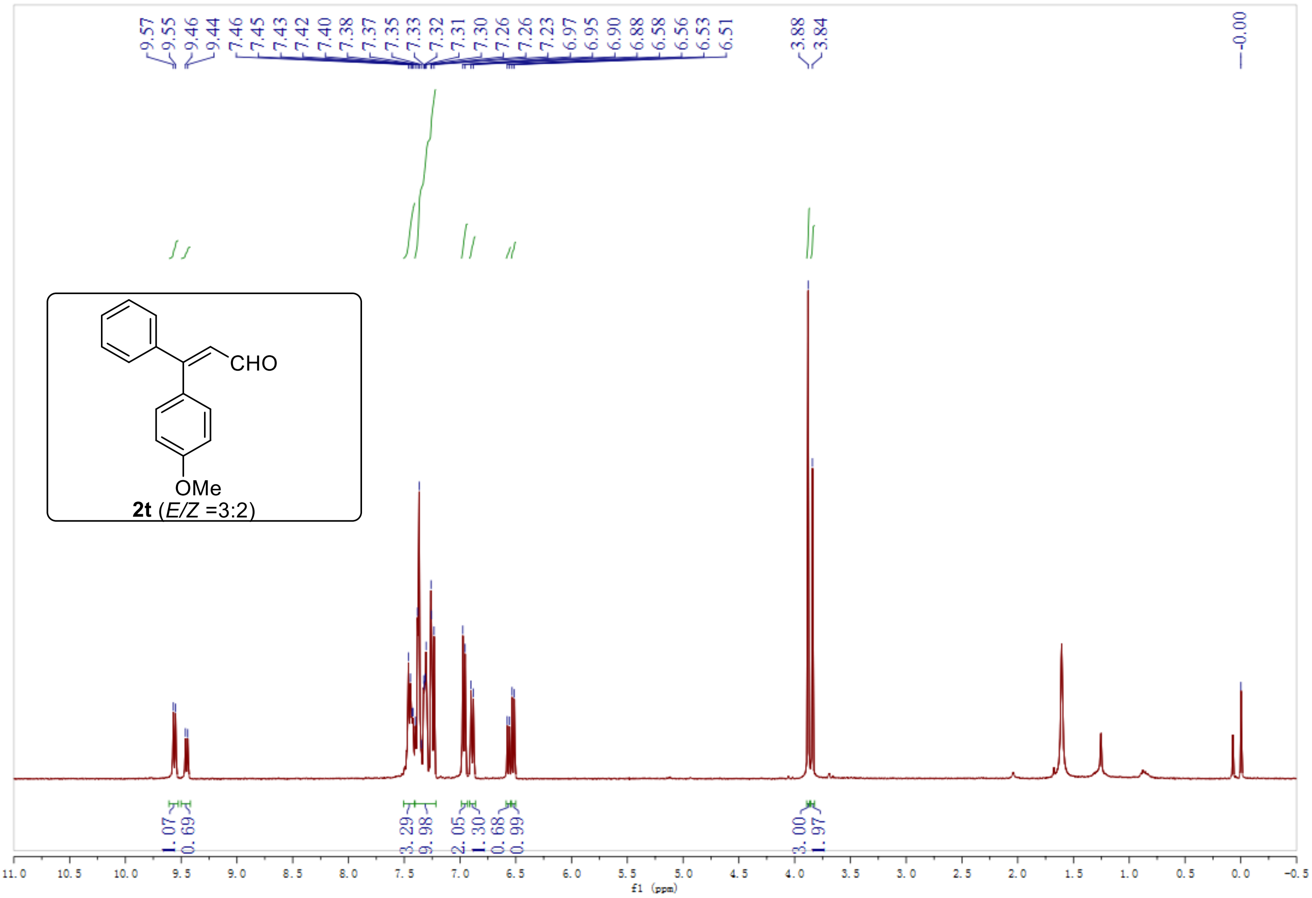
9.57
9.55
9.46
9.44
7.46
7.45
7.43
7.42
7.40
7.38
7.37
7.35
7.33
7.32
7.31
7.30
7.26
7.23
6.97
6.95
6.90
6.88
6.58
6.56
6.53
6.51
3.88
3.84
0.00
CHO
OMe
2t (E/Z =3:2)
1.07
0.69
3.29
9.98
2.05
1.30
0.68
0.99
3.00
1.97
f1 (ppm)

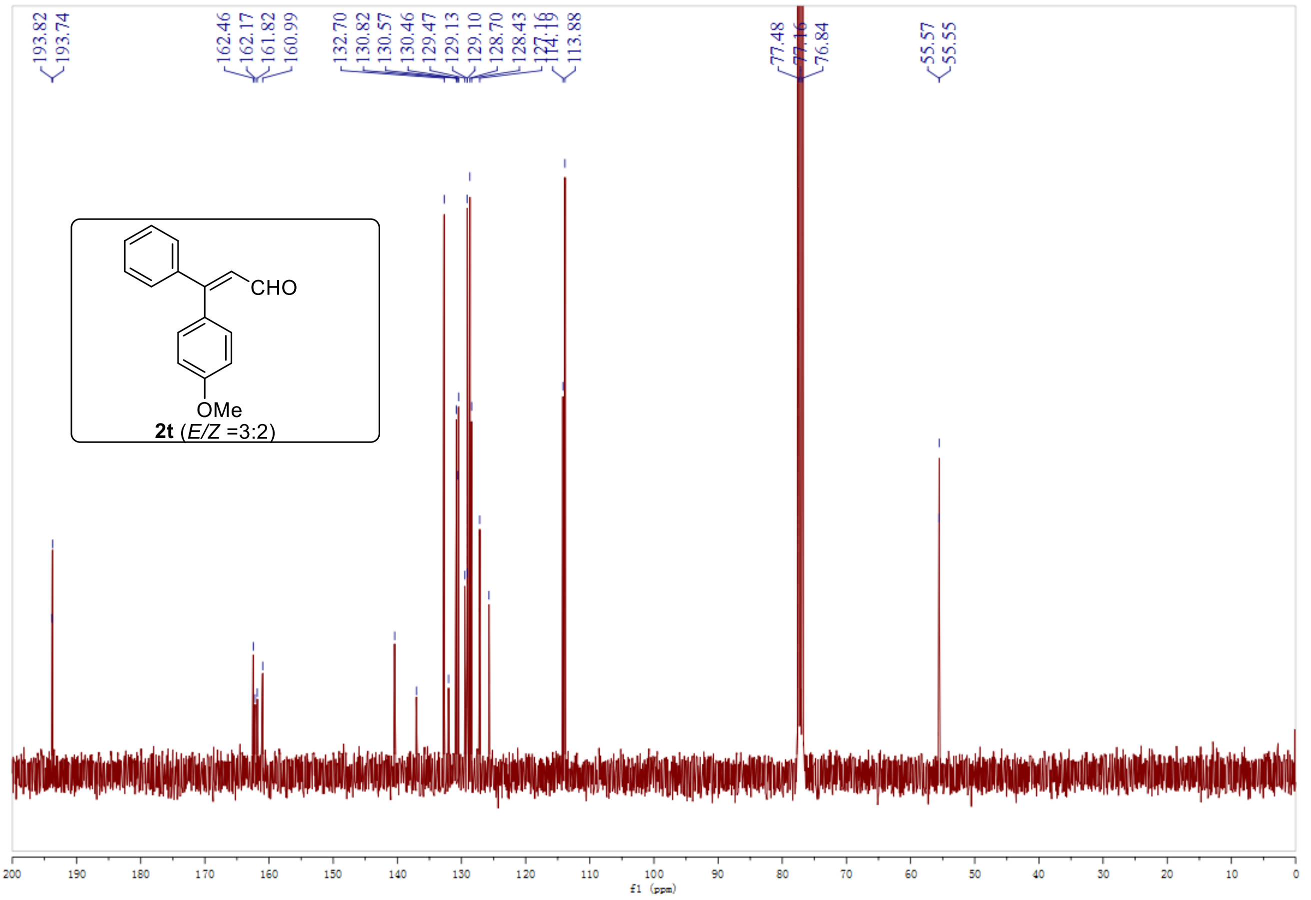
193.82
193.74
162.46
162.17
161.82
160.99
132.70
130.82
130.57
130.46
129.47
129.13
129.10
128.70
128.43
127.16
114.18
113.88
77.48
77.16
76.84
55.57
55.55
CHO
OMe
2t (E/Z =3:2)
f1 (ppm)

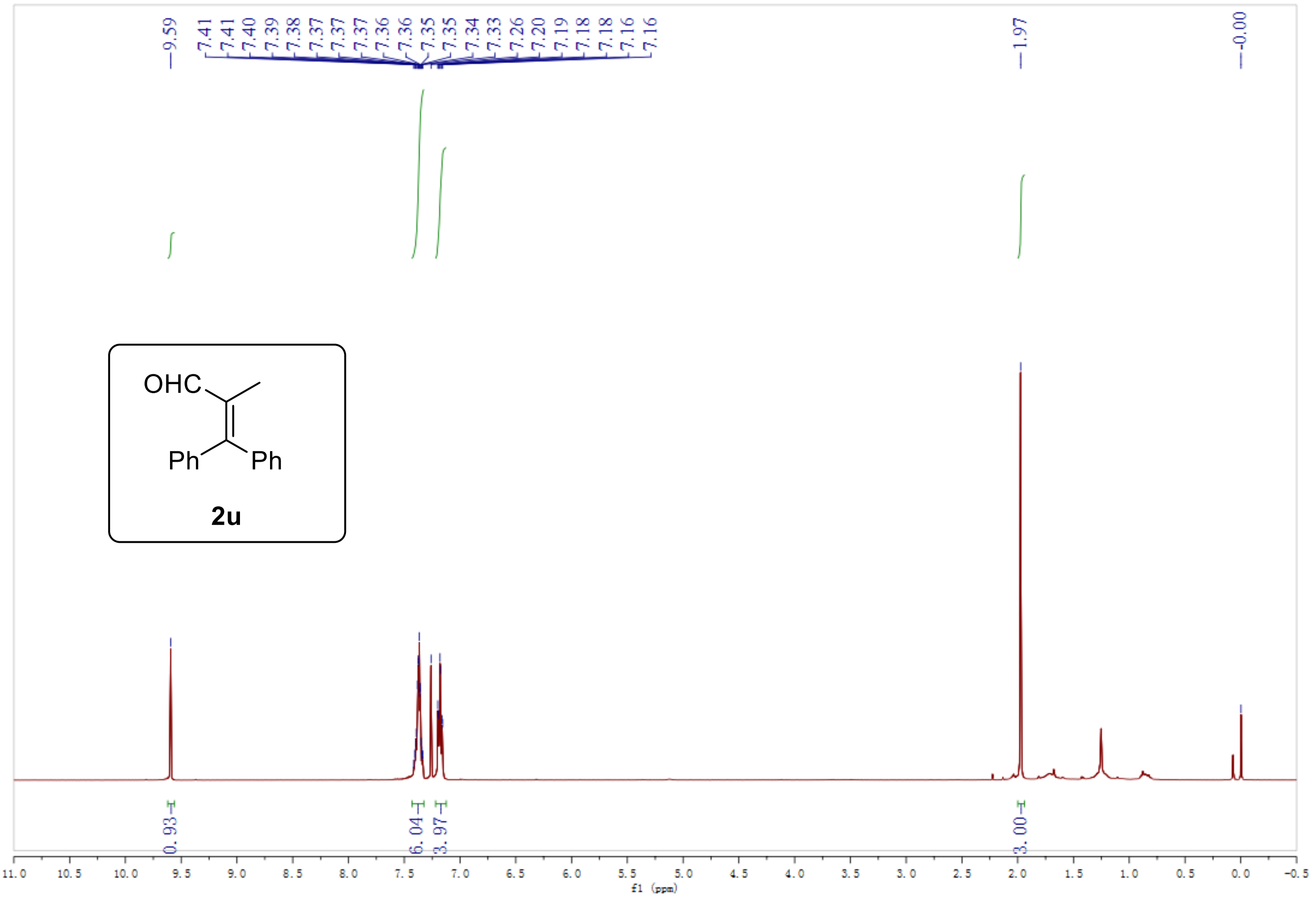
9.59
7.41
7.41
7.40
7.39
7.38
7.37
7.37
7.37
7.36
7.36
7.35
7.35
7.34
7.33
7.26
7.20
7.19
7.18
7.16
7.16
1.97
-0.00
OHC
Ph
Ph
2u
0.93
6.04
3.97
3.00
f1 (ppm)

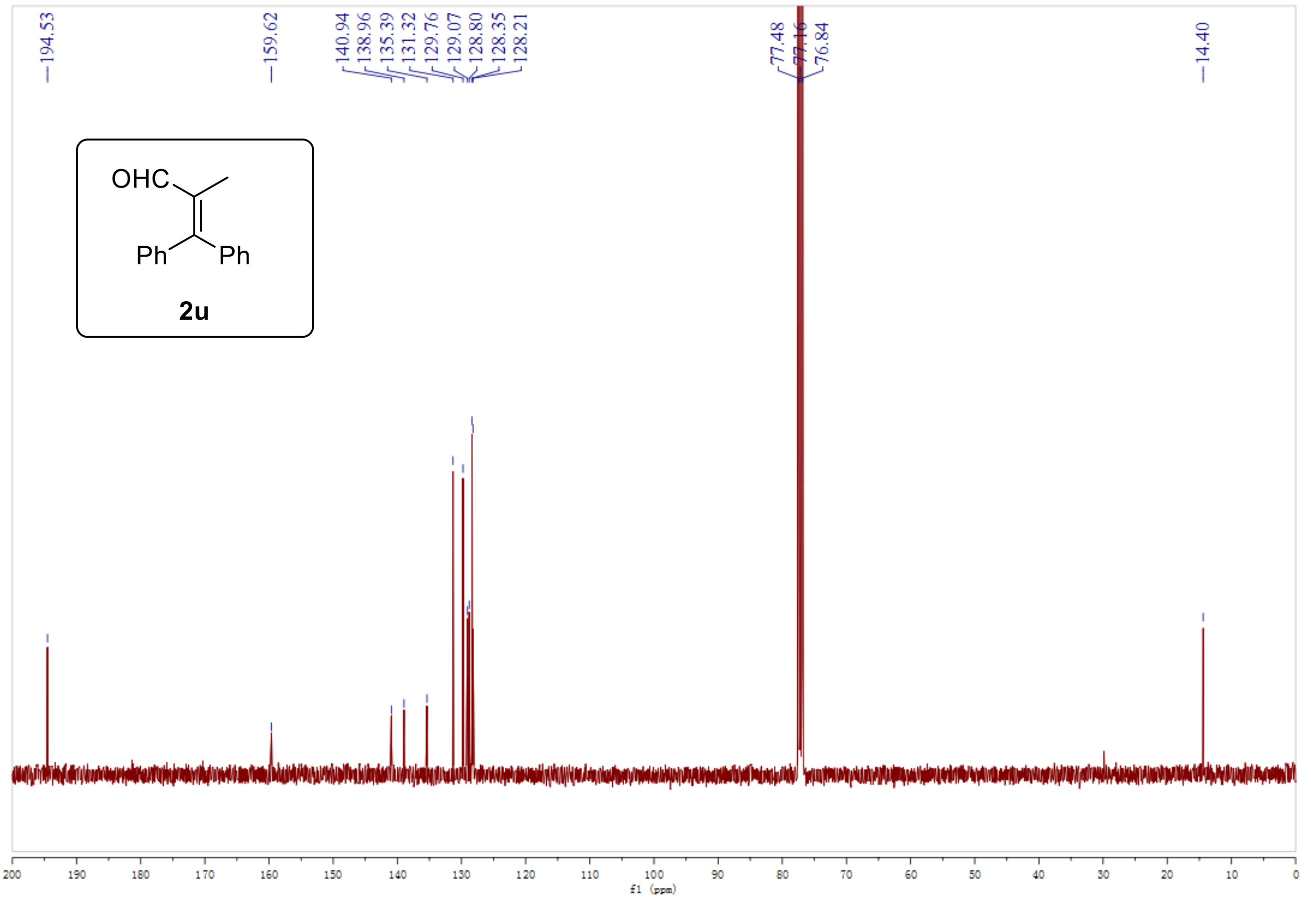
194.53
159.62
140.94
138.96
135.39
131.32
129.76
129.07
128.80
128.35
128.21
77.48
77.16
76.84
14.40
OHC
Ph
Ph
2u
f1 (ppm)

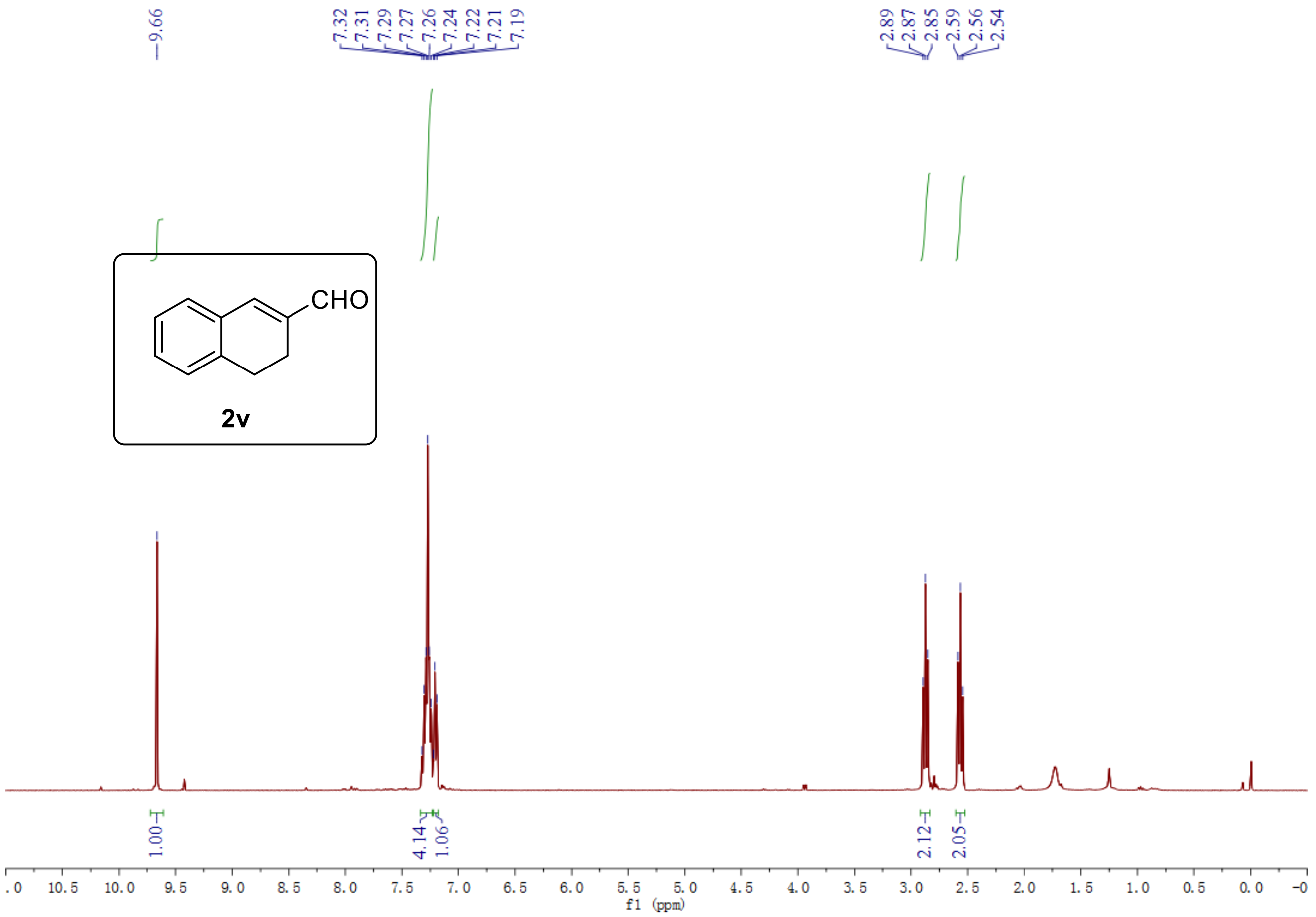
9.66
7.32
7.31
7.29
7.27
7.26
7.24
7.22
7.21
7.19
2.89
2.87
2.85
2.59
2.56
2.54
CHO
2v
1.00
4.14
1.06
2.12
2.05
f1 (ppm)

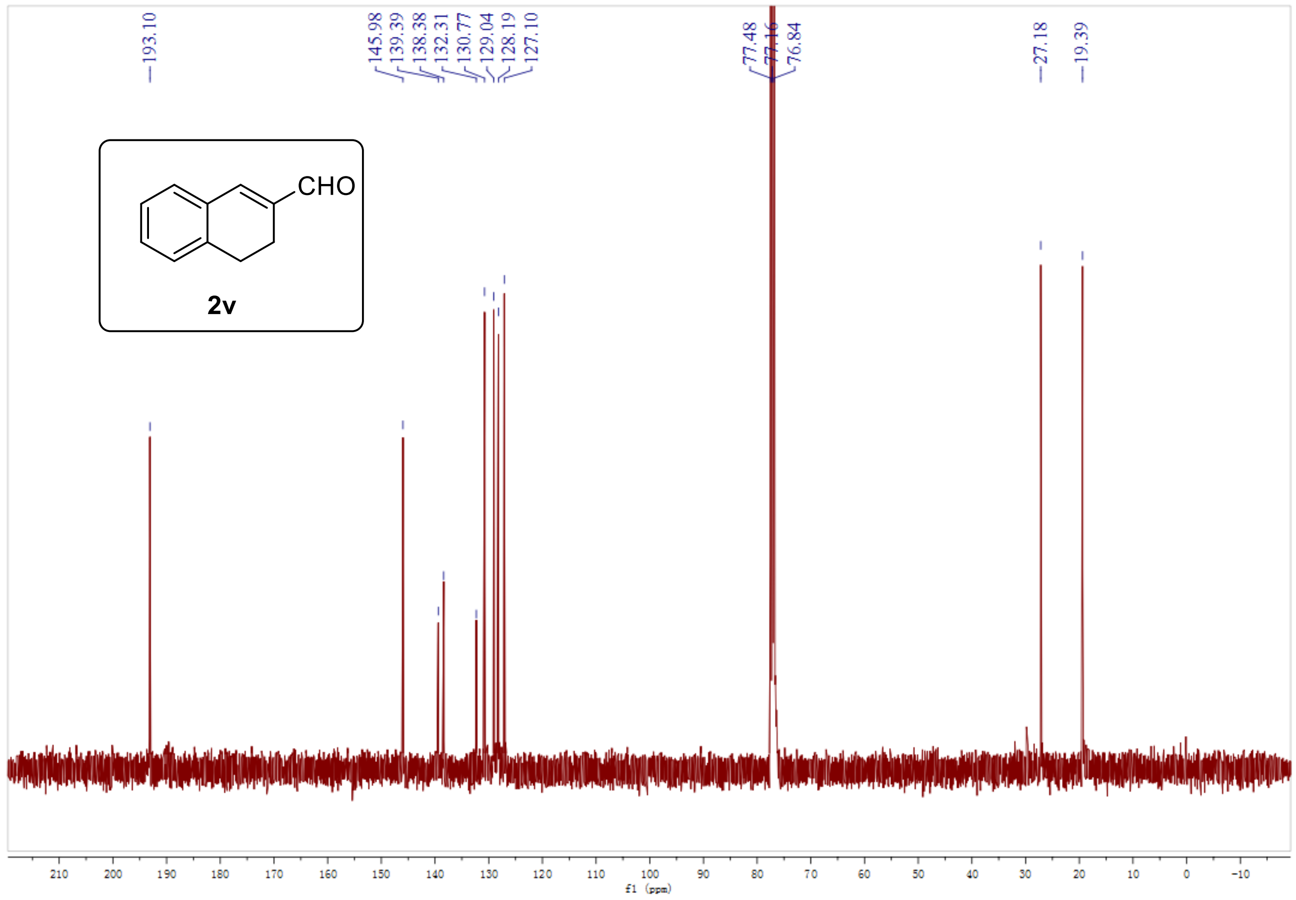
193.10
145.98
139.39
138.38
132.31
130.77
129.04
128.19
127.10
77.48
77.16
76.84
27.18
19.39
CHO
2v
f1 (ppm)

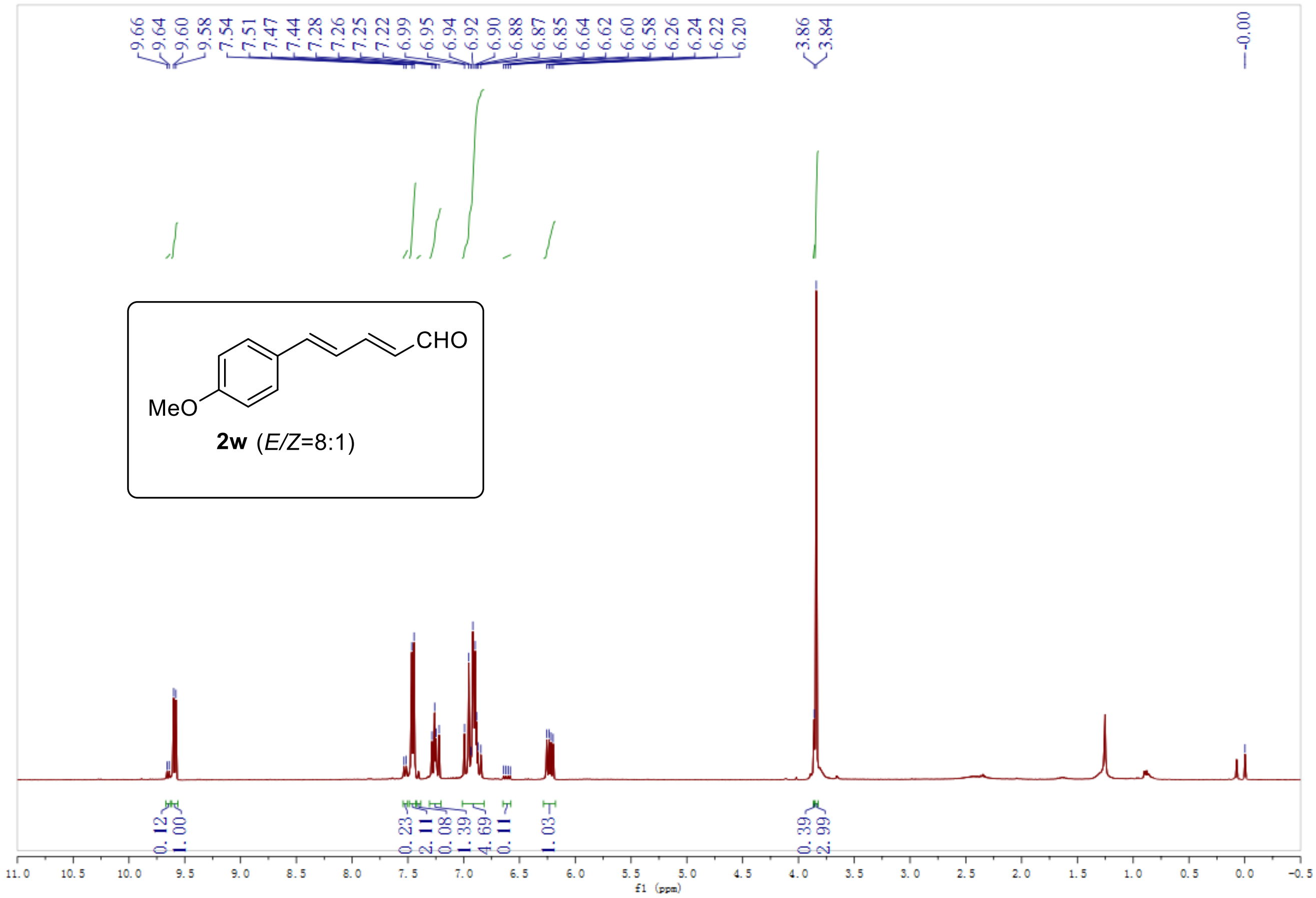
9.66
9.64
9.60
9.58
7.54
7.51
7.47
7.44
7.28
7.26
7.25
7.22
6.99
6.95
6.94
6.92
6.90
6.88
6.87
6.85
6.64
6.62
6.60
6.58
6.26
6.24
6.22
6.20
3.86
3.84
0.00
CHO
MeO
2w (E/Z=8:1)
f1 (ppm)

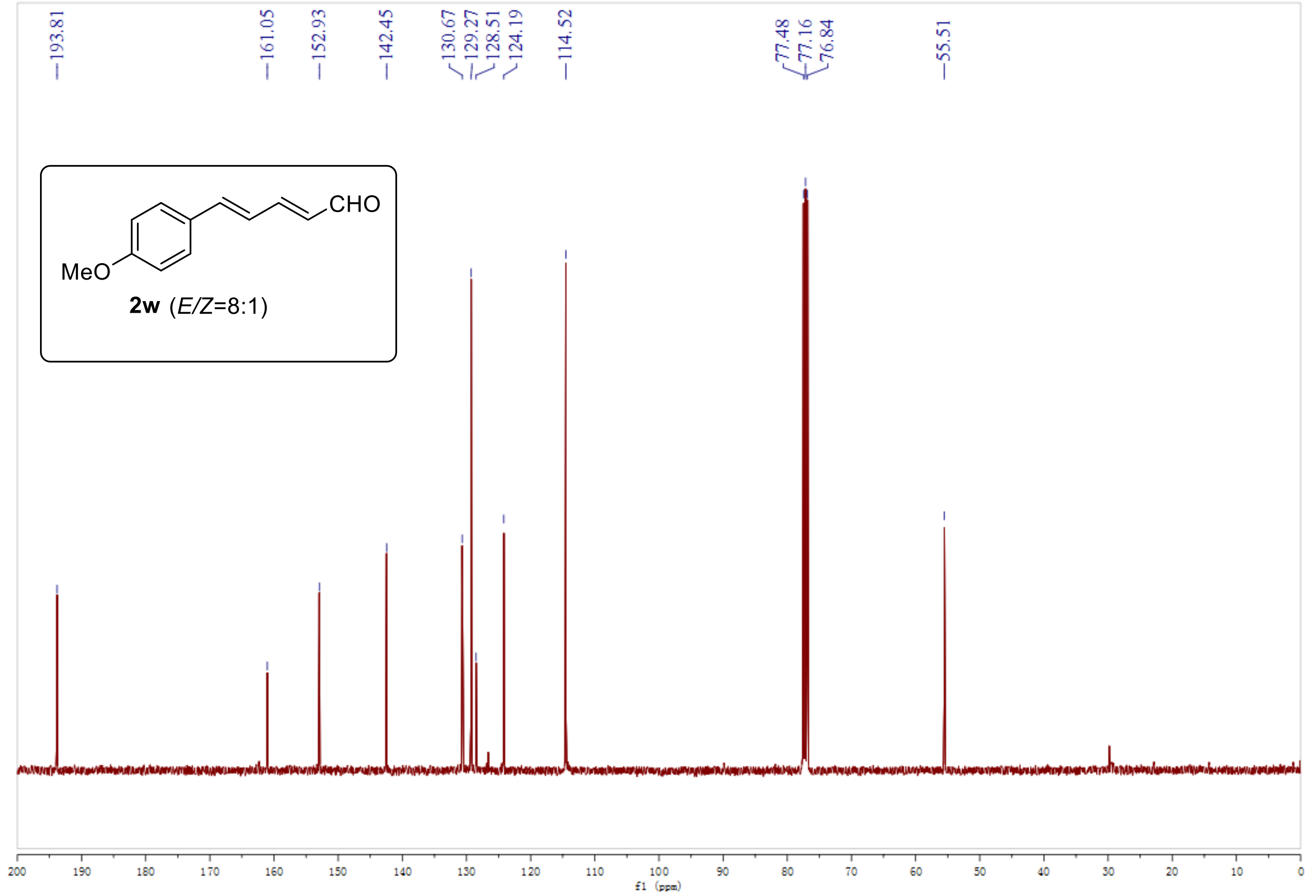
193.81
161.05
152.93
142.45
130.67
129.27
128.51
124.19
114.52
77.48
77.16
76.84
55.51
CHO
MeO
2w (E/Z=8:1)
f1 (ppm)

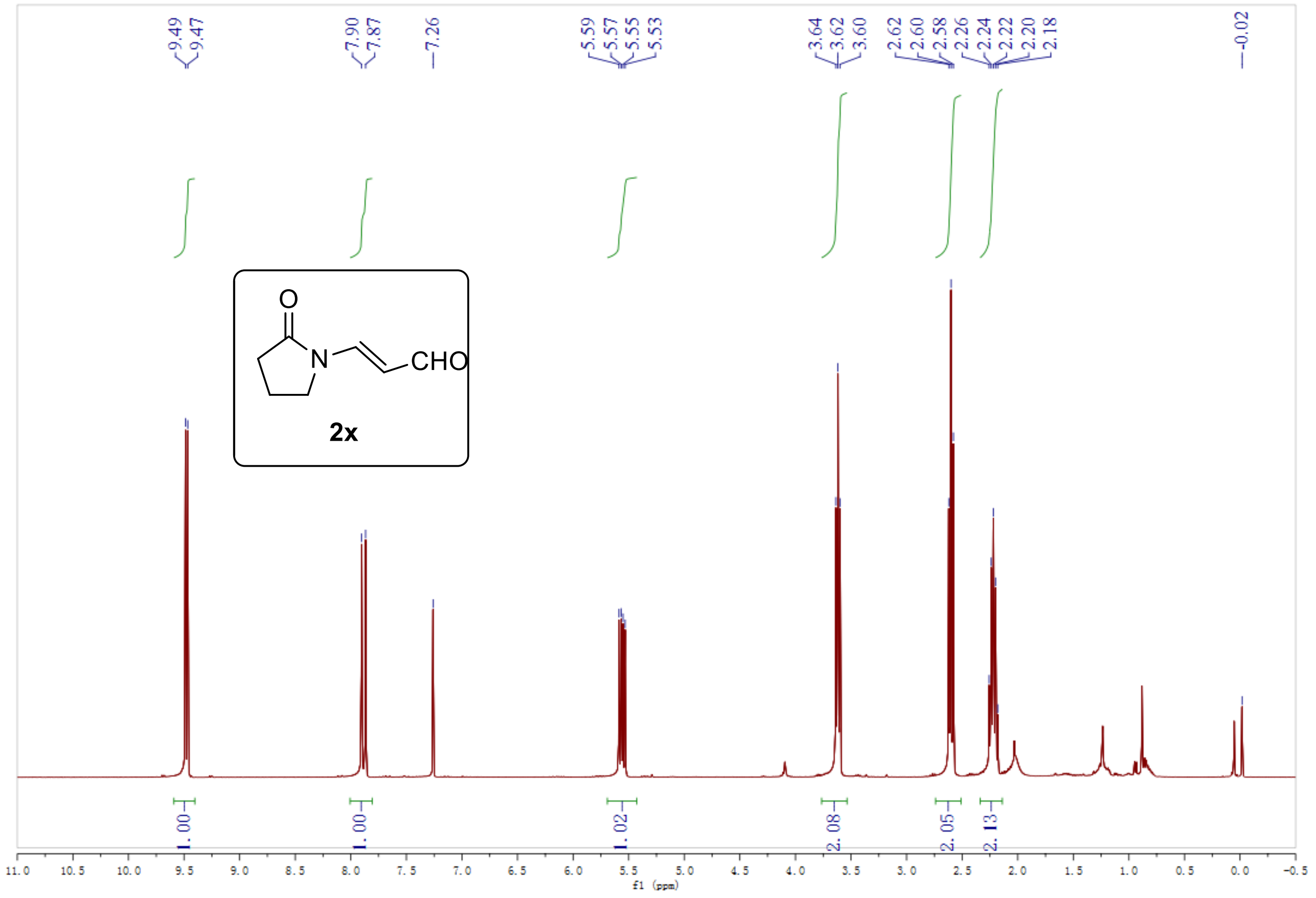
9.49
9.47
7.90
7.87
7.26
5.59
5.57
5.55
5.53
3.64
3.62
3.60
2.62
2.60
2.58
2.26
2.24
2.22
2.20
2.18
-0.02
CHO
N
O
2x
1.00
1.00
1.02
2.08
2.05
2.13
f1 (ppm)

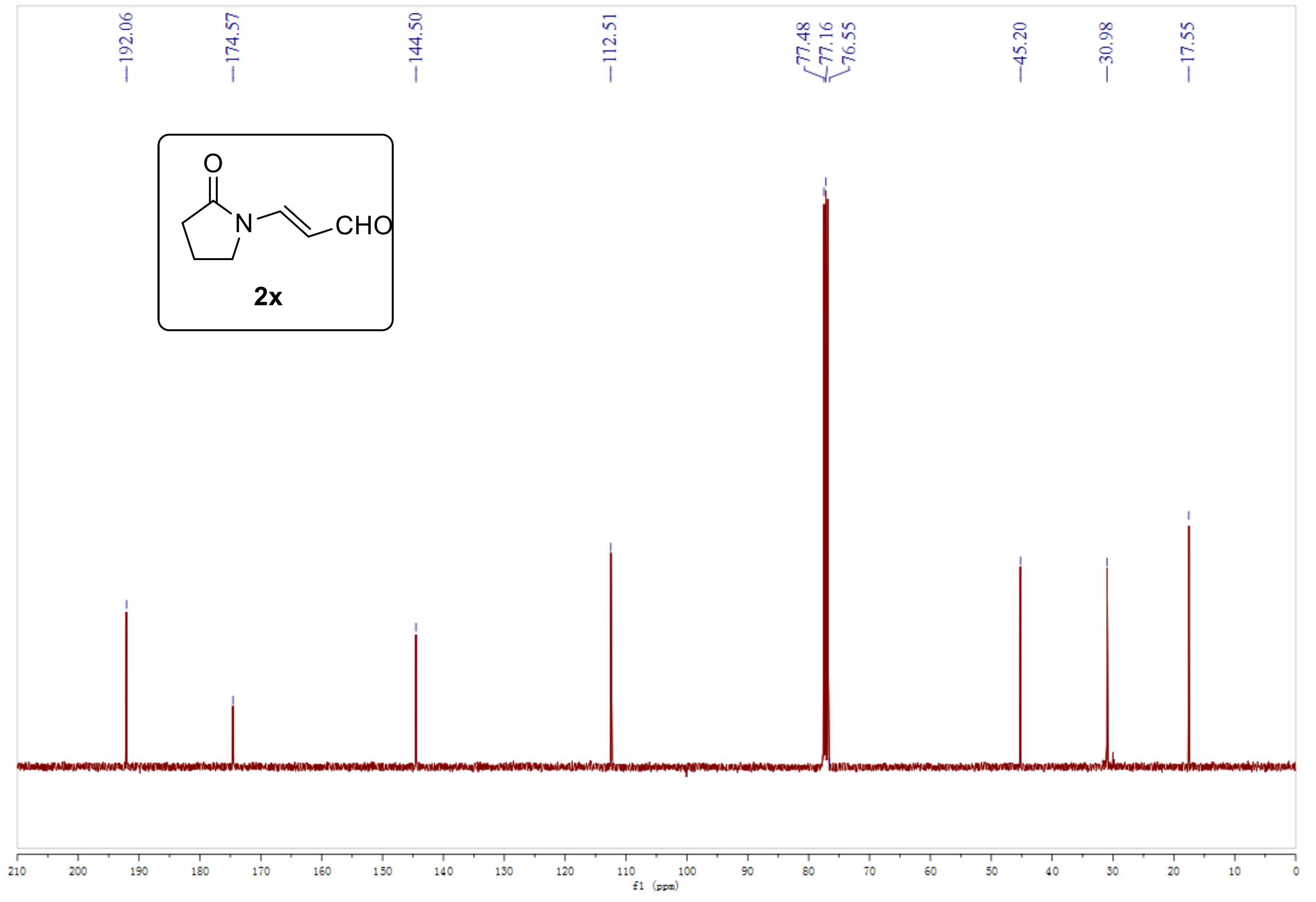
192.06
174.57
144.50
112.51
77.48
77.16
76.55
45.20
30.98
17.55
CHO
N
O
2x
f1 (ppm)

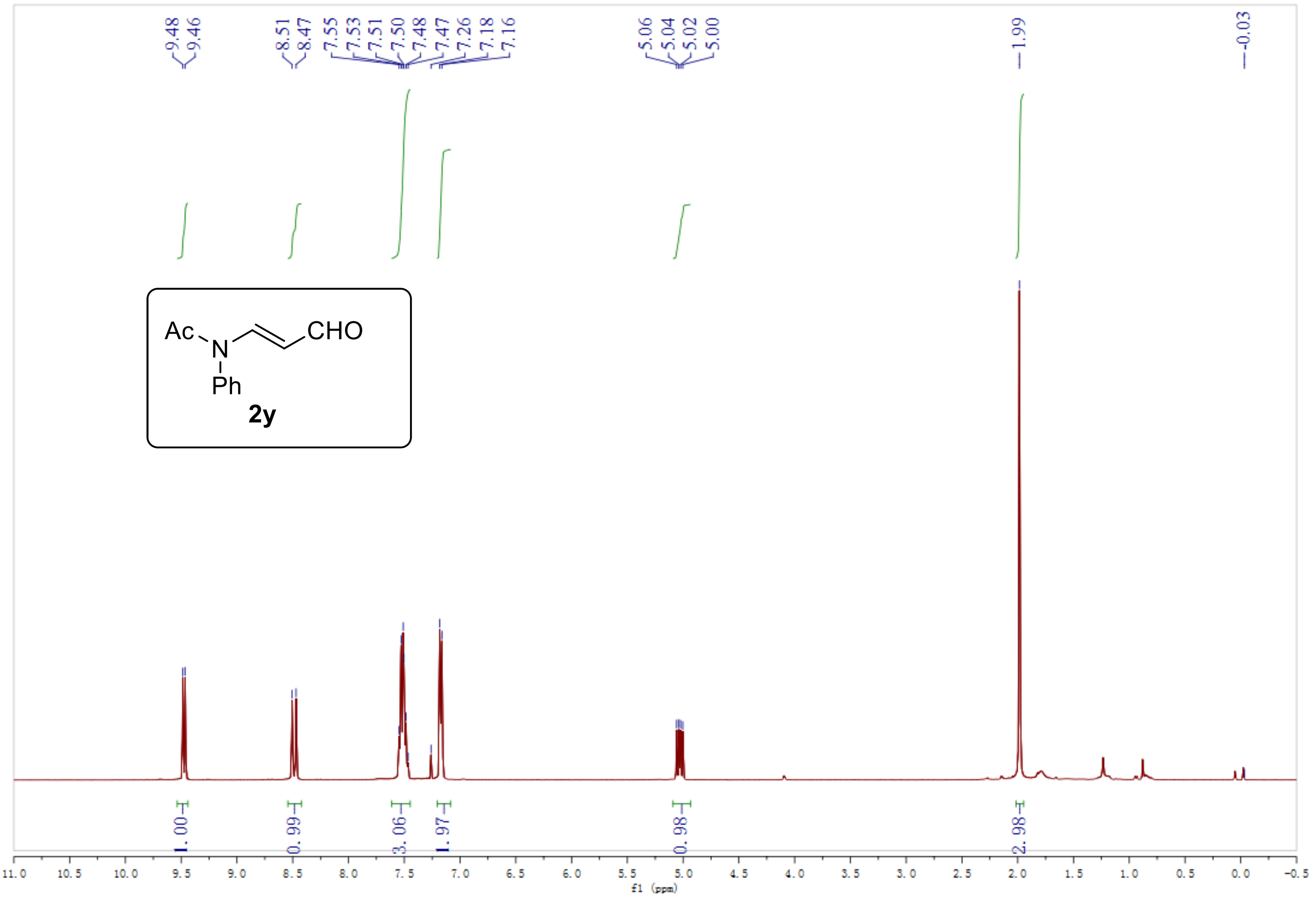
9.48
9.46
8.51
8.47
7.55
7.53
7.51
7.50
7.48
7.47
7.26
7.18
7.16
5.06
5.04
5.02
5.00
1.99
-0.03
Ac
N
CHO
Ph
2y
1.00
0.99
3.06
1.97
0.98
2.98
f1 (ppm)

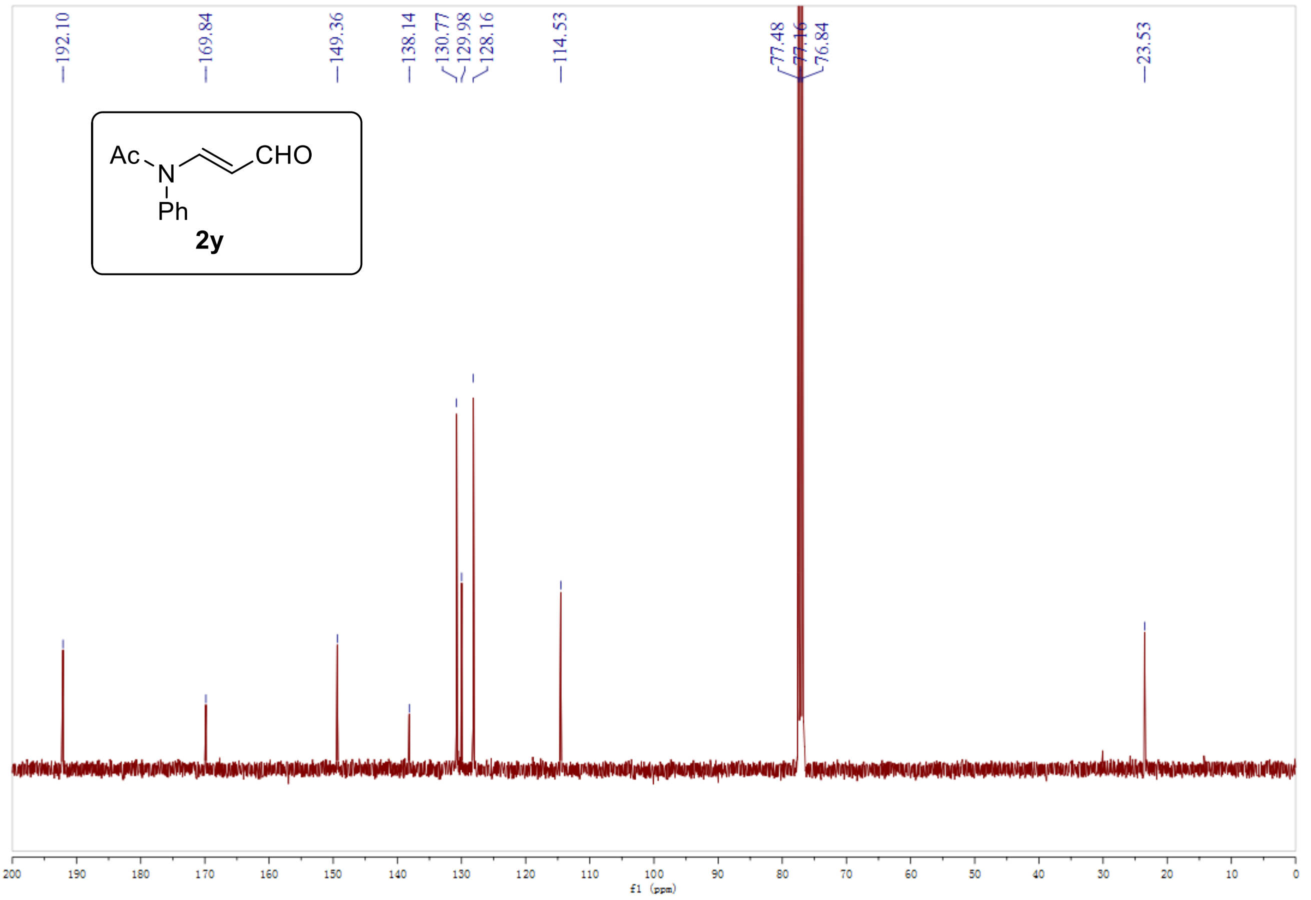
192.10
169.84
149.36
138.14
130.77
129.98
128.16
114.53
77.48
77.16
76.84
23.53
Ac
N
CHO
Ph
2y
f1 (ppm)

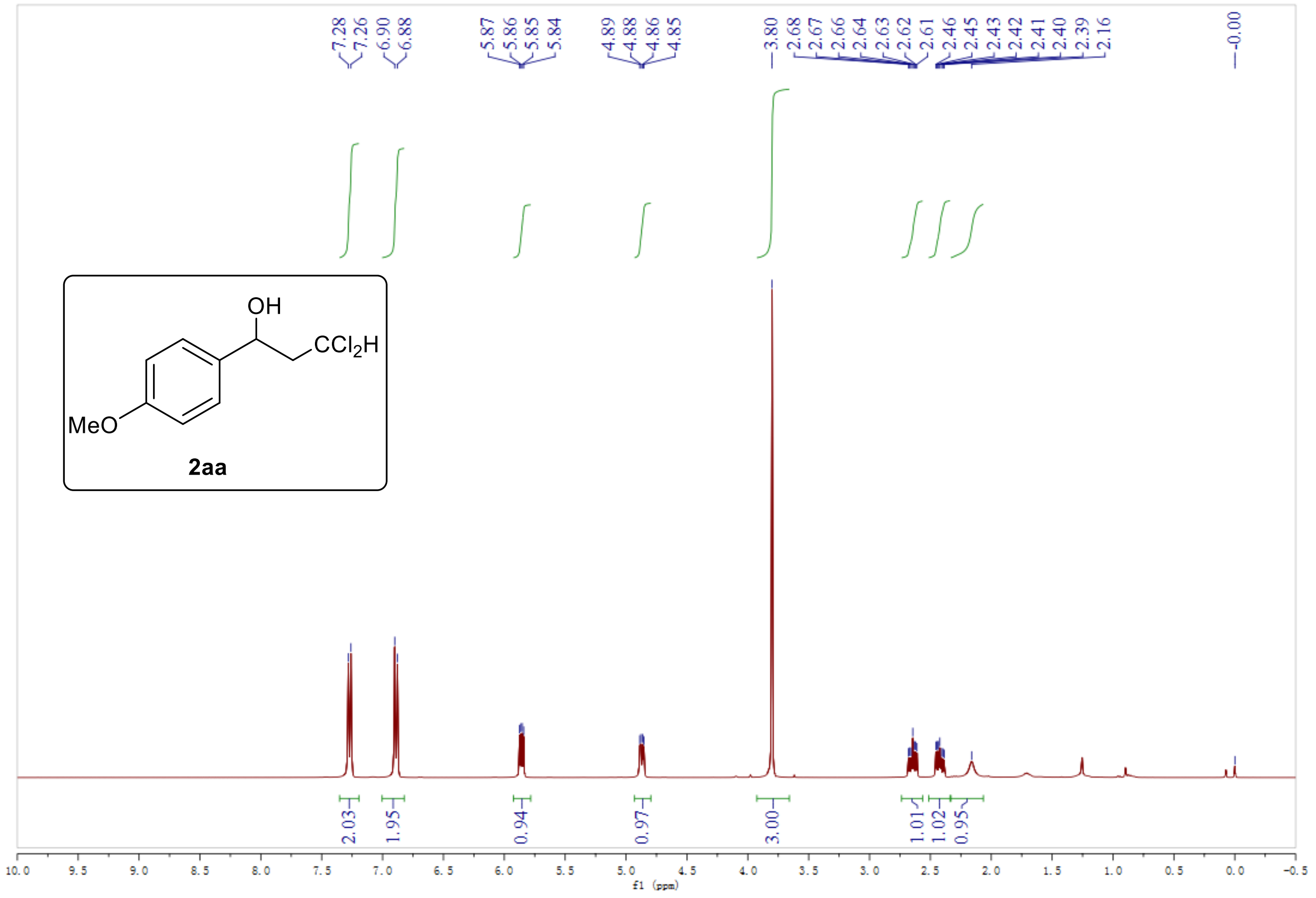
7.28
7.26
6.90
6.88
5.87
5.86
5.85
5.84
4.89
4.88
4.86
4.85
3.80
2.68
2.67
2.66
2.64
2.63
2.62
2.61
2.46
2.45
2.43
2.42
2.41
2.40
2.39
2.16
0.00
OH
CCl2H
MeO
2aa
2.03
1.95
0.94
0.97
3.00
1.01
1.02
0.95
f1 (ppm)

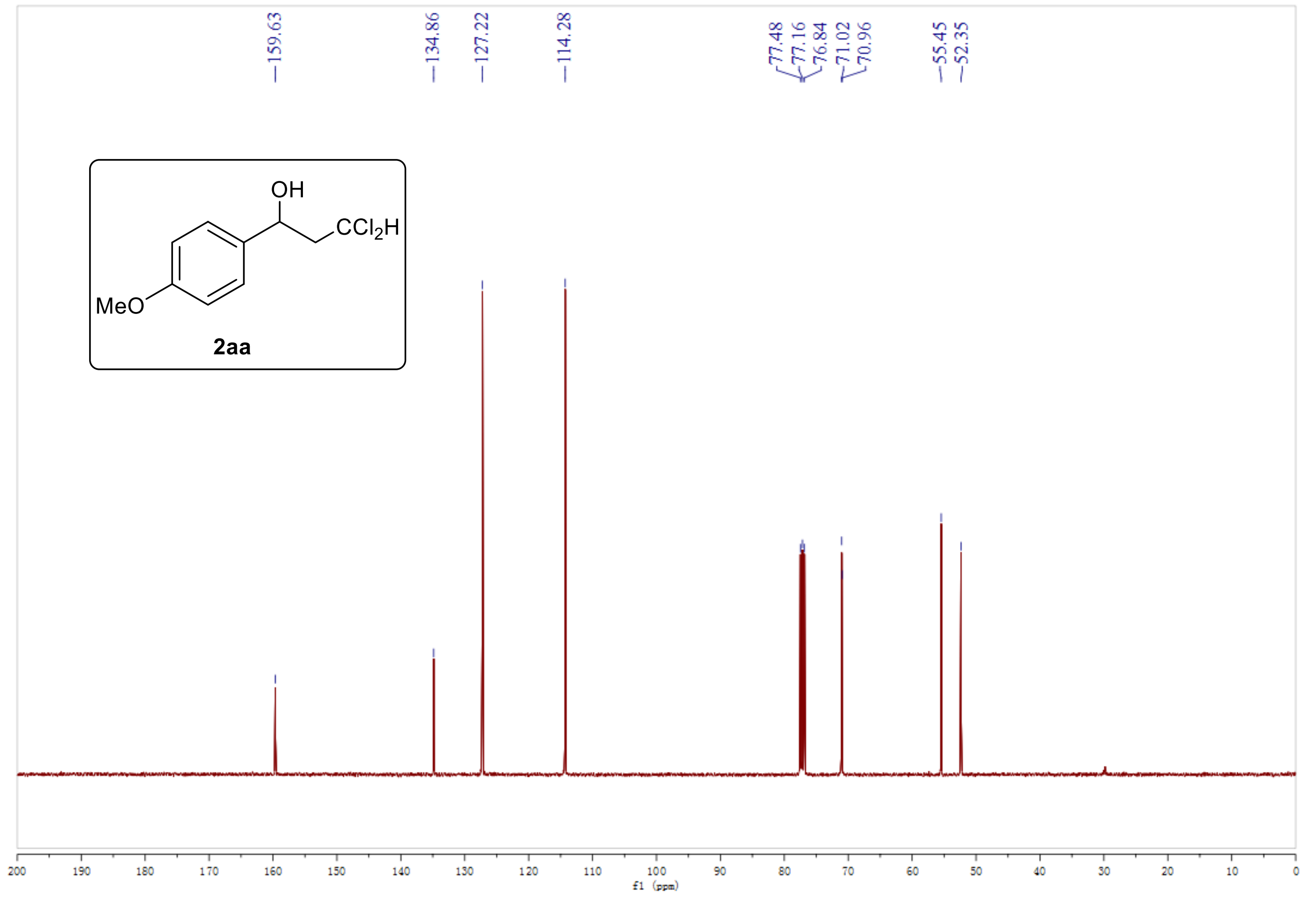
159.63
134.86
127.22
114.28
77.48
77.16
76.84
71.02
70.96
55.45
52.35
OH
CCl2H
MeO
2aa
f1 (ppm)

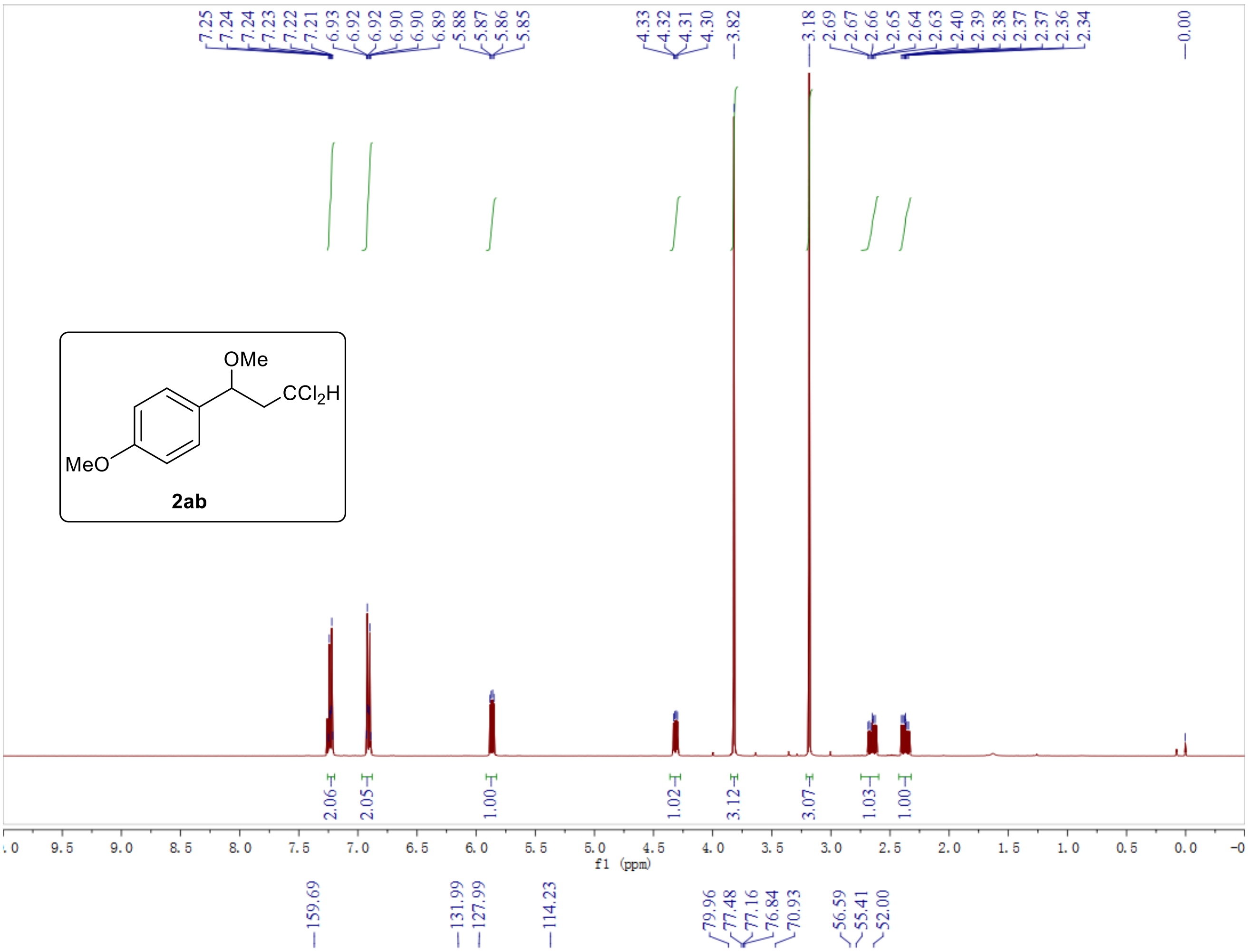
7.25
7.24
7.24
7.23
7.22
7.21
6.93
6.92
6.92
6.90
6.90
6.89
5.88
5.87
5.86
5.85
4.33
4.32
4.31
4.30
3.82
3.18
2.69
2.67
2.66
2.65
2.64
2.63
2.40
2.39
2.38
2.37
2.37
2.36
2.34
0.00
OMe
CCl2H
MeO
2ab
2.06
2.05
1.00
1.02
3.12
3.07
1.03
1.00
f1 (ppm)

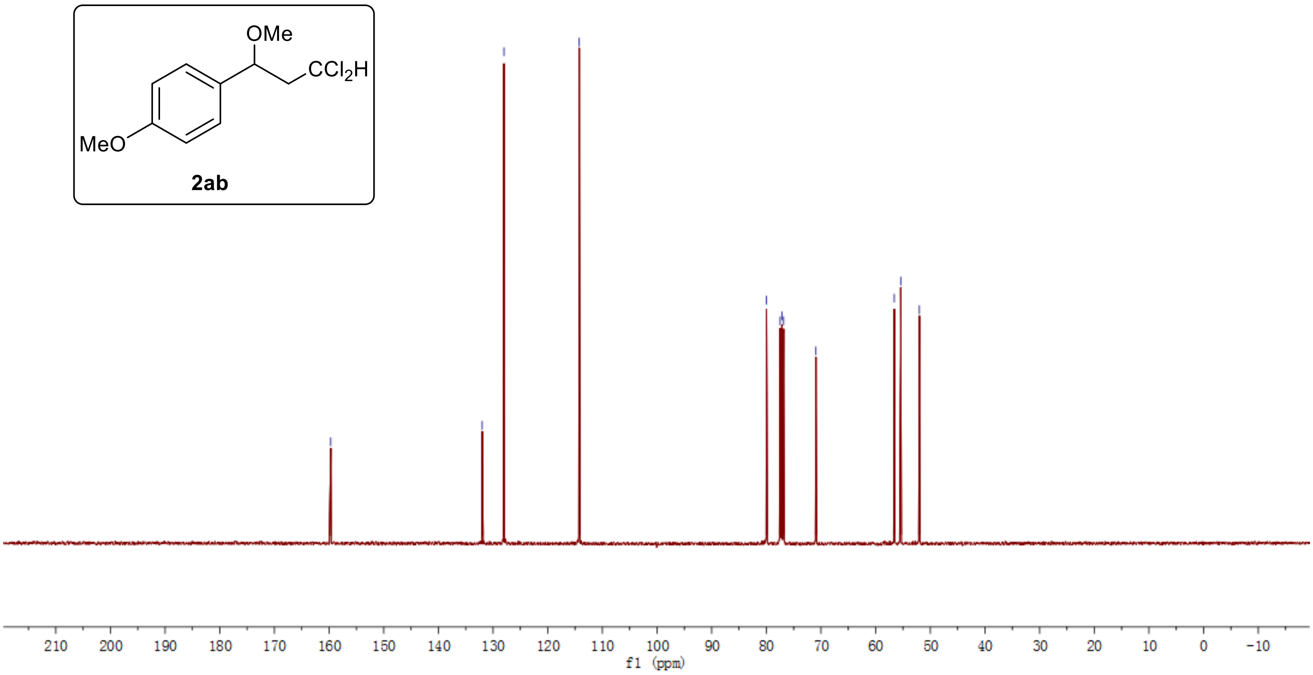
159.69
131.99
127.99
114.23
79.96
77.48
77.16
76.84
70.93
56.59
55.41
52.00
OMe
CCl2H
MeO
2ab
f1 (ppm)

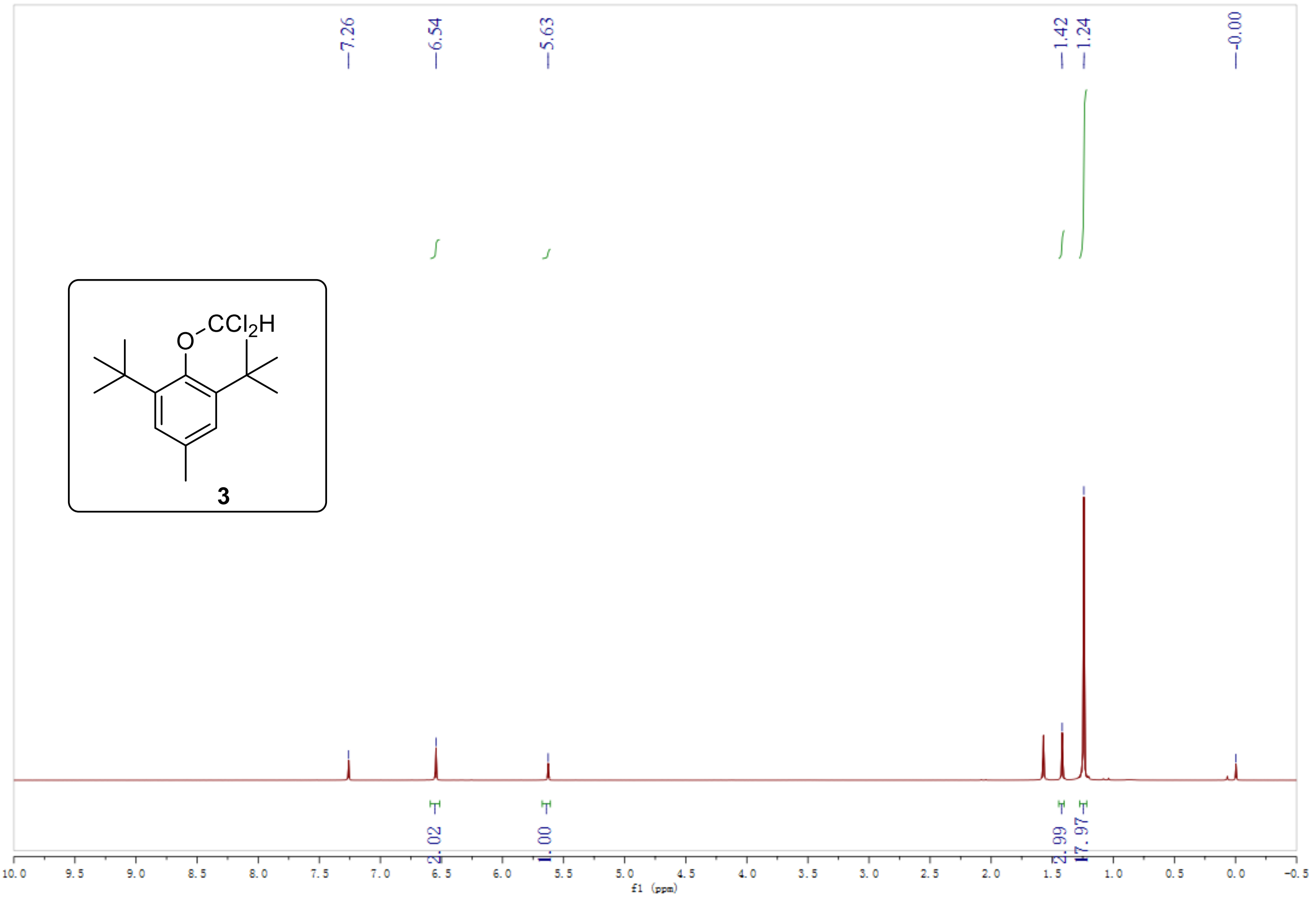
7.26
6.54
5.63
1.42
1.24
0.00
O
CCl2H
3
2.02
1.00
2.99
17.97
f1 (ppm)

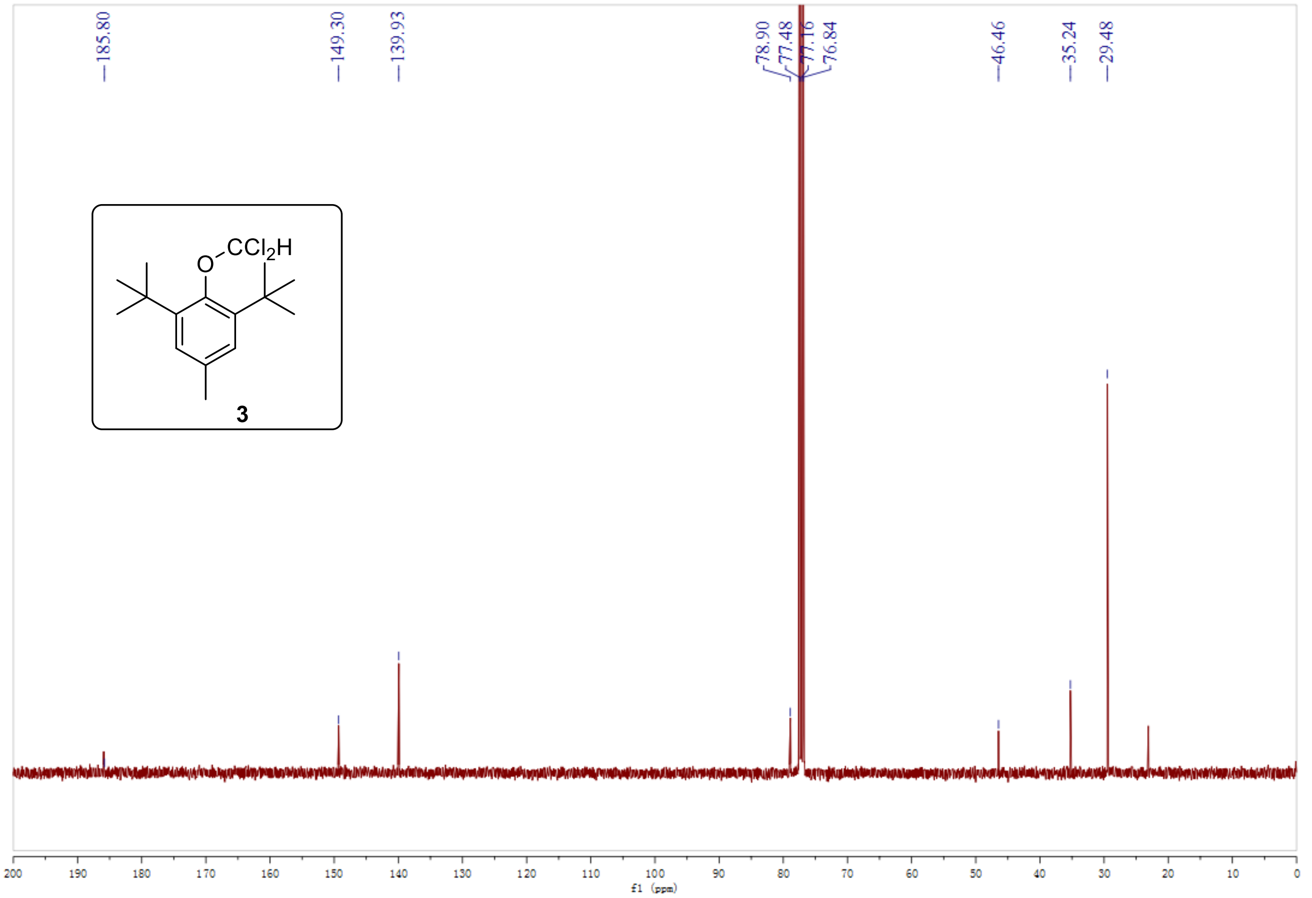
185.80
149.30
139.93
78.90
77.48
77.16
76.84
46.46
35.24
29.48
O
CCl2H
3
f1 (ppm)

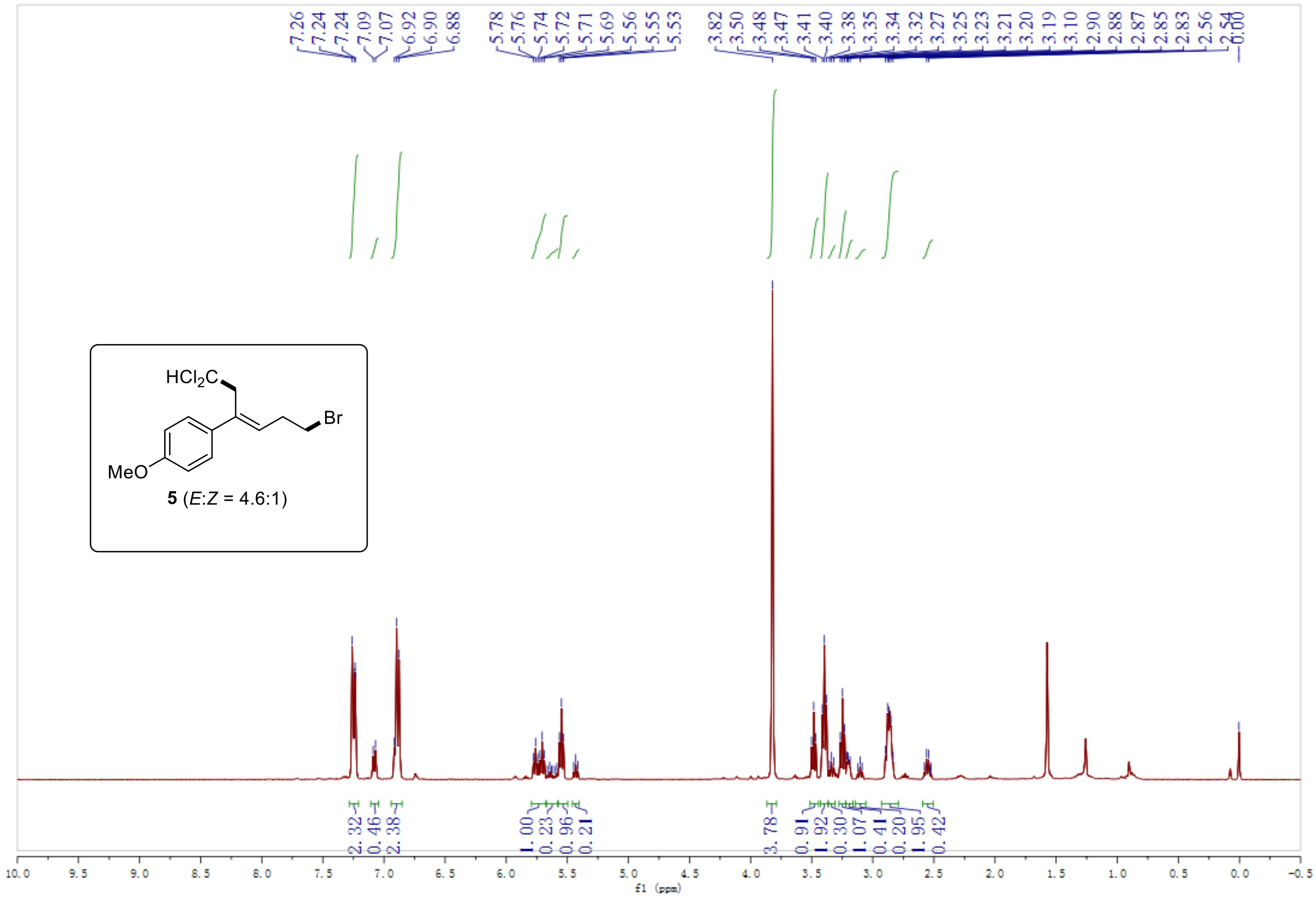

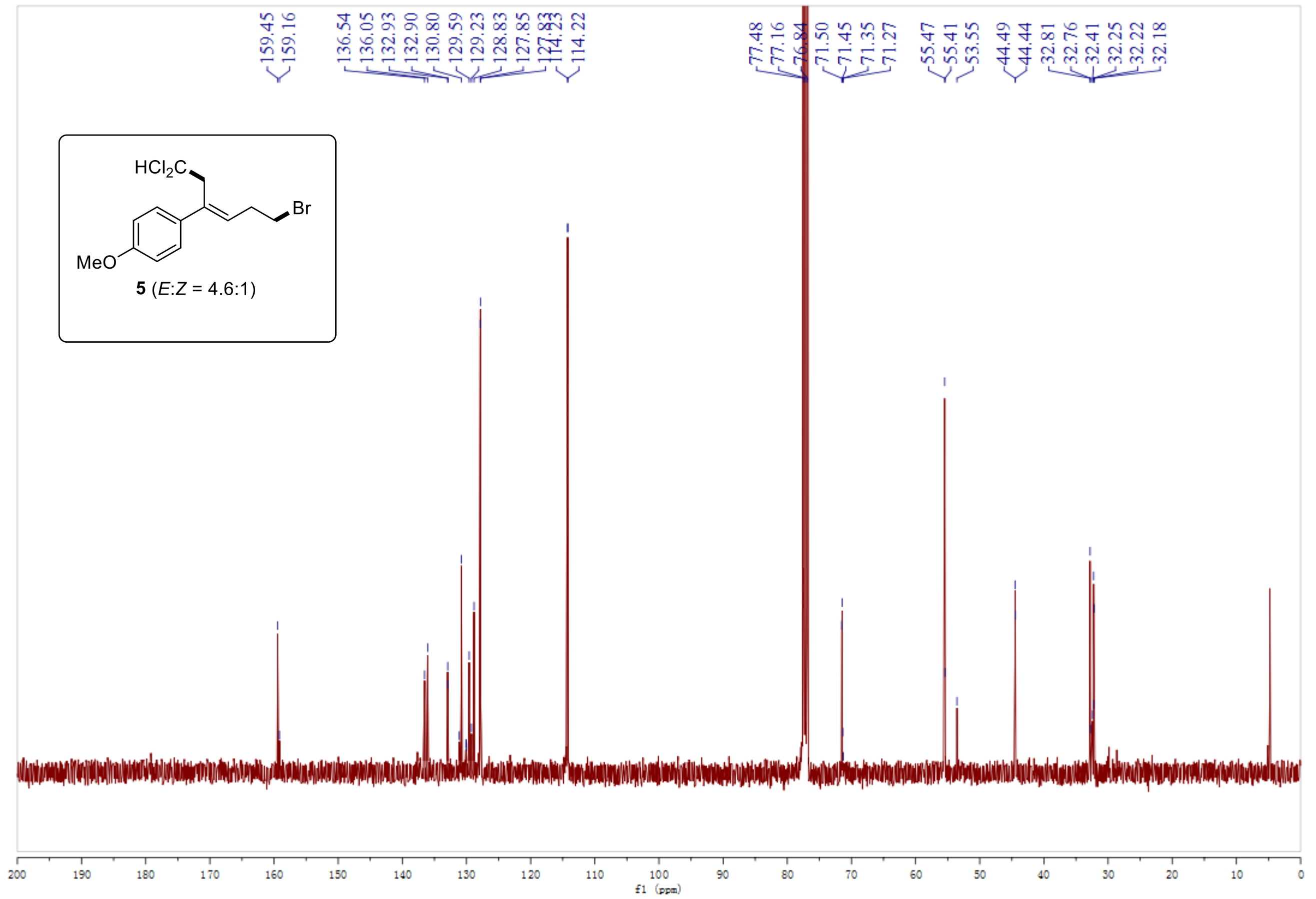

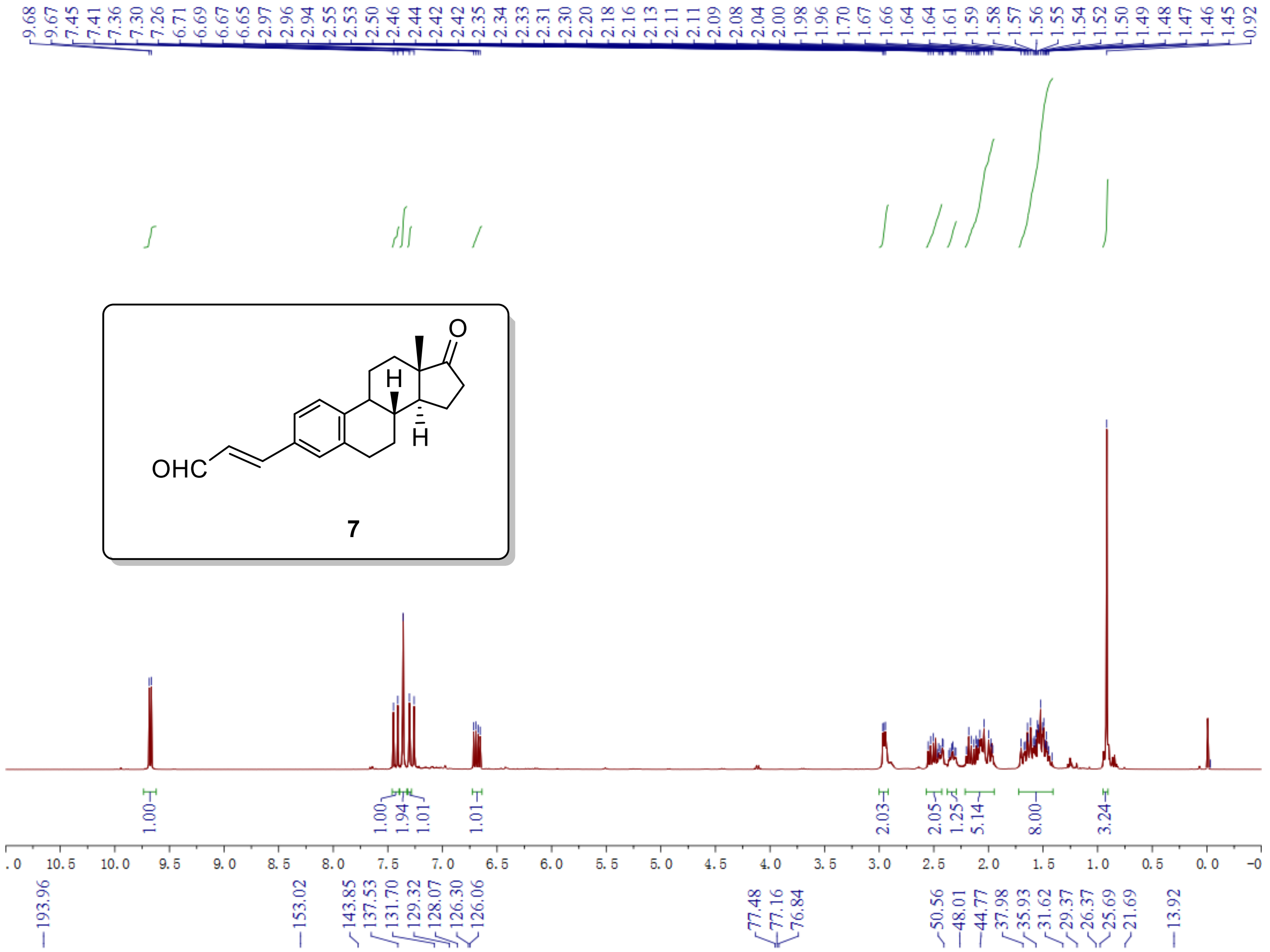

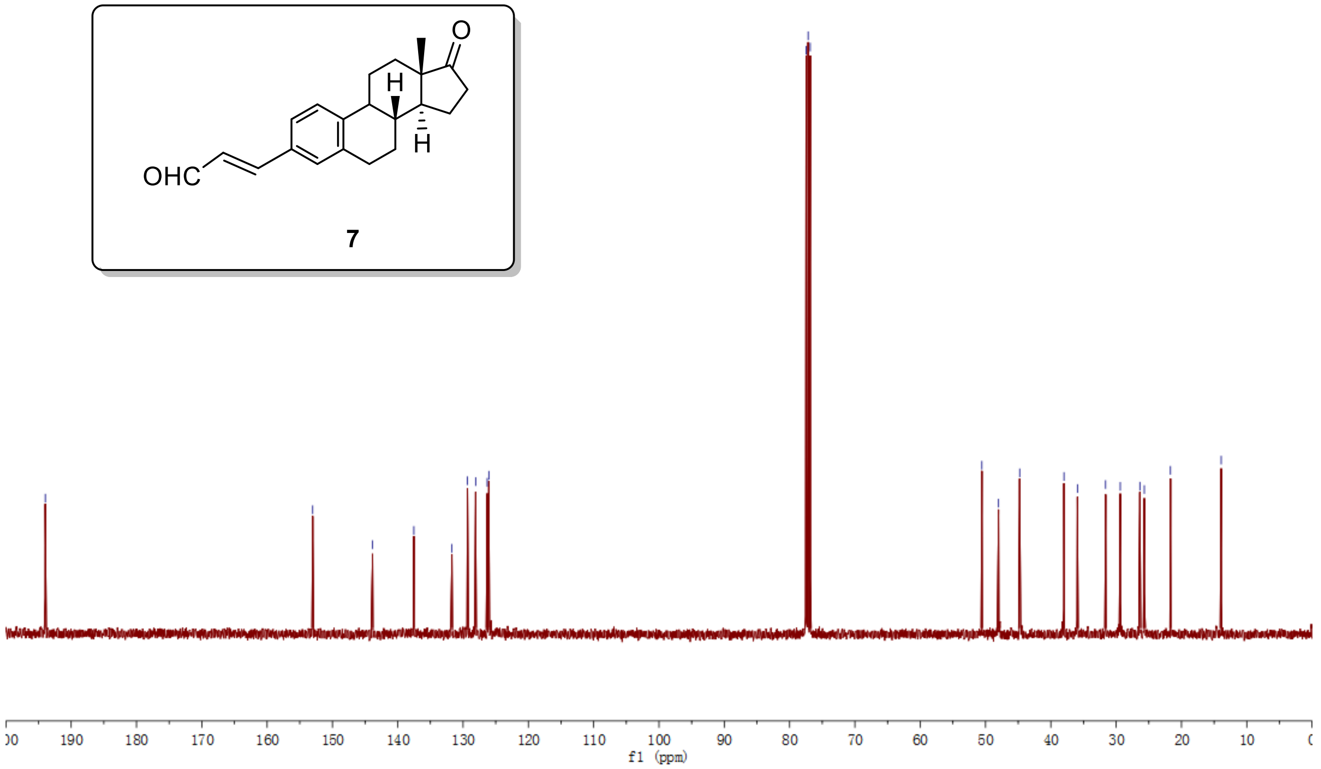

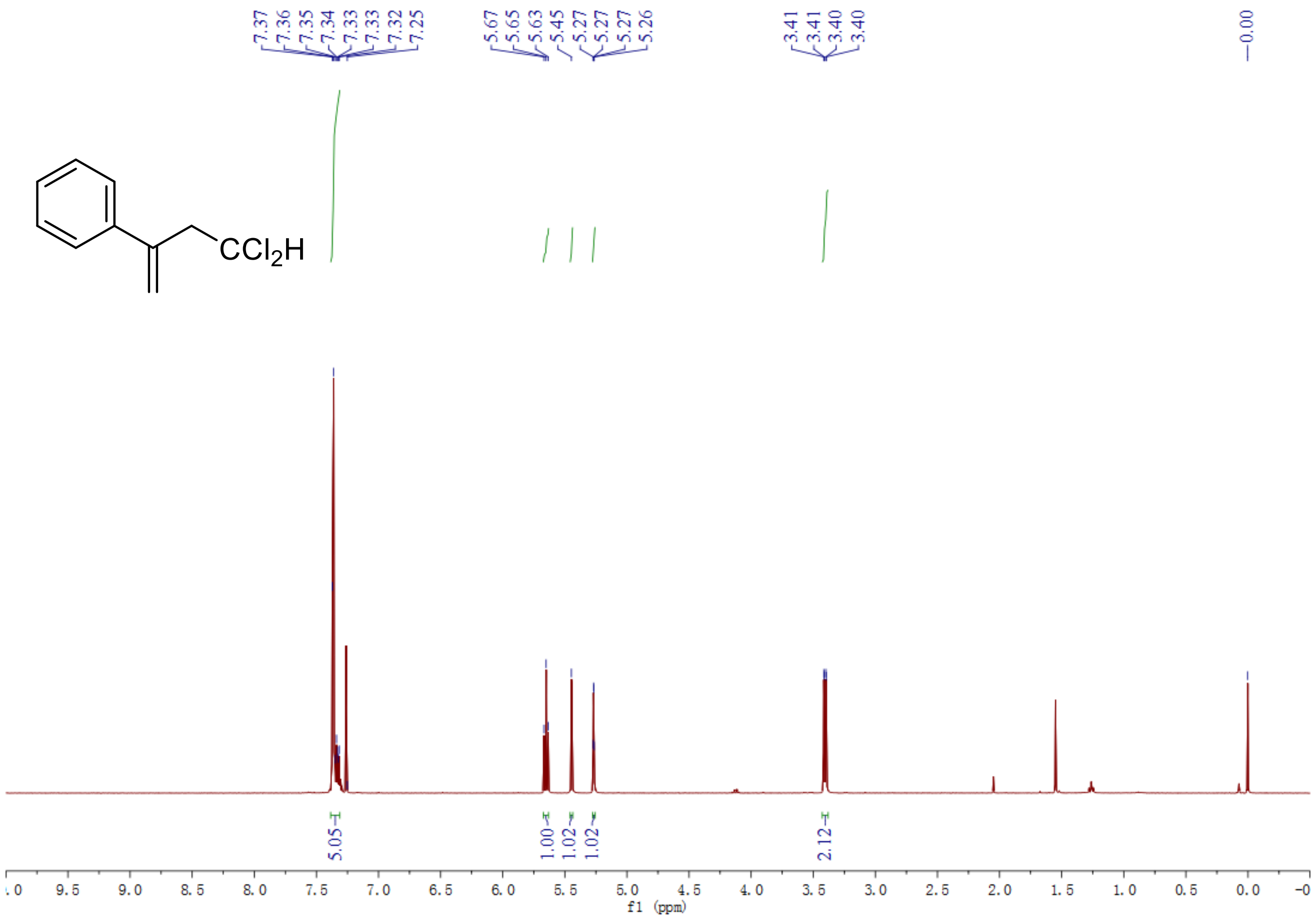

7.37
7.36
7.35
7.34
7.33
7.33
7.32
7.25
5.67
5.65
5.63
5.45
5.27
5.27
5.27
5.26
3.41
3.41
3.40
3.40
0.00
CCl2H
5.05
1.00
1.02
1.02
2.12
f1 (ppm)

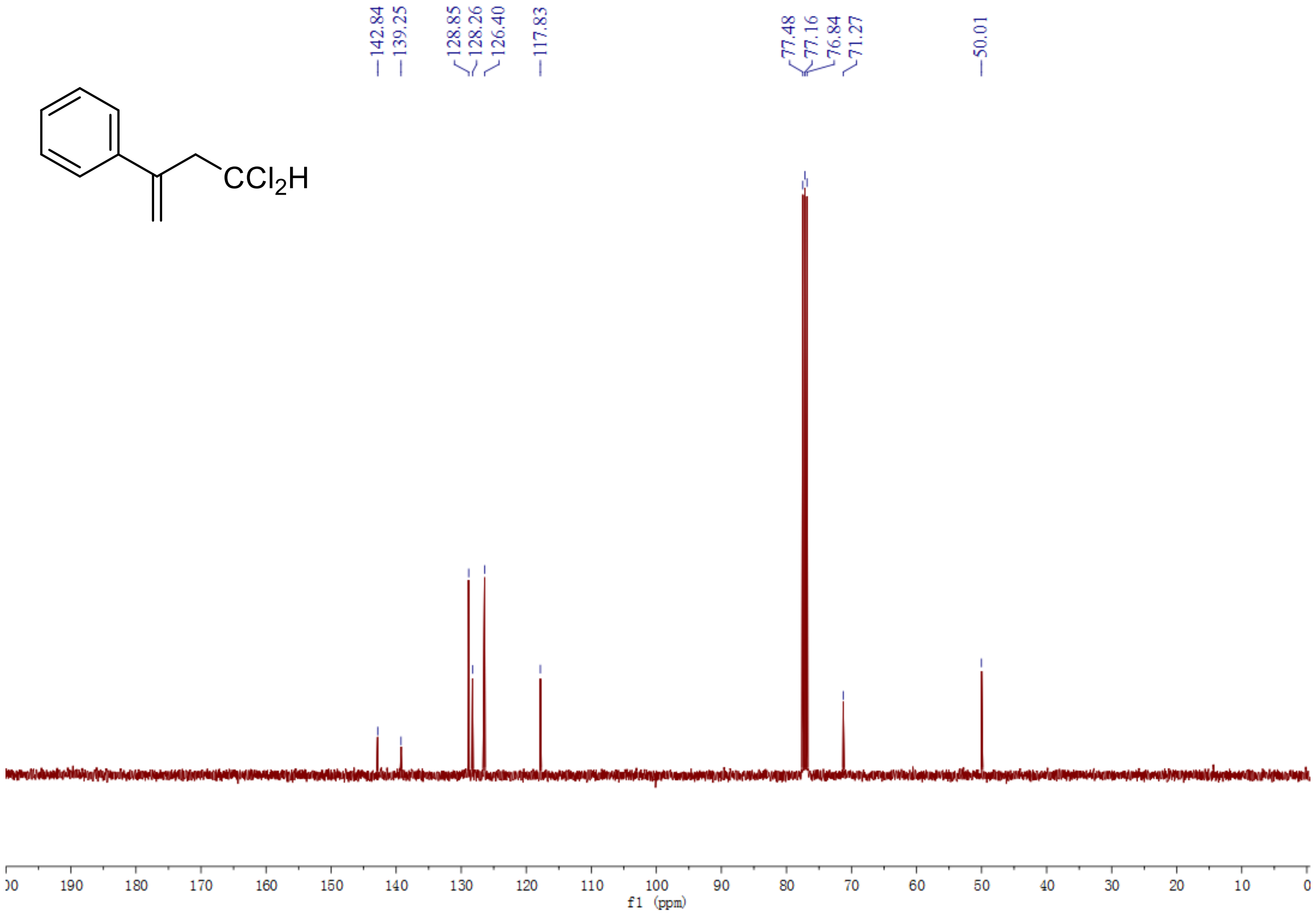

142.84
139.25
128.85
128.26
126.40
117.83
77.48
77.16
76.84
71.27
50.01
CCl2H
f1 (ppm)
